# Resilience and livestock adaptations to demographic growth and technological change: A diachronic perspective from the Late Bronze Age to Late Antiquity in NE Iberia

**DOI:** 10.1371/journal.pone.0246201

**Published:** 2021-02-17

**Authors:** Ariadna Nieto Espinet, Thomas Huet, Angela Trentacoste, Silvia Guimarães, Hector Orengo, Silvia Valenzuela-Lamas

**Affiliations:** 1 Consejo Superior de Investigaciones Científicas (CSIC), Institució Milà i Fontanals, Archaeology of Social Dynamics, Barcelona, Spain; 2 Archéologie des Sociétés Méditerranéennes, CNRS-UMR 5140, Montpellier, France; 3 Institute of Archaeology, University of Oxford, Oxford, United Kingdom; 4 CIBIO-InBIO, Centro de Investigação em Biodiversidade e Recursos Genéticos, Universidade do Porto, Vairão, Portugal; 5 Institut Català d’Arqueologia Clàssica (ICAC), Tarragona, Spain; Universita degli Studi di Milano, ITALY

## Abstract

There are strong interactions between an economic system and its ecological context. In this sense, livestock have been an integral part of human economies since the Neolithic, contributing significantly to the creation and maintenance of agricultural anthropized landscapes. For this reason, in the frame of the ERC-StG project ’ZooMWest’ we collected and analyzed thousands of zooarchaeological data from NE Iberia. By considering these data in comparison with ecological indicators (archaeobotanical remains) and archaeological evidence (settlement characteristics and their distribution) this paper seeks to characterize changes in animal production and the relationship between people, livestock, and their environment. These methods allow for an investigation of the topic at different scales (site, zone, territory) with a broad diachronic perspective, and for consideration of orography and cultural traditions alongside climatic factors. Through this integration of various streams of evidence, we aim to better understand the structure of ancient economic systems and the way they conditioned human decision-making on animal production. Results show a shifting relationship with the territory between the Bronze Age and Late Antiquity, in which market requirements and an economic model with a higher degree of integration increasingly influenced husbandry strategies. These processes are reflected in changes in land use and forms of territorial occupation, although along different rhythms and trajectories.

## 1. Introduction

Europe experienced significant economic and political changes between Late Prehistory and the Classical period [1–6]. Complex societies with a strong territorial component developed during the Late Bronze Age and the Early Iron Age in the north-west of the Mediterranean basin (northeastern Iberia and the area of the Gulf of Lion), which subsequently led to the formation of proto-statal structures during the Middle Iron Age [1,7–14]. In this area, the culturally and politically atomized landscape attested during the Iron Age was transformed following the Roman conquest, and further modified through its integration within the Roman Empire, which grouped many communities under a previously unseen level of large-scale economic connectivity [15–20]. This first ’globalized system’ on a Mediterranean scale experienced a major crisis during the 6^th^ c. AD, leading to its deconstruction and a re-localization of production, which retracted and became more autarchical during Late Antiquity [17,21–23].

In the North-East (NE) of the Iberian Peninsula, the transition from the Bronze to the Iron Age marked a significant moment of change in settlement structure. In association with the spread of iron technology during the 8^th^ c. BC, scattered huts made of perishable materials were replaced by stone-built settlements located on hilltops [24–26]. The spread of iron technology for tool production is thought to have promoted greater cereal production and a demographic increase, which subsequently led to increased pressure over resources, potentially inciting territorial conflicts, fortification and social stratification [25–28]. The impact of these phenomena on animal husbandry is not yet fully understood, but they appear to have had a significant influence on livestock body size, considering the general small size of animals attested during the Bronze and Iron Ages [29–31]. Animal size subsequently increased during the Roman period, as documented in many parts of Europe [31–46]. The factor(s) driving animal size changes through time are not clearly understood yet, but different models have been proposed to explain the size reduction during Late Prehistory including: the controlled selection of smaller individuals to produce more manageable animals [47], consequence of climatic changes [48], and an intensification of sub-adult breeding [49]. A further set of proposals has been advanced in relation to increases in livestock size during the Roman period, especially the introduction of new morphotypes [31]. Across time, factors for size change were probably influenced by the socio-political context in which animals were raised, and the different potentials and challenges provided by it [45,50–52]. Although increasingly recognized as a major determinant in shaping husbandry strategies, the interaction between animal management and the socio-political context in which these activities took place is rarely evaluated systematically. Previous synthetic works for the region have focused on smaller areas or time periods [53,54], but ours is the first study to combine species frequencies and detailed biometric analysis over protohistory and classical times, and to contextualize these data with larger scale environmental and archaeological evidence.

This paper aims to contribute to this debate by providing an integrated study that presents zooarchaeological data from the NE of the Iberian Peninsula from the Late Bronze Age to Late Antiquity. The zooarchaeological results are contextualized using other streams of archaeological evidence, i.e. settlement patterns, material culture, archaeobotanical data. The ultimate goal is to analyze to what extent ecology and / or socio-political factors may influence animal husbandry in terms of species selection and animal size.

## 2. Geographical setting

**Fig 1:**
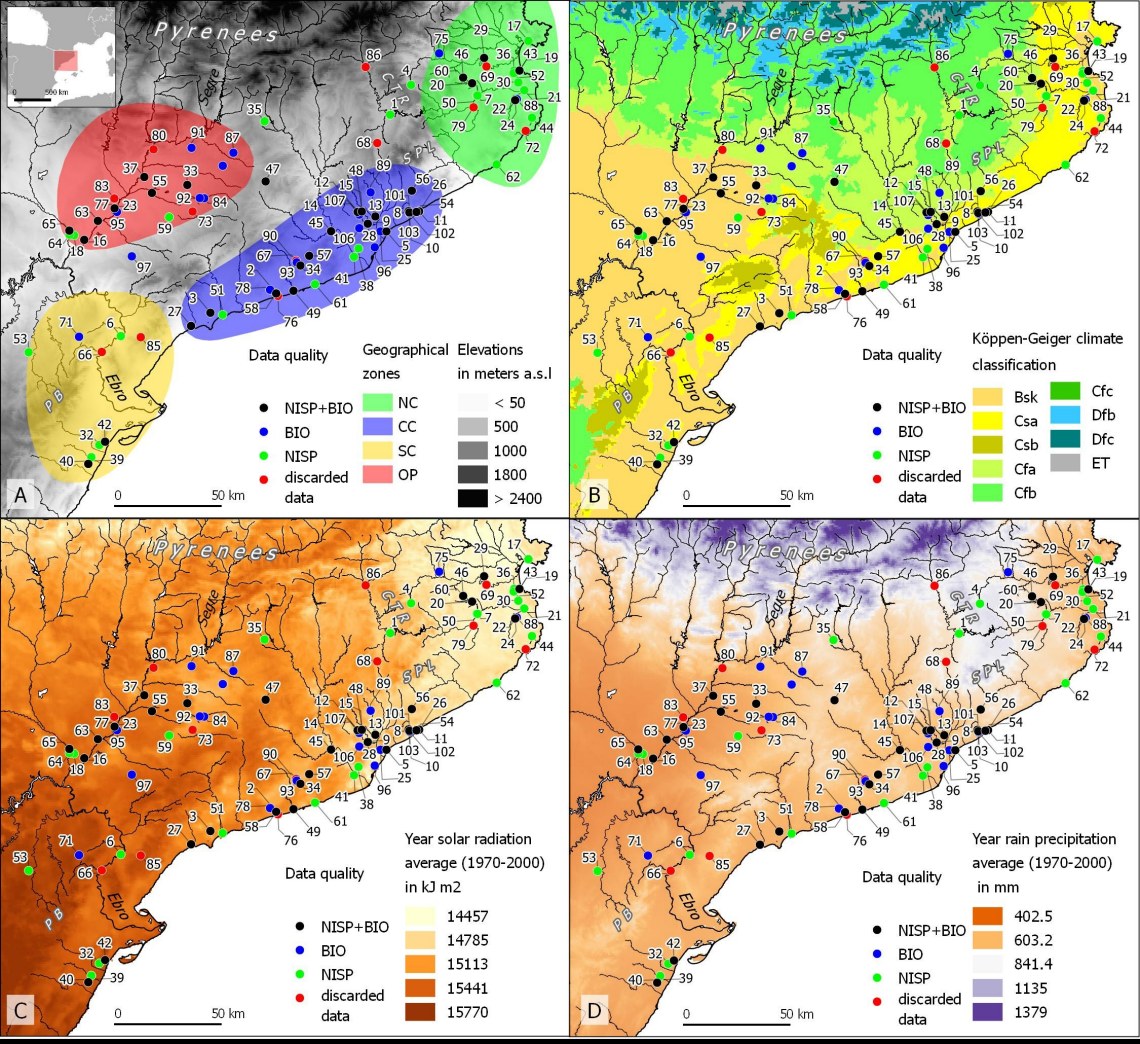
Map of NE Iberia with locations of the studied sites. A) Location of geographical areas (NC: North Coast, CC: Central Coast, SC: South Coast, OP: Occidental Plain) with sites colored depending of archaeozoological data availability (NISP: Counts by species, BIO: Biometrical data) with elevation map reprinted using open data from Geofabrik and the U.S. Geological Survey [55,56]; B) Köppen-Geiger climate classification reprinted from open data Zenodo [57]. The classification is based on threshold values and seasonality of monthly air temperature and precipitation. Considering vegetation as “crystallized, visible climate” C) Averages of year solar radiations between 1970–2000 [58]. D) Averages of rain precipitations between 1970–2000 [58] reprinted from WorldClim (CC-BY SA 4.0). List of sites: 1. **Esquerda**; 2. Alorda Park; 3. Antigons; 4. Aubert; 5. Baetulo; 6.Barranc de Gàfols; 7. Bosc del Congost; 8. Burriac; 9. Ca n’Oliver; 10. Can Bartomeu; 11. Can Cruzate; 12. Can Feu; 13. Turó de la Rovira; 14. Can Gambús 1; 14. Can Gambús 3; 15. Can Roqueta_DIASA; 15. Can Roqueta CRV; 15. Can Roqueta TR; 16. Carretelà; 17.Ciutadella de Roses; 18. Cova Punta Farisa; 19. Empúries; 21. Fonollera; 22. Gou Batlle; 23. Ilerda 1; 23. Ilerda; 23. Ilerda 2; 24. Illa d’en Reixac; 25. Puig Castellar; 26. Iluro_VI_VII; 27. La Llosa; 28. Mallols; 29. Mas Castellar rural; 29. Mas Castellar fortificat; 29. Mas Castellar; 30. Mas Gusó; 32. Moleta del Remei 1_1; 33. Molí d’Espígol; 34. Olèrdola; 35. Olius; 36. Olivet d’en Pujol; 37. La Pedrera; 38. Penya del Moro; 39. Puig de la Misericordia; 40. Puig de la Nau; 41. Sant Boi_Pl Constitució; 42. Sant Jaume Mas d’en Serrà; 43. Sant Martí Empúries; 44. Sant Sebastià de la Guarda; 45. Santa Margarida; 46. Saus; 47. Sigarra; 48. Sitges UAB; 49. Solana; 50. St. Julià de Ramis; 51. Tarraco; 52. Tolegassos; 53. Torre Cremada; 54. Torre Llauder; 55. Tossal Molinet; 56. Turó del Vent; 57. Turó Font de la Canya; 58. Vilarenc; 59. Vilars; 60. Vilauba; 61. Vil·la Vinyet; 62. Vil·la dels Ametllers; 63. Vilot de Montagut; 64.Vincamet; 65. Zafranales; 68. Camp de les Lloses; 69. Camp Gran-Camp d’en Pitu Porrusia; 70. Can Cruzate; 71. Coll del Moro; 72. Collet de Sant Antoni; 73. Estinclells; 74. Fonollera; 76. Les Guardies; 77. Lleida_Carrer Bafart; 78. Mas d’en Gual; 79. Mas Xirgù; 80. Monterò; 82. Puig de la Misericordia; 84. Rosella; 83. Roques del Sarrò; 85. Tossal del Moro de Pinyeres; 86. Turo del Calvari; 87. Puig Castellar Biosca; 88. Puig de Sant Andreu; 89. Torre Roja; 91. Hereuet; 92. Missatges; 94. Iesso; 95. Vil·la Torre Andreu; 97. Vilans de Reig; 101. Can Mateu; 102. Can Bernat; 103. Can Rodon; 106. Can Sant Joan; 107. Mas Duran.

This study considers four areas within the NE Iberian Peninsula, present-day Catalonia (Fig 1): the North Coast (NC), Central Coast (CC), South Coast (SC), and Occidental Plain (OP) (Fig 1A). The region has several rivers connecting the Pyrenees with the Mediterranean shore. The biggest one is the Ebro River, which is partly navigable, and links the Occidental Plain (OP) to the South Coast (SC).

While present climate is not directly applicable to the past, especially over the millennia considered in this study, it nevertheless provides an indication of potential relative differences in temperature (Fig 1B), precipitation (Fig 1D), and solar radiation (Fig 1C). The shore of the **North Coast (NC)** is described as an arid cold steppe (Bsk) in the Köppen-Geiger climate classification [55]. Its hinterland, about 20 km from the coastline, is temperate, with a dry and hot summer (Csa). The Catalan Transversal Range (ca. 500–900 m.a.s.l.), located in the hinterland of the NC, is the wettest and most cloudy of the areas considered, with a relatively annual high rate of rainfall (841–1135 mm) (Fig 1D), and a relatively low solar radiation (14457–14785 kJ m^2^) (Fig 1C). The rivers of La Muga, Fluvià and Ter connect the Catalan Transversal Range to the coast. The **Central Coast (CC)** has a warmer climate on average compared with the NC, with dry and hot summers (Csa) in the coastal valleys, and a temperate climate, with no dry season and a hot summer (Cfa), in the neighboring mountain range (Serralada Prelitoral, 500–1000 m.a.s.l.). In addition, an arid cold steppe climate area (Bsk) is located in the southern part of CC (Fig 1C). The **South Coast (SC)** area is mostly composed of a semi-arid cold steppe climate (Bsk) (Fig 1B). A small part of this zone shows a temperate climate with dry and hot summers (Csa). Only in the Ports de Beseit mountain range (ca. 1000–1200 m.a.s.l.) is a dry and warm summer found (Csb). The SC has the highest annual average of solar radiation of the four zones studied (above 15441 kJ m^2^) (Fig 1C), and the annual rainfall is similar to the North and Central Coast (Fig 1D). Finally, the **Occidental Plain (OP)** shows mostly a semi-arid and cold steppe climate (Bsk) in the lower part of the Segre river (Fig 1B), and a temperate hot summer (Cfa) in the middle Pre-Pyrenees mountain range (ca 400–600 m.a.s.l.). This area is the driest of the four studied, with ca. 420 mm of annual rainfall (Fig 1C and 1D).

## 3. Archaeological context: Changes in settlement patterns and the political organization in NE Iberia

A detailed analysis of settlement dynamics in the North-East of the Iberian Peninsula during Late Prehistory and early historical times (Table 1) is beyond the scope of this paper, but a general overview is necessary to contextualize the zooarchaeological data. The following paragraphs present the main changes in settlement dynamics and site types according to synthetic works and the accepted terminology for the region (Table 2).

**Table 1:** Diachrony considered.

Periods	Subperiods	Abb.	Chronology
**Bronze Age**	Late Bronze Age	LBA	13th– 9^th^ c. BC
**Iron Age**	Early Iron Age	EIA	8th– 7^th^ c. BC
	Middle Iron Age 1	MIA1	6th– 5^th^ c. BC
	Middle Iron Age 2	MIA2	4th– 3^rd^ c. BC
**Roman period**	Roman Republic	RR	2nd– 1^st^ c. BC
	Early Roman Empire	ERE	1^st^– 3^rd^ c. AD
	Late Roman Empire	LRE	4th– 5^th^ c. AD
**Late Antiquity**	Late Antiquity	LA	5th– 6^th^ c. AD

**Table 2:** Description of the types of site. For details see [27,28,59–62].

Categories	Abb.	Description
Aristocratic residence	AR	Small nuclei with solid and complex fortifications
Closed settlement	CS	Semi-detached houses with perimeter wall and central space for community use
Ecclesiastical	E	Rural churches without direct links with a known village, but which bring together a scattered population
Fortified settlement	FS	Houses surrounded by a wall and / or other defensive elements (towers, ditch, etc.), with a total surface varying from c. 0.5 Ha to c. 2 Ha
Open settlement	OS	Houses built with perishable materials and without fortifications
Periurban	PU	Houses or neighborhoods located on the outskirts of the wall of an urban nucleus
Rural settlement	RS	Detached houses with productive facilities and without fortification
Rural settlement with silo field	RSSF	Rural settlement with an associated extension of storage pits (silos). See ‘Rural settlement’
Scattered village	SV	Settlement with a high scattering of dwellings, no complexity with regard to space, and an expansive use of the space. Each productive units possesses a certain degree of autonomy
Scattered village with silos field	SVSF	Scattered village with an associated extension of storage pits (silos). See ‘Scattered village’
Shelter	S	Natural cover with a seasonal or permanent occupation
Silo field	SF	Wide extension of storage pits (silos) in which no habitation structures have been found
Urban	U	Settlements with a complex plot (domestic/public buildings, streets, etc.) and activities (high density/diversity of social interactions, economic production, etc.), larger than 2 Ha—first level sites
Villa	V	Roman country house for wealthy people with associated productive facilities built during the Roman Republic and the Roman Empire

**Fig 2:**
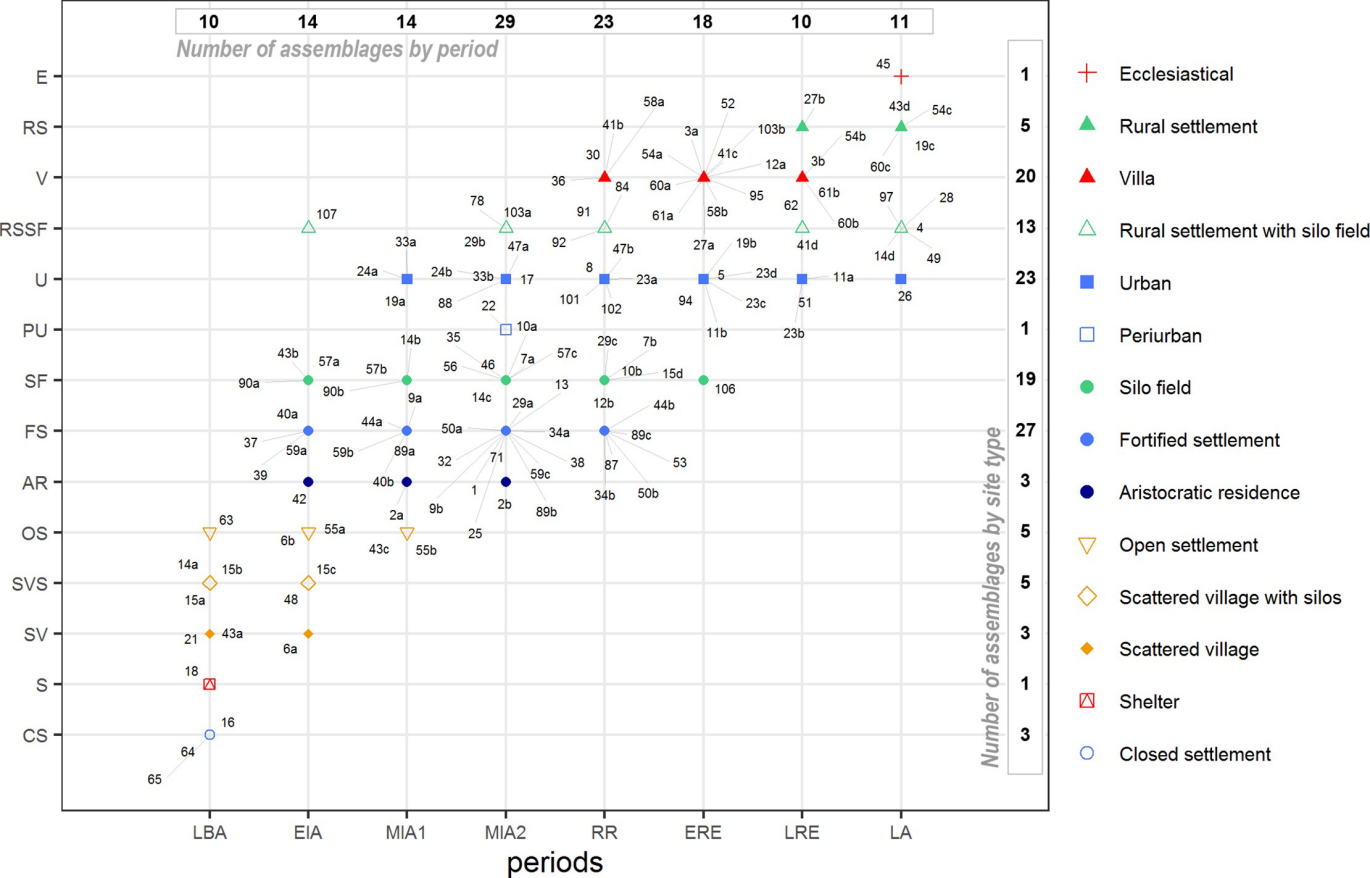
Chrono-typology seriation of the 127 studied assemblages from Late Bronze Age to Late Antiquity. (LBA: Late Bronze Age, EIA: Early Iron Age, MIA1: Middle Iron Age 1, MIA2: Middle Iron Age 2, RR: Roman Republic, ERE: Early Roman Empire, LRE: Late Roman Empire, LA: Late Antiquity). The x-axis represents the assemblages by periods and the y-axis represented the type of sites. List of assemblage numbers: 1. Esquerda; 2a. Alorda Park 2a; 2b. Alorda Park 2b; 3a. Antigons_waste dump; 3b. Antigons_*Nymphaeum*; 4. Aubert; 5. Baetulo; 6a. Barranc de Gàfols_1; 6b. Barranc de Gàfols_2; 7a. Bosc del Congost; 7b. Bosc del Congost; 8. Burriac; 9a. Ca n’Oliver 1; 9b. Ca n’Oliver 2_3; 10a. Can Bartomeu; 10b. Can Bartomeu; 11a. Can Cruzate; 11b. Can Cruzate; 12a. Can Feu; 12b. Can Feu; 13. Turó de la Rovira; 14a. Can Gambús 3; 14b. Can Gambús 3; 14c. Can Gambús 1; 14d. Can Gambús 1; 15a. Can Roqueta CRV; 15b. Can Roqueta TR; 15c. Can Roqueta DIASA; 15d. Can Roqueta TR; 16. Carretelà; 17. Ciutadella de Roses; 18. Cova Punta Farisa; 19a. Empúries; 19b. Empúries; 19c. Empúries; 21. Fonollera; 22. Gou Batlle; 23a. Ilerda; 23c. Ilerda1_Carrer Magdalena 47; 23d. Ilerda2_Carrer Bafart 46; 24a.I lla d’en Reixach 2_3; 24b. Illa d’en Reixach 4_5_6; 25. Puig Castellar; 26. Iluro_VI_VII; 27a. La Llosa; 27b. La Llosa; 28. Mallols; 29a. Mas Castellar_fortified; 29b. Mas Castellar_rural; 29c. Mas Castellar; 30. Mas Gusó; 32. Moleta del Remei 1_2; 33a. Molí d’Espígol; 33b. Molí d’Espígol; 34a. Olèrdola 1; 34b. Olèrdola 2; 35. Olius; 36. Olivet d’en Pujol; 37. La Pedrera IV-VII; 38. Penya del Moro; 39. Puig de la Misericordia; 40a. Puig de la Nau; 40b. Puig de la Nau; 41b. Sant Boi_Pl. Constitució; 41c. Sant Boi_Pl. Constitució; 41d. Sant Boi_Pl. Constitució; 42. Sant Jaume Mas d’en Serrà_sect. 1; 43a. Sant Martí d’Empúries; 43b. Sant Martí d’Empúries; 43c. Sant Martí d’Empúries; 43d. Sant Martí d’Empúries; 44a. Sant Sebastià de la Guarda; 44b. Sant Sebastià de la Guarda; 45. Santa Margarida; 46. Saus; 47a. Sigarra; 47b. Sigarra; 48. Sitges UAB; 49. Solana; 50a. St. Julià de Ramis; 50b. St. Julià de Ramis; 51. Tarraco; 52. Tolegassos; 53. Torre Cremada; 54a. Torre Llauder; 54b. Torre Llauder; 54c. Torre Llauder; 55a. Tossal Molinet I-II; 55b. Tossal Molinet III; 56. Turó del Vent 1–2; 57a. Turó Font de la Canya 0; 57b. Turó Font de la Canya 1; 57c. Turó Font de la Canya 2_3; 58a. Vilarenc; 58b. Vilarenc; 59a. Vilars 0_I; 59b. Vilars II; 59c. Vilars III_IV; 60a. Vilauba; 60b. Vilauba; 60c. Vilauba; 61a. Vil·la Vinyet; 61b. Vil·la del Vinyet; 62. Vil·la dels Ametllers; 63. Vilot de Montagut 0-III; 64. Vincamet; 65. Zafranales; 71. Coll del Moro; 78. Mas d’en Gual; 84. Rosella; 87. Puig Castellar Biosca; 88. Puig de Sant Andreu; 89a. Torre Roja; 89b. Torre Roja; 89c. Torre Roja; 90a. Mas d’en Boixos; 90b. Mas d’en Boixos; 91. Hereuet; 92. Missatges; 94. Iesso; 95. Vil·la Torre Andreu; 97. Vilans de Reig; 101. Can Mateu; 102. Can Bernat; 103a. Can Rodon; 103b. Can Rodon; 106. Can Sant Joan; 107. Mas Duran.

The **Late Bronze Age** (**LBA) (**13^th^–9^th^ c. BC**)** shows a clear duality in the settlement pattern between coastal areas (CC and NC) and the interior plain (OP). These two models probably reflect different social and economic scenarios. On the coastal areas, the continuity of the previous models of habitation from the Middle Bronze Age is well attested (i.e. open-air villages with isolated huts and storage pits, also called *silos* [63]). This is thought to reflect a family residence model, characterized by small autonomous farms scattered through the territory [8,13,25,27]. These sites were established in the vicinity of the crop fields and can contain a large number of *silos*, as is the case of Can Roqueta (15) in the CC area [64]. In contrast, in the occidental plain (OP) a proliferation of elevated sites with more stable stone structures is attested from the 10th c. BC, constituting the first closed settlements in the area (Fig 2) (e.g. Genó, Vincamet (64), Carretelà (16) and Zafranales (65); [28,65–68]). These villages are thought to represent a concentration of several families [28,68]. In the coastal areas, only one site–La Fonollera (21, NC)–includes several huts and can be considered as a small village [69,70]. Archaeobotanical data suggest that Late Bronze Age economy was mainly based on cereal production, most notably barley (*Hordeum vulgare*) and common wheat (*Triticum aestivum*) [71]. In the occidental plain (OP), agriculture probably involved ploughing, fallowing, and the use of manure as fertilizer [71]. In contrast, in the coastal areas a system of grazing and fire-based deforestation has been proposed [72,73], which would justify less stable settlements.

The absence of strong differences in the funerary record and within the settlements suggests that these societies were relatively egalitarian compared with those of later periods [74]. The end of the Bronze Age is marked by the collapse of complex long-distance exchange networks for the bronze industry (tin and copper [75,76]).

The **Early Iron Age (EIA)** (8^th^–7^th^ c. BC) is characterized by the presence of the first occurrences of iron objects, and the expansion of open settlements due to population concentration processes [27,28,77–79]. Archaeobotanical data suggest that during the EIA the farming strategies were similar to previous periods. Even so, the introduction of viticulture (mainly in eastern Catalonia) was a novelty with respect to the previous period. At this time, the agricultural rhythm based on the immediate obtaining of yields was broken. This means that the surplus agricultural production became more archaeologically visible, suggesting a change in farming strategies [67]. The palaeoenvironmental data available for the four study regions are very heterogeneous. Although both the LBA and the EIA have shown recurrent signs of anthropization of the landscape, the indicators still do not show excessive disturbances [80–82].

During the EIA important differences between the interior and the coastal areas are visible at the funerary level in different tumular traditions, and in the settlement pattern. This duality is thought to reflect two distinct economic systems [13,67,71]. The first, located in the Occidental Plain (OP), represented a centralized economy based on the large-scale production of one or several basic subsistence products. In this area, there is a low presence of imported products from the Mediterranean colonial trade [83]. The appearance of fortified sites, mostly located on elevated positions but also on the plain (e.g. Els Vilars (59), Molí d’Espígol (33), La Pedrera (37)), and ’warrior tombs’ (e.g. Necropolis of La Pedrera) have been used to suggest that coercion was an important factor for power consolidation and for exercising effective control over the territory [13,28,84]. The second socio-economic model attested in coastal areas (CN, CC, SC), is thought to have been developed from an economy of prestigious goods. In this model, small group of individuals would control both the mechanisms of exchange of colonial trade and the redistribution of prestigious import objects [6,13,25,85,86]. In both zones (interior and coastal), a process of aristocratic emergence probably occurred, in which the exchange of prestigious goods acted as an element of social cohesion [13,25,27,67,87–93]. Some elements suggest that there were also important internal conflicts. In this regard, most sites in the SC were destroyed by fire in the 7^th^ c. BC, which prevented these centers of power from fully consolidating until the 6^th^ c. BC [13,92,94,95].

The **Middle Iron Age (MIA1)** (6^th^–5^th^ c. BC) was characterized by the emergence of what has been called ’Iberian Culture’ [27,96,97]. Emerging chiefs and aristocratic groups consolidated both in the OP [28,84,98] and the coastal areas [91,99,100]. A hierarchical system of occupation was implemented, organized into several centralized political units. There was a greater diversification in settlement types: central places with fortifications (more than 3 ha in size), peripheral mid-rank settlements (3 to 1.5 ha), and small-sized scattered settlements [11,27]. At this point, coercion would be used as a means of control in a strongly territorialized context. Protection became central, as evidenced by the spread of stone architecture, the presence of defensive elements in most sites and the generalization of weapons in the tombs [27,101,102]. In this period, the use of iron is mainly attested in the manufacture of weapons, and no major changes in the production model have been identified [30,67,71].

In the **Middle Iron Age 2 (MIA2) (**4^th^–3^rd^ c. BC**),** there was significant growth of some settlements that became large urban centers (over 10 ha in size). In addition, other smaller nuclei with concentrated populations were created, and the number of dispersed farms and rural establishments generally increased, especially in NC and CC [11,25]. This settlement pattern is thought to reflect a greater integration of productive models, where agriculture experienced significant intensification, visible in the generalization of iron-made agricultural tools and the growing size of cereal storage structures [27,102–105]. In addition, fortification structures became increasingly complex (ditches, concealed entrances, towers, etc.). The development of more intensified production systems is thought to respond to both a growing internal (demographic increase) and external demand (colonial trade) [103]. The intensification processes promoted the consolidation of an administrative system managed by political entities with a larger territorial influence during the 3^rd^ c. BC. The estimated areas covered by these territories vary between 2000–2800 km^2^ for the smallest entities located in CC and NC [27], up to the 9500 km^2^ of the largest entities in the OP [106]. This new scenario was characterized by a higher degree of specialization and hierarchization of settlements, and the existence of an Iberian coinage, weight system, and writing, all of which is thought to correspond to a proto-state political structure comprising different ethnic entities [25,99,107].

The 2^nd^ Punic War (218–201 BC) and the Roman conquest of Iberia starting in 218 BC brought about profound transformations in the socio-political and economic system of the indigenous communities throughout NE Iberia. The Roman victory over Carthaginians marked the progressive incorporation of the whole area into the Roman economic and cultural system during **Republican** period **(RR) (**218–37 BC) [108–114]. During the first half of the 2^nd^ c. BC (200–150 BC) an extraction model was imposed on the allies, which were still attached to the *oppida* as territorial administrative centers. But from 150 BC onwards, there was a progressive transformation of the settlement pattern, first in the coastal areas and later in the Occidental Plain [108,115–119]. A new type of small rural sites located in the plains, without defensive systems and specialized in cereal production, spread in most areas (except in the SC [112]). The material culture and building practices were mainly of indigenous tradition, and a low degree of Roman acculturation has been proposed according to material culture and the presence of new construction techniques and structures (e.g. La Rosella (84) [120], Serrat dels Espinyers [121,122]). During the 1^st^ c. BC, newly built *villae* were erected over many of these small rural sites, thus suggesting that the territory was then fully integrated into the economic and political Roman system [108,110,123].

The **Early Roman Empire (ERE) (**37 BC–AD 250**)** is associated with the end of the conflicts in the Iberian Peninsula and the creation of the new provinces by Caesar Augustus, who founded cities and reorganized the road network [114]. In the case of the *provincia Tarraconensis*, it was a time of territorial expansion with a high degree of economic integration. The changes related to the territorial reorganization of Augustus are the culmination of a new model of settlement and occupation of the territory. In the rural areas, an important nuclearization of dispersed sites is attested, due to the spread of the *villae* system [114]. The *villae* managed large *latifundia* (Latin: *latus* ’spacious’ and *fundus* ’farm, estate’) with a production system specialized in cereals and olive trees for the *villa*, and grapes for wine production and export [15,16,124]. The large territorial extension managed by the *villae*, allowed large areas to be exploited for crop fields, to have grazing areas for the flocks, and to have a large variety of natural resources from forested areas. The *villae* also had buildings for artisanal production (e.g. large ceramic ovens) and for the storage of agricultural surplus. All this made the *villae* self-sufficient in terms of food and supply of basic products, forming self-managed production units, although within a productive system clearly devoted to the imperial market [16,111,124,125]. On the other hand, cities occupied large areas (c. 80 ha in the case of *Tarraco*), and were densely populated [114,126]. Cities also concentrated the political and administrative power in the provinces, and articulated the territory together with large consumer and redistribution centers of surpluses sourced from rural areas [127–129]. The larger regional administrative units of the empire (compared to the pre-Roman periods) and the large road network were key elements in facilitating long distance trade and the circulation of products, livestock and people. The high degree of connectivity allowed this system to maintain a new large-scale production system, balancing the growing supply and demand [18,19].

During the **Late Roman Empire (LRE)** (AD 250–472), the pattern of rural settlements in the *provincia Tarraconensis* underwent important changes [129–131]. After the crisis of the 3^rd^ c. AD, wine production in the *Tarraconensis* significantly decreased and some *villae* were partially or totally abandoned, or their facilities reduced [59,60,114,132]. Subsequently, some remaining *villae* experienced refurbishments and the monumentalization of some of their buildings during the 4^th^ c. and the first decades of the 5^th^ c. AD [129]. There were important changes in the relationships between the urban and rural worlds, which appear less interconnected and integrated compared with the Imperial period. These territorial changes are thought to reflect a progressive concentration of the land in the hands of fewer (and richer) owners [114]. From circa AD 450, small peasant houses are attested across the *provincia*, together with a progressive concentration of rural population in small rural hamlets and a significant reduction in the number of sites [129]. This period is often referred to as one of economic, demographic and urban recession, as evidenced by the decrease, and abandonment of some buildings, as attested in cities such as *Ilerda* (23, in the OP), *Tarraco* and *Iluro* (51 and 26, in the CC) among others [133,134].

During **Late Antiquity (LA) (**AD 470–572), Visigoth groups from Aquitaine gained control of the area. Between 470 and 475 AD, Euric conquered Roman Hispania and occupied *Tarraconensis* [135]. Despite the resistance of the local nobles, Visigoths conquered the whole territory very quickly [136]. In 507 AD, the kingdom of Toulouse disappeared. The Visigothic court initially moved from Narbonne to Barcelona (between 531 and 572 AD), although it later moved the capital to Toledo. From the second half of the 5^th^ c. AD, and throughout the 6^th^ c. AD, the settlement pattern was reconfigured with a certain continuity of the pre-existing sites, although very transformed [59,114,129,137–146]. There was a gradual disappearance of the *villae* and archaeological evidence indicates important changes in their functionality and in the ways of rural life [132,142,147,148]. The drastic reduction of imports and the size of production facilities (e.g. wine and olive presses, warehouses, silos) suggest a decrease of production, adapted to the demand of more local/regional markets [149]. Some authors highlight the decline of long-distance Mediterranean trade as a result of the Byzantine conquest of North Africa, Italy, Sicily, Balearic Islands and South-eastern Hispania [150]. The monumental structures of the *villae* (e.g. baths, spaces of representation) were abandoned or re-used for productive activities, and building materials were extensively recycled to erect new and smaller habitation and productive units. New rural settlements also spread in the territory in the form of small agricultural villages with huts built with perishable materials (wood and other plant materials, [142,144,148]), which are thought to be inhabited by small family groups [151]. The period has been described as a time of self-reliance as a basic strategy, and of technological and architectural modesty. Although at this time the arrival of imports from other places of the Mediterranean is still attested in the *Tarraconensis*, this was mainly limited to coastal areas and cities located at main communication axes. These transformations are thought to reflect an increasingly autarkic and independent system of rural areas with respect to cities [152].

## 4. Materials and methods

### 4.1 Archaeological sites and zooarchaeological assemblages

Fig 1A shows the location of the 101 archaeological sites considered in this study, among which 28 have NISP data (number of identified specimens, green points), 23 have biometry data (blue points), and 34 have both data (black points). The complete dataset and related references are detailed in supplementary materials S1 in S1 File. A total of 16 settlements (red points) were excluded from the analyses due to contextualization problems or to a low number of remains (< 110 NISP of main domesticates). As stated above, the studied area was divided into four zones (Fig 1A). To guarantee greater precision in the location of the sites and the study areas, all data were integrated into an open access geographic information system (QGIS). The criteria for defining the different areas of study considered their geographical, ecological and material culture coherence. The chrono-cultural framework (Table 1) was chosen to follow a broadly accepted periodisation [27,28,62,149,153]. The time-span was divided in eight different periods covering *circa* two centuries each, except for the Late Bronze Age (LBA, three centuries) and Late Antiquity (LA, three centuries). These time intervals allowed us to have a sufficient number of sites and data for each period.

In order to look for correlations based on their characteristics, the sites were classified into 14 categories based on their typology, functionality and surface extension following previous works on the topic (Table 2 and Fig 2). Each site was given a number, and different phases / periods of occupation were distinguished with different letters of the alphabet (e.g. 1a; 1b; 1c, etc. See S1 and S2 in supplementary materials in S1 File). Contexts with a broad chronological attribution (e.g. ’Iron Age’), or including residual or intrusive materials were also excluded. In total, 127 contexts have been studied.

The corpus of zooarchaeological data for this meta-analysis– 82,774 identified remains (see S1 in supplementary materials in S1 File) and 2,354 unique measurements (see S4 in supplementary materials in S4 File)–originates both from published works and unpublished reports (grey literature) written more than 10 years ago (2009 or before). New zooarchaeological analyses were also undertaken during the ERC-StG ZooMWest project (ERC-StG 716298) in order to increase the dataset for the periods with less available data (e.g. Late Antiquity). All the assemblages originate only from deposits found in habitat contexts, or non-specialized waste deposits, to reflect specifically the daily use of animal husbandry. Therefore, assemblages from ritual contexts (i.e. sanctuaries, necropolises, temples), animal bone groups (ABGs, special faunal depositions), military settlements, and sites with a seasonal occupation were excluded. In addition, we considered the contextual information available (e.g. taphonomical characteristics) and, as stated above, only the assemblages numbering more than 110 identified main domesticates specimens (cattle, pig, sheep and goat) were included in the analyses.

### 4.2 Quantification units and statistical analyses

The meta-analysis focused on the main domesticates because they constituted, by and large, the main meat source for all analyzed areas and periods according to zooarchaeological data [53]. The units of comparison used were relative frequencies calculated using the number of identified specimens (NISP) of the main domesticates–cattle, sheep, goat, and pig–, and log ratios calculated from biometric data. In order to explore the relations between site typology, site location, and changes in animal husbandry (NISP and biometry) over time, we performed various data visualizations and statistical analyses based on these variables: type of sites, geographical areas, and chronological periods.

NISP was selected as it is generally present in the reports and is more stable than other quantification units such as MNI or MNE [154,155]. The kill-of-patterns provide key information to characterize animal husbandry practices and assess changes through time. Unfortunately, the absence of a standardized way of recording and displaying the information made it impossible to compare and systematize the mortality profiles between different works.

Correspondence analyses (CA) were used to explore i) correlation between site’s livestock frequencies, site’s types and geographical areas; and ii) similarities between contemporaneous sites (intra-period comparisons). A hierarchical clustering analysis (HCA) based on these results allowed grouping of sites with similar animal consumption profiles. Significance in the observed differences between sites were assessed using Chi-square tests with the ‘chisq.test’ R native function. The CA algorithm comes from the R package ‘FactoMineR’ [156]. To avoid size effects, measurement of differences between sites were performed on the normalized values of cattle, pig and sheep/goat (main domesticates) with the ‘dist’ function of the basic package of R [157]. The HCA agglomeration method was complete (complete-linkage clustering) and performed with the ‘hclust’ R native function. On these figures, CA and HCA, site types and geographical areas are displayed using different shapes and colors in the symbols, but these variables did not have any effect on the clustering statistics.

Biometric analyses considered osteological size changes of postcranial elements from the main domestic species (sheep/goat, cattle and pig). Biometric data from actual domestic and wild species were also included in the graphs for comparative purposes, particularly to help assess the potential presence of wild individuals (or hybrids) in the archaeological samples. In order to establish the differences and limits between domestic and wild individuals, archaeological wild boars from different sites and periods and biometric data of actual *Capra pyrenaica hispanica* and *Capra pyrenaica pyrenaica* (extinct in January 2000) [158] were included in the graphs (Figs 7 and 8). Modern specimens of *Capra pyrenaica* were used as a comparative reference for zooarchaeological individuals. Consequently, they were both included in the "actual" category. The purpose of including wild goats was to identify if the observed size increase in the domestic sheep/goats was due to the presence of wild animals in the dataset. In addition, data from individuals from two actual local breeds of sheep and goats–Xisqueta breed sheep and Catalan goat breed–were also included as reference measurements of present-day domestic individuals adapted to the ecology of the areas studied (Figs 7 and 8).

Changes in animal size were assessed using size-index scaled Log standard index values (LSI) [159]. Widely available standards were used for cattle [160], sheep and goats [161], and pigs [162]. The analysis follows the methodology described in Table 1 of [51]. However, in this case the SLC (smallest length of the collum scapulae [163]) was also used as a last option when no other width measurement was available. Differences between osteometrical distributions were assessed using Mann-U tests, with the R native function ‘wilcox.test’ [164], at different levels for each species (cattle, pig, sheep/goat) through periods and between areas.

## 5. Results

### 5.1 Site patterns in livestock ratios through time

During the **Late Bronze Age** (LBA, 13^th^–9^th^ c. BC; Fig 3.1 top), the sites located in the Occidental Plain (OP) display a high frequency of sheep and goat remains (between 57 and 85% of the main domesticates). This is attested in different site types: Open settlements (OS), Closed settlements (CS) and shelters (S). In the Central Coast (CC) and the North Coast (NC) sheep and goats are also predominant (67,8%) but with higher proportions of pig (14,6%) and cattle (17,6%) (Fig 5). Although some sites have profiles that stand from the other sites in their area–e.g. Zafranales (65) from the OP, or Can Gambús 3 (14a) and Can Roqueta CRV (15a) from the CC–, the general trend is a high correlation between area and percentages of species consumed (as shown in Fig 4). The NISP results suggest that in this period the composition of the livestock frequencies are closely related to the location of the site (zone) and not to the settlement type.

**Fig 3:**
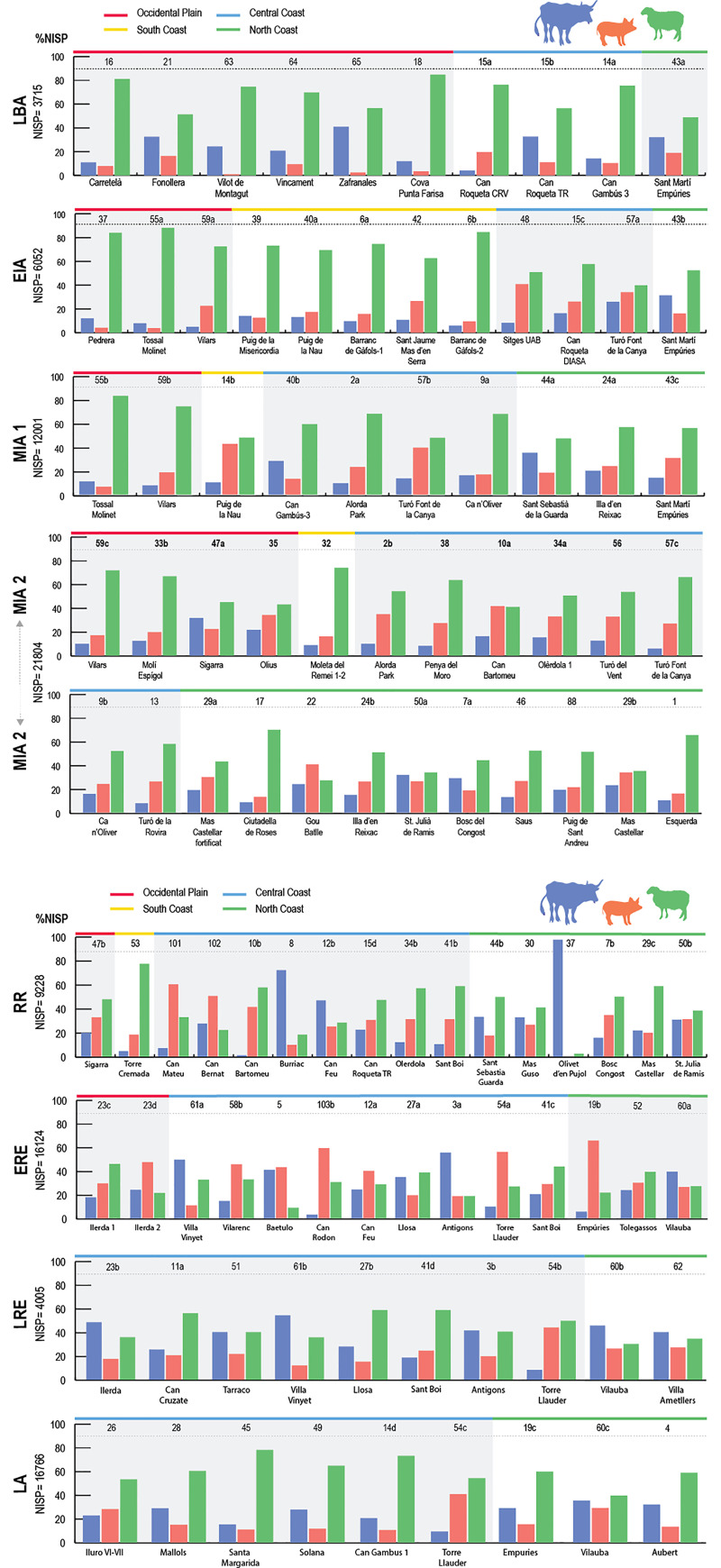
**3.1. NISP percentages histograms of main domesticates** (cattle, pig and sheep/goat) by site and periods (LBA: Late Bronze Age, EIA: Early Iron Age, MIA1: Middle Iron Age 1, MIA2: Middle Iron Age 2). Numbers of sites refer to Fig 2. **3.2 NISP percentages histograms of main domesticates** (cattle, pig and sheep/goat) grouped by sites and ordered by periods (RR: Roman Republic, ERE: Early Roman Empire, LRE: Late Roman Empire, LA: Late Antiquity). Numbers of sites refer to Fig 2.

**Fig 4:**
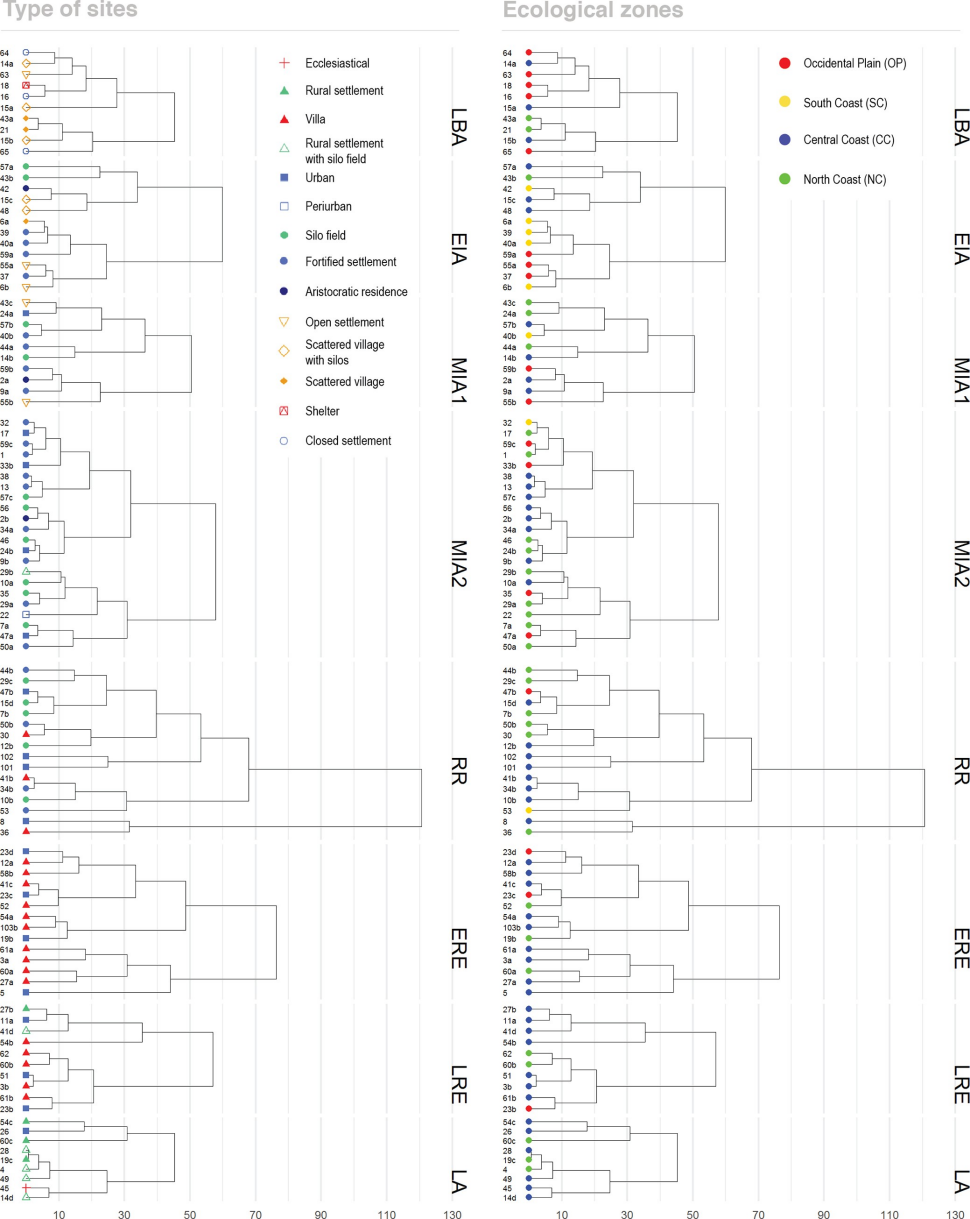
Hierarchical clustering analysis (HCA) of sites’ NISP profiles ordered by periods. (LBA: Late Bronze Age, EIA: Early Iron Age, MIA1: Middle Iron Age 1, MIA2: Middle Iron Age 2, RR: Roman Republic, ERE: Early Roman Empire, LRE: Late Roman Empire, LA: Late Antiquity). Numbers of sites refer to Fig 2. Dendrograms are identical expect except for site labelling: HCA left aligned shows symbols of sites’ types (see Fig 2). HCA right aligned shows symbols of sites’ geographical zones (see Fig 1).

**Fig 5:**
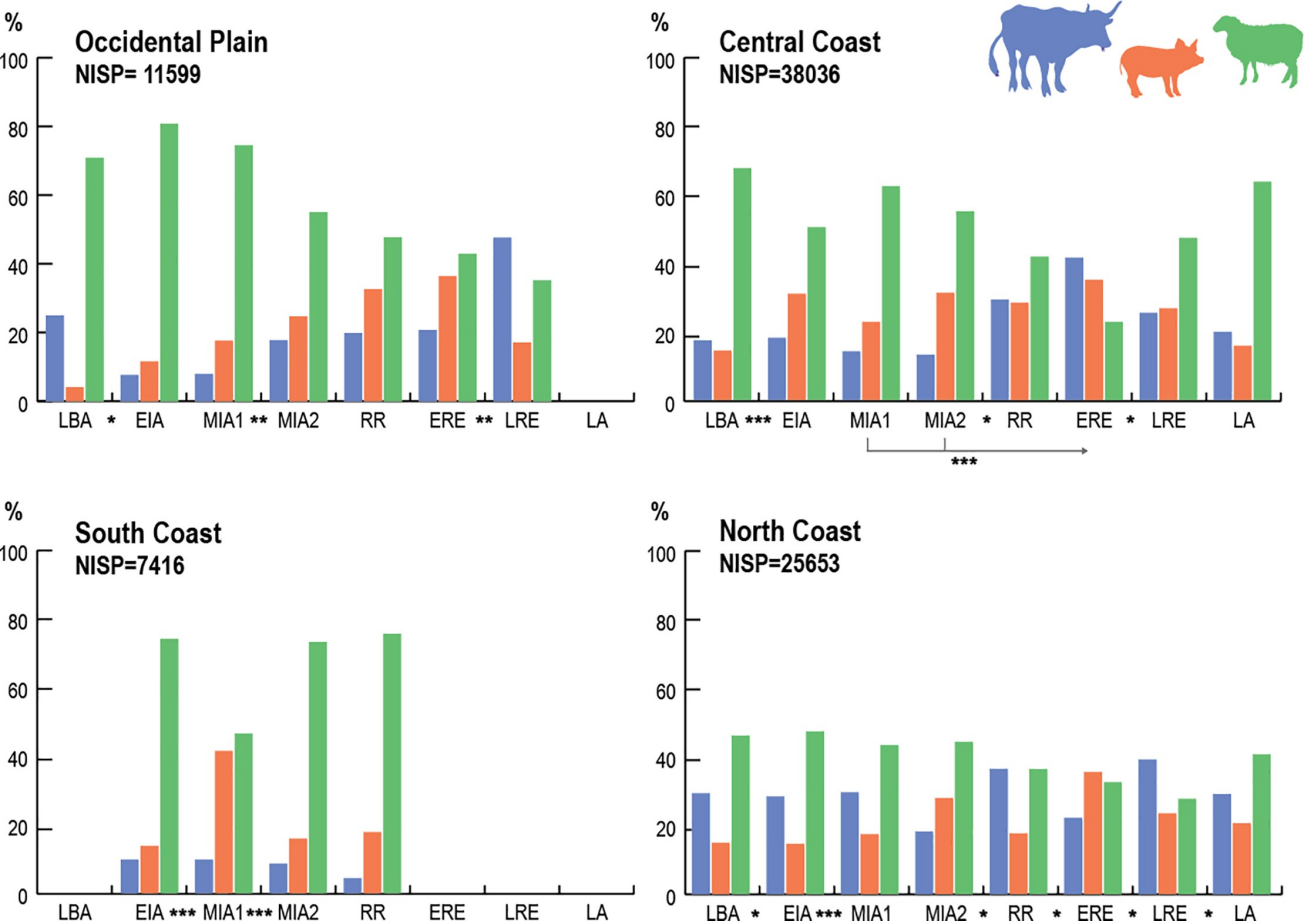
NISP percents of main domesticates (cattle, pig and sheep/goat) grouped by zones and ordered by periods. (LBA: Late Bronze Age, EIA: Early Iron Age, MIA1: Middle Iron Age 1, MIA2: Middle Iron Age 2, RR: Roman Republic, ERE: Early Roman Empire, LRE: Late Roman Empire, LA: Late Antiquity). Chi-square results showing significant changes between periods are displayed with their *p-value*: ***: highly significant (< 0.01); **: significant (< 0.05); * less significant (< 0.1).

During the **Early Iron Age** (EIA, 8^th^–7^th^ c. BC; Fig 3.1) the two fortified stone-built settlements (FS) with NISP data in the OP, La Pedrera (37) and Vilars 0-I (59a), display lower frequencies of cattle remains (12–4,9%) and higher frequencies of sheep and goats (84–72,6%) compared with the LBA sites in the same region. The site of Vilars, the first in which a complex fortification (other than a closing wall) is attested, had a higher frequency of pig remains (22,5%) compared with the two other OP sites. This NISP profile, in which sheep and goats are the most common species, followed by pigs, and then cattle, is also attested at all the fortified stone-built settlements with zooarchaeological data located in the South Coast (SC) as Puig de la Misericordia (39) and Puig de la Nau (40a). The *silos* (SF) of Sant Martí d’Empúries (43b), in the NC, display a notable continuity with the NISP profiles attested in LBA assemblages of this area. Exploratory analysis showed a significant clustering according to settlement type and livestock ratios, in a moment when there was a greater diversification of the typology of sites (Fig 4). The six different types of settlements with NISP data are associated with different livestock profiles (Fig 4). The scattered villages with *silos*–e.g. Can Roqueta DIASA (15c) and Sitges UAB (48)–and the Aristocratic Residence–Sant Jaume Mas d’en Serrà _sect. 1 (42)–are strongly correlated with high frequencies of pigs (26–41%) (Fig 3.1). These sites have also in common the important concentrations of colonial imported products from the Mediterranean. Another subgroup within the previous one (see Fig 4), are the silo fields–Turó Font de la Canya 0 (57a) and Sant Martí d’Empúries (43b)–, which are strongly correlated with cattle (26–33,3%) (Fig 3.1). Another big group aggregates the open settlements–e.g. Tossal del Molinet I-II (55a) and Barranc de Gàfols 2 (6b)–which have a great continuity with the previous period, with higher proportions of sheep/goats (88,2–83,3%) (Fig 3.1). Finally, the fortified settlements (with more complex urbanism) have pig frequencies between 10–20%–e.g. Puig de la Misericordia (39), Puig de la Nau (40a), and Vilars 0-I (59a).

In the **Middle Iron Age 1** (MIA 1, 6^th^-–5^th^ c. BC), the two sites–Tossal del Molinet (55b) and Vilars 59b –available for the OP continued with the same NISP distribution they had in EIA levels (Fig 3.1). Puig de la Nau (40b), the only site with NISP data in the SC, also has a profile where sheep and goats predominate (48%) followed by pigs (42%) and then cattle (10%). This ’staircase’ pattern is also attested at most stone-built sites located in the OP and CC–fortified settlements like Ca n’Oliver (9a) and Vilars II (59b), and aristocratic residences like Alorda Park 2a (2a)–, with the exception of Can Gambús 3 (14b) (Fig 3.1). In the NC, the fortified settlement of St. Sebastià de la Guarda (44a) displays the ’U-shaped’ pattern characteristic of the LBA assemblages in the area, with abundant cattle and sheep/goats (49,6%) and few pigs (17,4%), while the other two fortified settlements have ’staircase’ pattern NISP distributions, in which sheep/goats are followed by pigs and then cattle. We recall here that this was the time when a real hierarchy between settlements is attested in the region. Unlike the previous period, no correlation between the livestock ratios and the ecological zones or site typology could be identified (Fig 4). At the same time, the differences in livestock ratios between sites are less apparent, thus suggesting a slight process of homogenization of the zooarchaeological records.

During the **Middle Iron Age 2** (MIA 2, 4^th^–3^rd^ c. BC) two sites in the east of the OP–Olius (35) and Sigarra (47a)–display cattle frequencies over 20%, in contrast with the other two sites available further west in this area–Molí d’Espígol (33b), Vilars III-IV (59c)–, which display the ’staircase’ pattern (Fig 3.1). This NISP ’staircase’ pattern is also found in the two sites available in the SC–Penya del Moro (38), Moleta del Remei (32)–and across most sites in the Central Coast, no matter their extent or characteristics. Only the assemblages coming from some *silos* in Can Bartomeu (10a) (Fig 3.1, penultimate row) have NISP profiles that slightly differ from the rest. In the NC, the faunal assemblages have a higher degree of diversity. The peri-urban houses of Gou Batlle (22) stand because of the predominance of pig remains (44,3%). Several sites display the ’staircase’ pattern while two sites–the fortified settlement of Sant Julià de Ramis (50a) and the *silos* of Bosc del Congost (7a)–had the ’U-shaped’ pattern commonly found during the LBA. In this area, cattle frequencies are generally higher (over 20%) compared with the sites located in the CC and the SC. The NISP profiles of first-order cities–like Molí d’Espígol (33b), Illa d’En Reixach (24b) or Puig de Sant Andreu (88)–are not correlated one to another, but are similar to silo fields and other fortified settlements (Fig 4). From the end of the 4^th^ c. BC there was a greater diversification and hierarchization between sites, and this contrasts with the homogeneity and the predominance of the ’staircase’ pattern at most sites (Fig 3.1 final two rows). The short length of the dendrogram’s branches (Fig 4) shows that the differences between sites are very small, and suggests a process of homogeneity or generalization of similar dietary practices.

The **Roman Republican** period (RR, 218–27 BC) displays a different and much more diversified picture (Fig 3.2, top). Many changes took place between the middle of the 2^nd^ c. BC and the 1^st^ c. BC, as it coincided with the Roman conquest (RR) and the progressive disappearance of the Iberian system of *oppida*, that was replaced by the Roman system of the *villae* (V). The long length of the dendrogram’s branches, the widest of all the periods, shows that the differences in livestock patterns between sites were very high (Fig 4). Several sites from the OP, the SC and the CC have the ’staircase’ pattern characteristic of the Middle Iron Age. They include newly built sites–e.g. Torre Cremada (53), Sant Boi_Pl. Constitució (41b)–and others previously inhabited–e.g. Sigarra (47b), Olèrdola (34b), Can Roqueta TR (15d). In contrast, cattle remains predominate on two sites in the CC with a previous occupation–Can Feu (12b) (46,7%) and Burriac (8) (72%)–, and pig is the most represented species at two newly built sites in the same area–Can Bernat (102) (50,5%) and Can Mateu (101) (60,2%). In the NC, cattle frequencies are generally over 20%, and some sites display the ’U-shape’ pattern in which caprines and cattle constitute the majority of faunal remains (Fig 3.2, top). The diversity in the NISP profiles shows an important heterogeneity between geographical areas and types of sites (Fig 4).

The assemblages dated from the **Early Roman Empire** (ERE, 27 BC–AD 250; Fig 3.2) also have a notable diversity. There is a hierarchical and differentiated NISP distribution according to site type, with an important dichotomy between the *villae* and urban sites.

A first large group is attested with three related subgroups within Fig 4. A first subgroup, formed by two assemblages–the city of *Ilerda* 1_Carrer Magdalena 47 (23c) in the OP, Sant Boi-Plaça Constitució (41c) in the CC and the *villa* of Tolegassos (52) in the NC–have NISP profiles similar to the ’staircase’ pattern of the Iron Age, although with lower values of caprines (about 40%). A second one groups the *villae* of Vilarenc (58b) and Can Feu (12a) in the CC, and another set of the city of *Ilerda* 2_Carrer Bafart 46 (23d). This second subgroup is characterized by a higher percentage of pigs (about 40%) and lower frequencies of caprines (about 30%) compared with the first subgroup. The third subgroup counts the richer *villae* of Can Rodon (103b) and Torre Llauder (54a) in CC, and the city of Empúries (19b) in NC. This subgroup is also characterized by high percentages of pigs (60–70%) and very low percentages of cattle (below 10%).

A second large group is formed, firstly, by the *villae* of Vinyet (61a), Antigons waste dump (3a) and La Llosa (27a) in the CC, and Vilauba (60a) in NC. It is composed of two subgroups correlated with high percentages of cattle (between 40–60%). Finally, the city of Baetulo (5), the main urban site of the area, has a different profile characterized by lower percentages of sheep/goats (c. 10%), and a balance between pigs and cattle (c. 40%).

Although there seems to be quite a lot of diversity, there are some general patterns (Fig 3.2). The tendencies observed on NISP frequencies show that some *villae*–e.g. Can Rodon (103b), Vilarenc (58b), Can Feu (12a)–, cities–Empúries (19b)–, and the rich *domus* of Torre Llauder (54b) (also located in an urban context)–have high pig frequencies (c. 60–70%). The cities of Baetulo (5) and *Ilerda* (23c, 23d) also present high values of pig remains, although in smaller percentages (36–46%). Other *villae* have lower percentages of pig (10–28%) and are strongly correlated with cattle–e.g. La Llosa (27a), Vilauba (60a), Vinyet (61a), Antigons_waste dump (3a), and Tolegassos (52).

The picture changed again during **Late Roman period** (LRE, AD 250–472). Three groups are observed according to livestock frequencies during this period. A first subgroup presents very homogeneous profiles, with six out of the ten assemblages displaying a ’U-shape’ pattern in which cattle remains predominate over caprines or have similar frequencies (Fig 3.2, center-bottom). This is the case of the urban sites of Ilerda (23b) in OP and Tarraco (51) in CC, as well as the *villae* of Antigons_*Nymphaeum* (3b), Vinyet (61b), Vilauba (60b) and Ametllers (62). Other two *villae*–Can Cruzate (11a) and La Llosa (27b)–also have a ’U-shape’ profile but with a greater predominance of caprine remains (55–57,7%). The rural settlement of Sant Boi (41c) displays the ’staircase’ profile. Finally, the *villa* of Torre Llauder (54b) preserved a high frequency of pig remains (over 40% NISP), although surpassed by caprines, which significantly differentiates its profile from the rest of the sites. For the first time, some *villae* and urban contexts had profiles where sheep/goats are clearly the most frequent species (between 40–60%), and there was a drastic decline of pigs in all of them.

The assemblages available from **Late Antiquity** (LA, AD 472–711) are all located in the CC and the NC (Fig 3.2, bottom). New rural settlements appear at this time, which coexist with the last Roman cities and *villae*. These newly created settlements have a totally different architecture and are characterized by NISP profiles with a higher predominance of sheep/goats (40–70%) followed by cattle (14–27%). Most sites–six out of nine, sites 28 to 14d in Fig 4– form a subgroup with very homogeneous NISP profiles, characterized by the predominance of caprine remains (57,9–70,2%), followed by cattle (10,1–27,7%) and then pigs (10,1–14,4%) (Fig 4). These sites have livestock ratios similar to those attested in LBA and EIA assemblages. On the other hand, the ancient *villae* of Torre Llauder (54c) in CC and Vilauba (60c) in NC display similar profiles compared to their Late Roman levels, but with an increase of caprine remains (38–52%) in both cases. In this sense, these *villae* and the city of Iluro VI-VII (26) display a pattern of consumption similar to Roman times, with high percentages of pig remains (27,1%) (Fig 3.2, bottom).

### 5.2 Regional patterns in livestock ratios through time

Meta-analysis of NISP data for taxon abundance of the main domesticates (S3 File) suggests that livestock representation varied significantly between regions and time periods. Fig 5 displays the trends obtained from aggregating the different sites available in each area and period.

**Fig 6:**
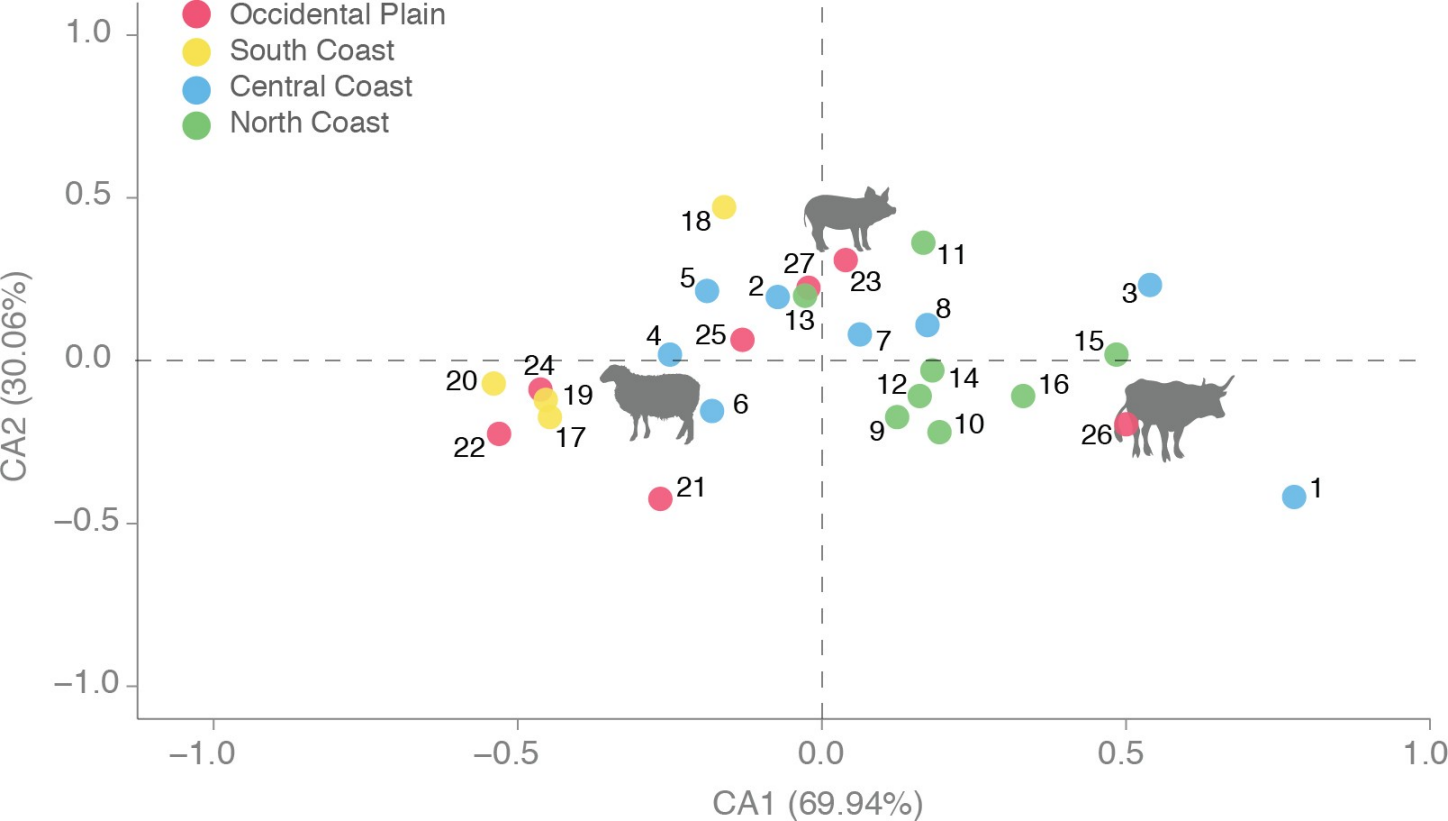
Correspondence Analysis (CA) on main domesticates NISP (cattle, pig and sheep/goat) grouped by periods and zones (i.e. statistical individuals). Symbol colors refer to the geographical zones (see Fig 1): Occidental Plain (red points), South Coast (yellow points), Central Coast (blue points), North Coast (green points). Main domestic species (i.e. statistical variables) are represented by their animal shape. Numbers refer to zones and periods: 1. CC-LBA; 2.CC-EIA; 3.CC-ERE; 4.CC-MIA1; 5.CC-MIA2; 6.CC-LA; 7.CC-ERE; 8.CC-RR; 9.NC-LBA; 10.NC-EIA; 11.NC-ERE; 12.NC-MIA1; 13.NC-MIA2; 14.NC-LA; 15.NC-LRE; 16.NC-RR; 17.SC-EIA; 18.SC-MIA1; 19.SC-MIA2; 20.SC-RR; 21.OP-LBA; 22.OP-EIA; 23.OP-ERE; 24.OP-MIA1; 25.OP-MIA2; 26.OP-LRE; 27. PO-RR.

Sheep/goats generally predominate in all areas and periods, but significant differences across time and space exist. The sites located in the **Occidental Plain (OP)** and **South Coast (SC)** are strongly correlated with sheep and goats compared with the other areas (Fig 6). In the OP, changes in the livestock ratios occurred more progressively compared to coastal zones. Correspondence analysis (Fig 6) indicates that the **Central Coast (CC)** mostly correlates with pig and sheep and goat remains. The most pronounced diachronic changes occur in this area. Conversely, the **North Coast (NC)** displays the most stable trend through time (Fig 5). This last area shows a strong correlation with cattle, except for the Middle Iron Age (MIA) and Early Roman Empire (ERE), when it had a higher percentage of pigs, like the other areas.

This inter-regional comparison (Fig 6) showed that each area kept these peculiarities during most periods, showing significant differences between them. Only during MIA2 did these differences seem to disappear or soften (see also Fig 4). In Roman times, and despite local differences, all areas display a greater proportion of pigs and a drastic decrease in sheep/goat frequencies.

### 5.3 Variables of homogeneity/diversity in livestock ratios

Exploratory and descriptive statistics (Fig 4) on NISP over these periods show two main husbandry systems (Iron Age and Roman) framed by transitional stages (LBA, RR, LA). The whole Iron Age showed a strong correlation with sheep and goats, while all areas are strongly correlated with pigs and cattle during the Roman period (Fig 5). At the site level, the initial diversity between sites observed in LBA and EIA assemblages was progressively reduced during the MIA (Figs 3 and 6). In Roman times, there was a higher diversity between sites, coherent with the greater diversity and specialization of settlement types (i.e. urban contexts vs. *villae*). Interestingly, the transitional period between these two different socio-political systems (RR), marked by the Roman conquest, displays the highest degree of diversity between zones and sites.

### 5.4 Changes in animal size through time

**Fig 7:**
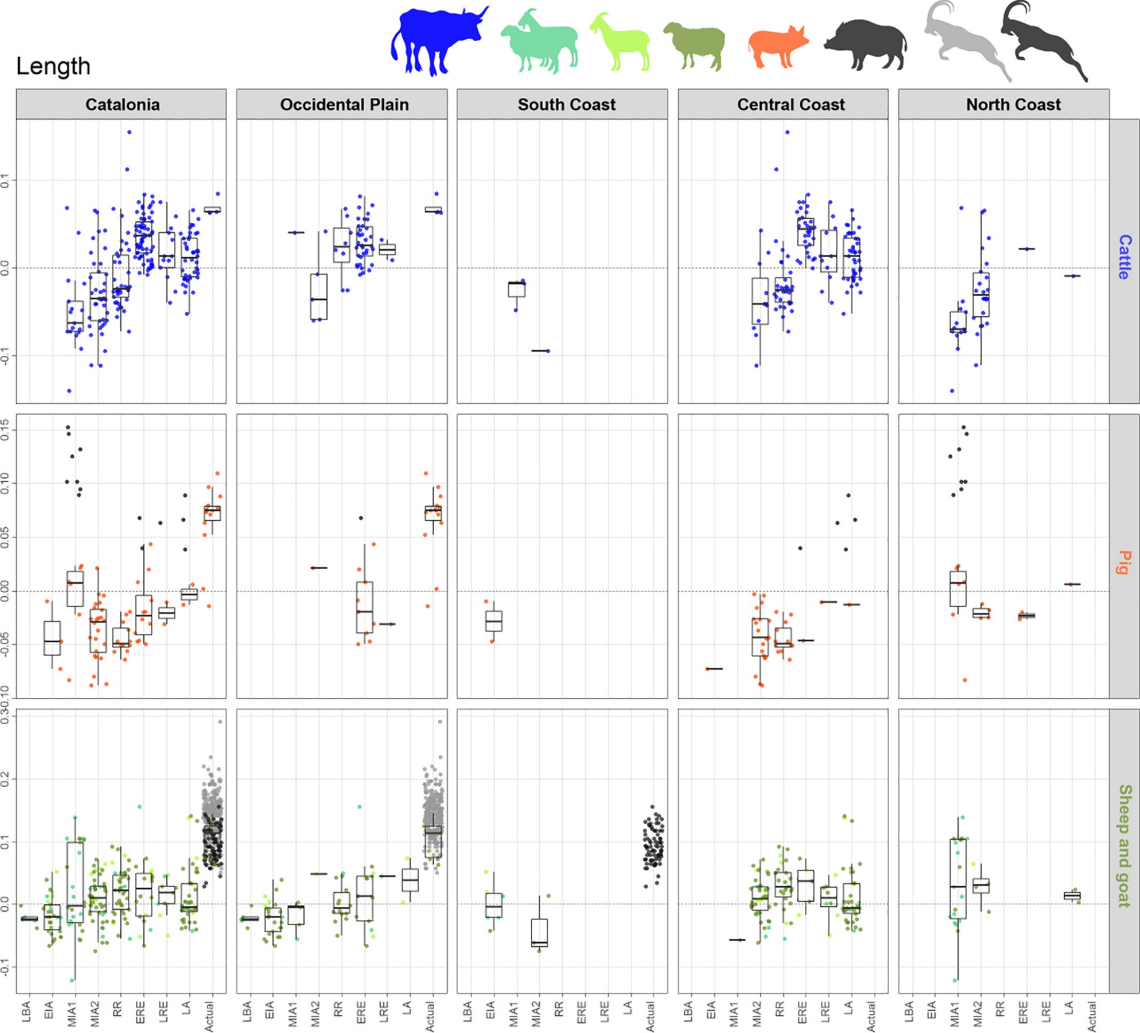
Length LSI values distribution diagram of cattle, pig and sheep/goat bones, by species (in rows) and geographical areas (in columns). Sheep and goats were considered together (see text for details). For each cell of the diagram, LSI values are displayed on the y-axis and chronological periods on the x-axis: LBA: Late Bronze Age, EIA: Early Iron Age, MIA1: Middle Iron Age 1, MIA2: Middle Iron Age 2, RR: Roman Republic, ERE: Early Roman Empire, LRE: Late Roman Empire, LA: Late Antiquity. Box plots quartiles measures only represent statistics on main domesticates, i.e. wild individuals values are displayed but excluded from statistics. Wild individuals: wild boar (black points), actual *Capra pyrenaica hispanica* (black points), modern *Capra pyrenaica pyrenaica* (grey points) [15]; actual Xisqueta breed sheep and Catalan goat breed.

**Fig 8:**
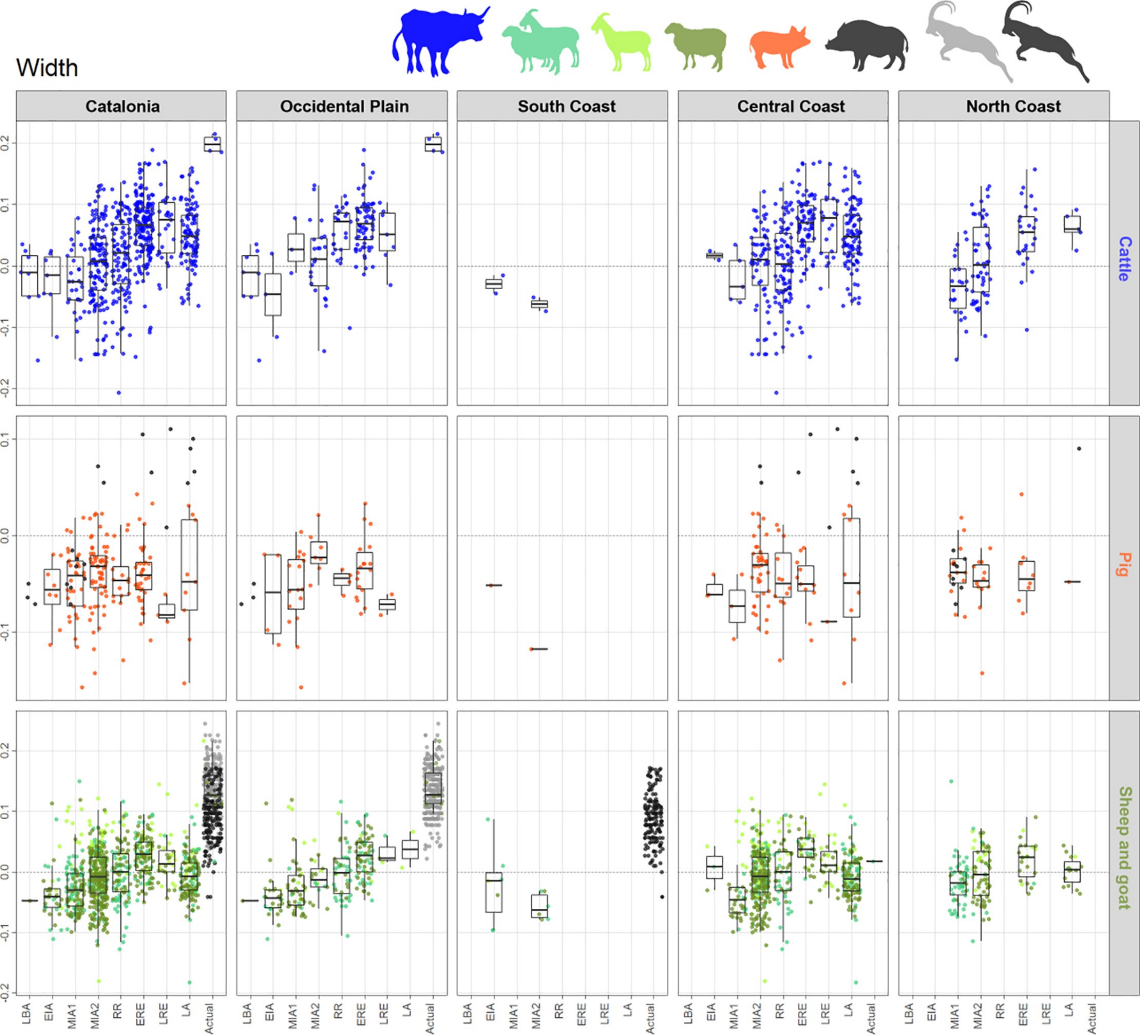
Width LSI values distribution diagram of cattle, pig and sheep/goat bones, by species (in rows) and geographical areas (in columns). Sheep and goats are considered together. For each cell of the diagram, LSI values are displayed on the y-axis and chronological periods on the x-axis: LBA: Late Bronze Age, EIA: Early Iron Age, MIA1: Middle Iron Age 1, MIA2: Middle Iron Age 2, RR: Roman Republic, ERE: Early Roman Empire, LRE: Late Roman Empire, LA: Late Antiquity. Boxplots quartiles measures only represent statistics on main domestic, i.e. wild individuals values are displayed but excluded from statistics. Wild individuals: wild boar (black points), actual *Capra pyrenaica hispanica* (black points), modern *Capra pyrenaica pyrenaica* (grey points) [15]; actual Xisqueta breed sheep and Catalan goat breed.

**Fig 9:**
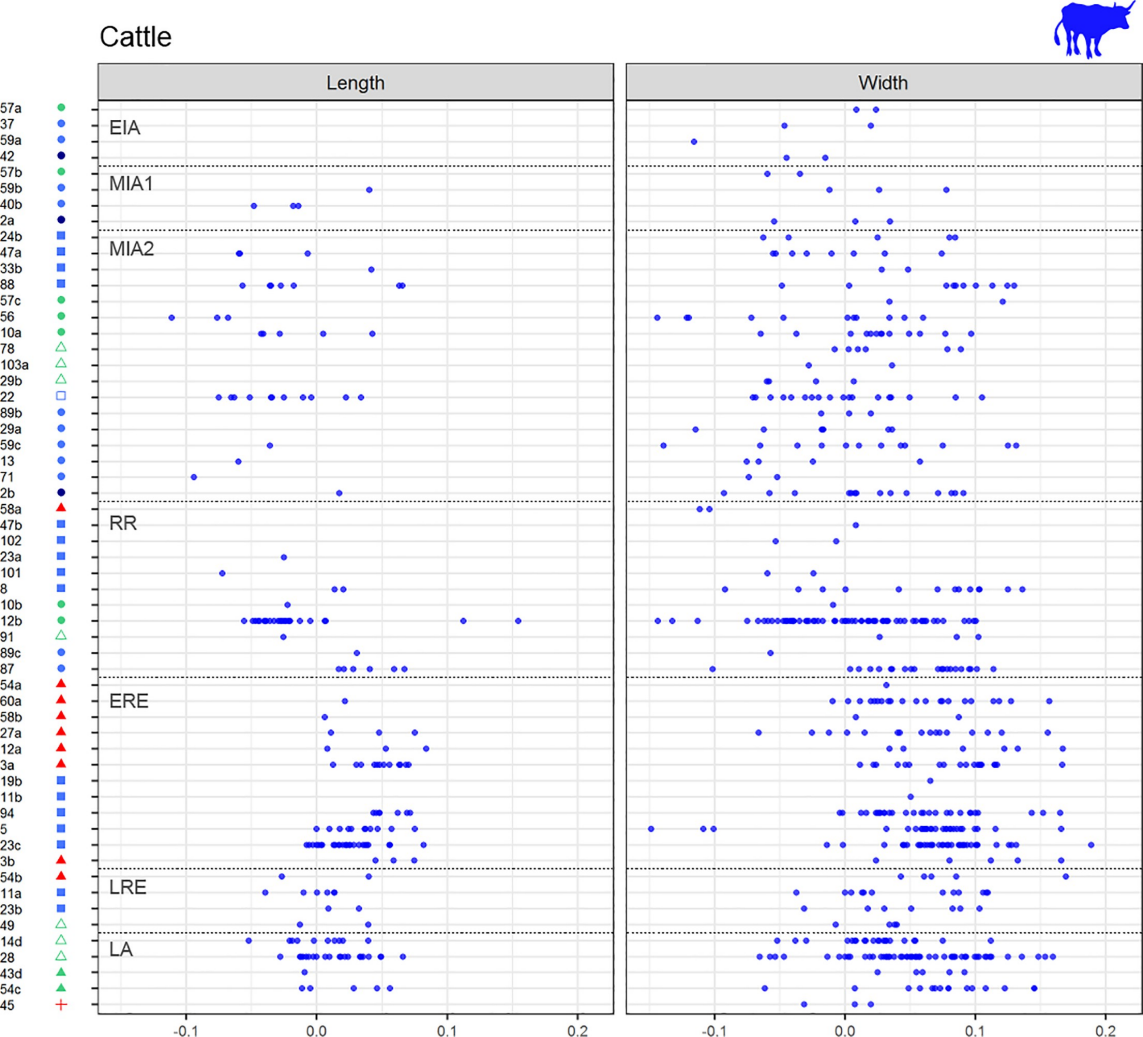
Cattle LSI length and width values by assemblages ordered by periods. (from top to bottom, EIA: Early Iron Age, MIA1: Middle Iron Age 1, MIA2: Middle Iron Age 2, RR: Roman Republic, ERE: Early Roman Empire, LRE: Late Roman Empire, LA: Late Antiquity). Left column shows length LSI values, right column shows width LSI values. Assemblages’ symbols refer to their type (see Fig 2).

**Fig 10:**
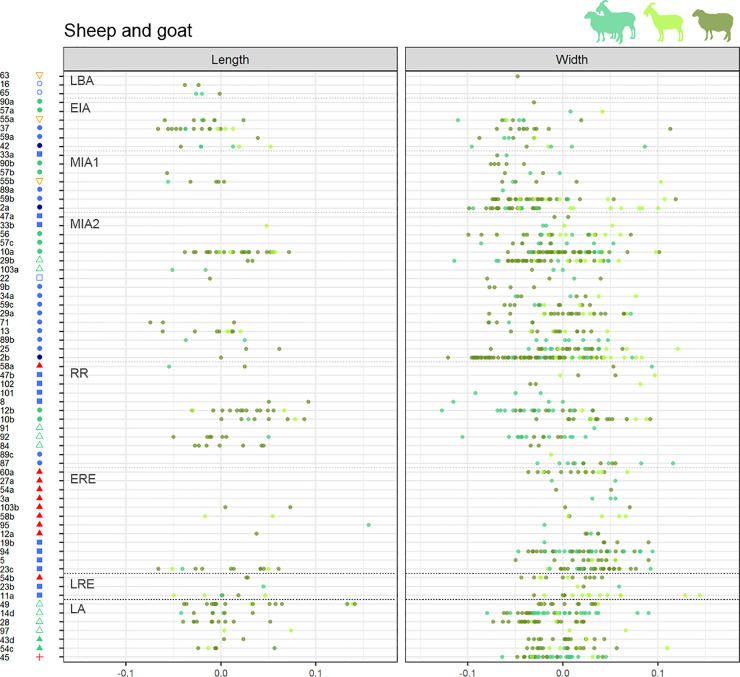
Sheep and goats LSI length and width values by assemblages ordered by periods. (from top to bottom, LBA: Late Bronze Age, EIA: Early Iron Age, MIA1: Middle Iron Age 1, MIA2: Middle Iron Age 2, RR: Roman Republic, ERE: Early Roman Empire, LRE: Late Roman Empire, LA: Late Antiquity). Left column shows length LSI values, right column shows width LSI values. Assemblages’ symbols refer to their type (see Fig 2).

**Fig 11:**
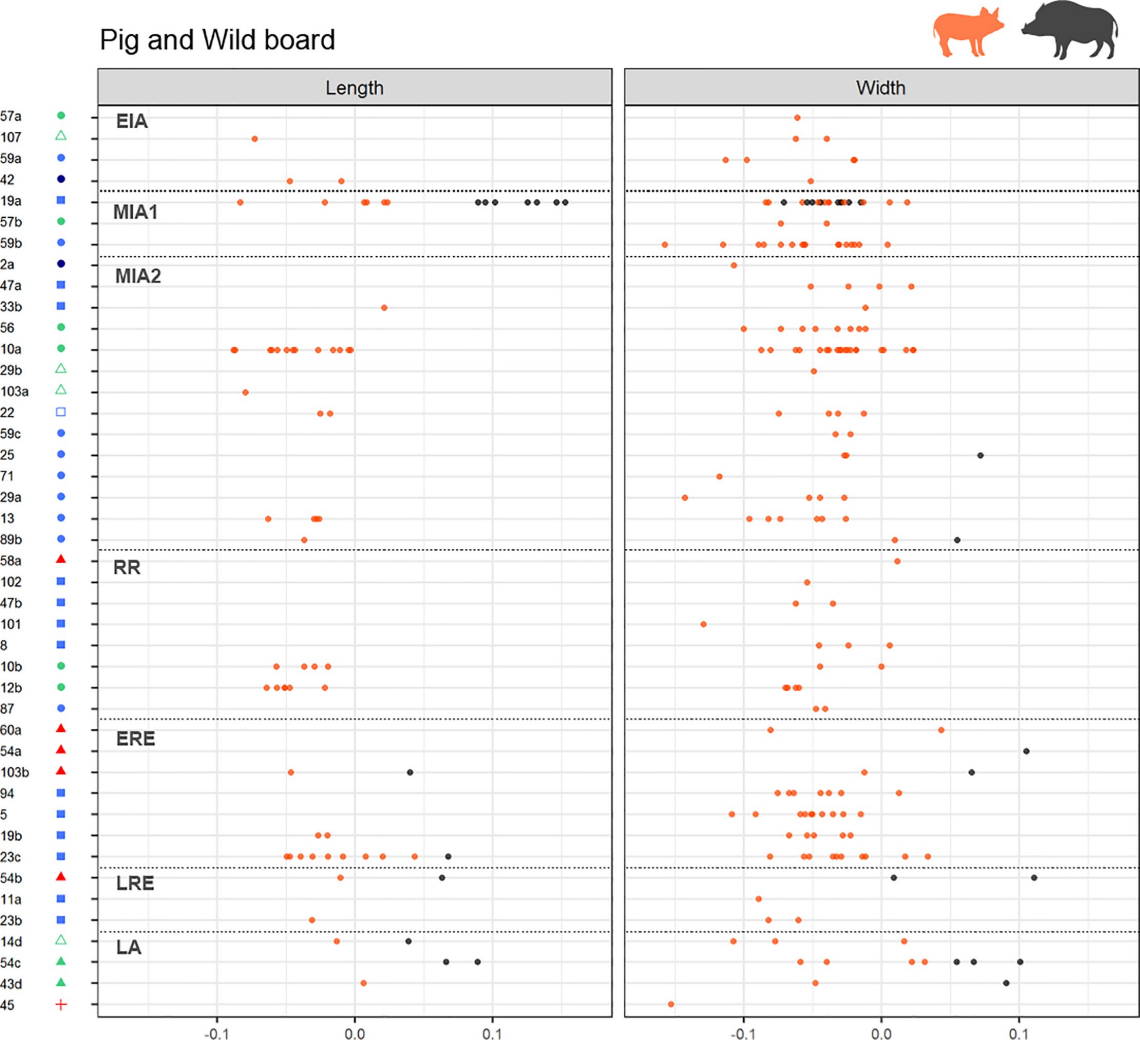
Pigs LSI length and width values by assemblages ordered by periods. (from top to bottom, EIA: Early Iron Age, MIA1: Middle Iron Age 1, MIA2: Middle Iron Age 2, RR: Roman Republic, ERE: Early Roman Empire, LRE: Late Roman Empire, LA: Late Antiquity). Left column shows length LSI values, right column shows width LSI values. Assemblages’ symbols refer to their type (see Fig 2).

Analysis of livestock biometry was based on 2354 (557 lengths and 1797 widths) unique post-cranial measurements (see S4 File). Figs 7 and 8 present LSI values by species, region and period, and Figs 9–11 show LSI values for each species at site level and period. Sheep and goat measurements were considered separately, plotted with different colors as similar trends, both on lengths and widths, and no significant differences were found between these two species (see S5 File). Full statistical results on the temporal variation of size are available in S6 File (table Mann-Whitney). In general terms, the number of available length measurements was very uneven between areas and periods, especially for CS and NC. Even so, our data were sufficient to highlight some general trends, which are presented below.

#### 5.4.1 Availability of biometric data and regional patterns

In some cases, the availability of biometric data is uneven depending on the type of measurement, the area, or the species. There were a lower number of effective lengths with respect to widths in all species. The pig was the most underrepresented species with regard to biometric data, especially in the area of OP and SC. Few studies have been done in the SC so far, which affected all main domestic species; here we simply included the currently accessible data so that it is recorded. Also for pigs, the protohistoric periods have many more gaps, in all areas, compared to the Roman periods (Figs 7 and 8): a fact which is due to the greater availability of pig remains in this moment. For cattle we have few lengths and widths in the case of the NC, and some small gaps in the case of the OP and the CC. Regarding sheep/goats there are some similar limitations to those of cattle, with large gaps for lengths in the NC, as well as for protohistoric periods in CC. However, a large set of width measurements helps to counteract these gaps.

Comparisons between zones show the first significant differences in **cattle** LSI values (for all statistical results see S7 in supplementary materials S7 File) occurring in the RR period, for both widths (p = 0.000) and lengths (p = 0.005). This difference is due to the presence of larger individuals in OP (see Figs 7 and 8). The same trend is observed in cattle width LSI values during LRE (p = 0.043).

On the other hand, intra-period analysis shows significant differences in LSI values from **sheep/goats** bone widths between OP and CC during EIA (p = 0.041, higher medians in CC), NC and CC in MIA1 (p = 0.004, higher medians in NC), SC and all other areas in MIA2. This latter difference is due to the low median in SC (Fig 8). During RR the sheep/goat biometric analysis shows a substantial difference between OP and CC in lengths (p = 0.017) due to the presence of larger individuals in CC (Fig 7). It should be noted the largest sheep/goat that appeared in NC during MIA1, with lengths equivalent to those of wild goats and domestic sheep and goats from the same area (Fig 7). The LSI values from **pig** bone widths only showed significant differences between OP and NC during MIA2 (p = 0.016); in this latter case, the difference can be related to the lack of biometric data for this species.

#### 5.4.2 Diachronic change by zone

*Cattle*. At a regional scale, cattle LSI values indicate a general cattle size increase from MIA1 up to ERE, followed by a size decrease during the Late Roman and Late Antiquity phases (LRE and LA), both in lengths and widths (Figs 7 and 8). In **the OP**, the only length measurement available for MIA1 attests the presence of at least one large individual of a similar height to the ones recorded in Roman times, which contrasts with the three measurements available for MIA2, all notably smaller. The median value of the LSI lengths and widths increased significantly during RR compared to MIA2 (Figs 7 and 8. For all statistical results see S5 in supplementary materials in S5 File). No significant changes were found between the different Roman periods in the OP. In **the CC,** no significant differences in width LSI values were found between MIA2–RR (p = 0.191), but two extraordinarily large individuals in RR are attested. A greater diachronic variability was visible during Roman times in both lengths (p = 0.000) and widths (p = 0.000), together with a height increase between RR–ERE. In contrast, the length values from LRE are significantly smaller than ERE ones (p = 0.034). Subsequent size decrease in LA (not significant) is attested on width values (p = 0.119). Published data for **the NC** are scarcer, but the available ones showed that there was a size increase between MIA1–MIA2, both in lengths (p = 0.005) and widths (p = 0.01), suggesting that during MIA2 there were some large individuals comparable to those from OP and CC during Roman times. The presence of large individuals contrasts with the short cattle attested in the other areas during MIA2.

*Sheep and goats*. Sheep and goats increased in size from the LBA to ERE, both in lengths and widths (Figs 7 and 8) followed by a size decrease during LA (Figs 7 and 8. For all statistical results see S5 in supplementary material in S5 File). Considering the different areas, an increase in the animals’ height is observed between MIA2–RR in **the CC** (p = 0.015) followed by a progressive decrease in widths during Roman times, and even more in LA. Width values also reflected a rapid growth from MIA1 to MIA2 (p = 0.039). The maximum median is attested during ERE, both on lengths and widths, and there was a subsequent size decrease from LRE to LA, most notably in widths (p = 0.001). In **the OP,** the median values show a progressive increase since LBA with a statistically significant difference between EIA and ERE for both lengths (p = 0.032) and widths (p = 0.000) (Figs 7 and 8). There are fewer data from **the NC**, but the width results suggest that there were no major differences between MIA1 and MIA2. In ERE, sheep and goats were significantly larger (or more robust) compared to Iron Age ones (p = 0.000 for MIA 1, p = 0.02 for MIA2). There is also evidence of a decrease in widths between ERE and LA (not significant, p = 0.11).

*Pig*. The LSI values from pig bone lengths (Figs 7 and 8. For all statistical results see S5 in supplementary material S5 File) do not reveal diachronic differences in any area, possibly due to the scarcity of data. LSI values from bone widths reveal a significant increase in size between MIA1–MIA2 in both CC (p = 0.083) and OP (p = 0.033). Unlike the other areas, **the OP** shows a significant decrease in pigs width between MIA2–RR (p = 0.042) that also occurred between ERE–LRE (p = 0.088, although there were few measures for LRE). In **the NC,** more data are necessary to draw any conclusion, but the data available for widths suggest that there were no significant changes in pig size between MIA and Roman times, as very stable median values are attested.

### 5.5 Changes in animal size by site type and period

The distribution of the LSI values from the main domesticates, according to the type of site in diachrony, shows that the general changes observed in the different zones (Figs 7 and 8) occurred in all the sites in a similar way, regardless of their typology (Figs 9–11). Only some sites stand out with some peculiarities, especially during the Iron Age (EIA and MIA) and Roman times (RR to LRE).

In MIA2, some sites of different typologies and located in different areas–i.e. Puig de Sant Andreu (88), Gou Batlle (22), Turó Font de la Canya (57c) and Vilars III-IV (59c)–stand out because of the presence of cattle individuals with large widths. Very large cattle lengths are also attested at Puig de Sant Andreu (88). Conversely, no differences in sheep/goats or pigs sizes are attested at those sites during MIA.

During the RR, some sites have LSI cattle bone lengths similar to those of the Iron Age, while others reveal the presence of much larger cattle–e.g. Burriac (8) in CC and Puig Castellar de Biosca (87) in OP. During ERE, a generalized cattle size increase is attested, and especially large cattle are recorded both in *villae* and in urban contexts (Fig 9).

The sheep and goat measurements dated to RR seem to be smaller in rural settlements with *silos* at OP–Rosella (84) and Missatges (92)–compared to other sites types close to them–e.g. Puig Castellar de Biosca (87)–and the coast silo fields at CC–Can Bartomeu (10b) and Can Feu (12b). On the other hand, LSI sheep/goat widths became larger in all sites during Roman Imperial times.

Pig width values seem to be decreasing during RR in most of the analyzed sites, with the exception of Burriac (8)–an indigenous site quickly romanized–and the *villa* of Vilarenc (58a) that had more robust individuals (Fig 11). During ERE width values are still low in most sites compared to the previous period. But there are some sites with more robust pigs, as in the urban contexts of *Ilerda*_Carrer Magdalena (23c) and Iesso–in the OP–, as well as in the rural site of Vilauba (60a) in the NC. On the other hand, lengths seem very stable throughout all periods, with the outstanding case of the urban context of Ilerda (23c), with larger-sized individuals during ERE. In all cases, these larger animals do not fall within the range of wild boars (Fig 11, black points), which suggests that they are larger domestic pigs.

Finally, during LA, all site typologies (both newly created and those with Roman levels), present smaller LSI lengths and **widths** for the main domesticates.

## 6. Discussion

The settlement patterns reflect human social organization and their impact over the landscape [8,165–168], and the study of the animal husbandry allows us to characterize the human subsistence strategies [169–171]. In the following paragraphs we will discuss the ecological and socio-economic reasons that probably influenced and conditioned livestock strategies, integrating the zooarchaeological data with those of other archaeological disciplines. For this purpose, a multiscale and diachronic analysis will be implemented, to be able to observe the nature and impact of changes at both a micro-spatial (site scale) and macro-spatial level (territory scale).

### 6.1. Impact of ecology on livestock patterns in the NE of the Iberian Peninsula

**Fig 12:**
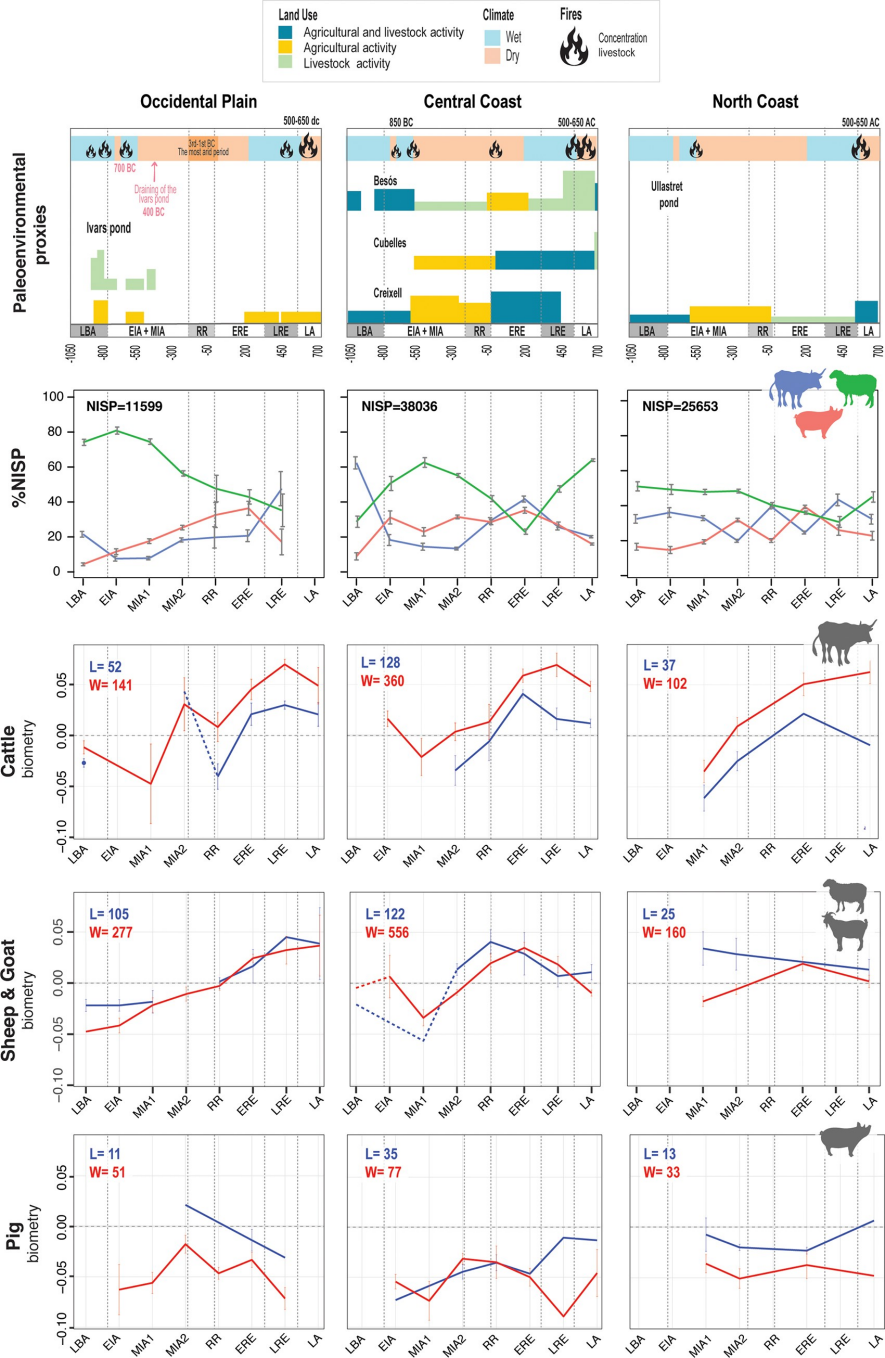
Synthesis of NISP frequencies and animal size for the main domesticates between Late Bronze Age (LBA) and Late Antiquity (LA) in the Occidental Plain (OP), Central Coast (CC) and North Coast (NC) compared to paleoenvironmental proxies. From top to bottom: paleoenvironmental proxies, main domestic NISP profiles (in percent), main domestic LSI lengths (in blue, Length) and widths (in red, Width) values with their total number of bones considered (see text for references).

Factors contributing to the degree of heat stress and seasonal effects on reproduction experienced by animals include environmental temperature, and landscapes less conducive to grazing (Fig 1B), solar radiation (Fig 1C), relative humidity (Fig 1D), and wind speed. These environmental factors produce changes in animal conformation, including the increase or decrease of their size and robustness (e.g. [172,173]). To complement the different ecological information of the studied areas provided by Figs 1 and 12 summarizes the ecological information available on fire incidence and landscape use based on palynological and sedimentological information on the region [81,82,174–179]. The integration of these different ecological data has made it possible to characterize which areas are potentially more or less suitable for grazing, taking into account the ethology of the different livestock species. This can be contrasted with the summary of NISP frequencies and animal size through time in the three areas for which we have reliable data (OP, NC, CC); data from the South Coast (SC) have not been considered in detail in this synthesis on account of the relatively scarce data available for the region (see e.g. Figs 3.1 and 5). In combination with Fig 6, we will discuss how the zooarchaeological results correlate with ecological differences between the areas.

Exploratory statistics on **livestock ratios** (Fig 6) suggest that during LBA and EIA there was an important correlation between the location, and hence ecological conditions of each studied area, and NISP frequencies at each site. During these times, geography, watersheds, potential local landscapes, solar radiation and annual rainfall (Fig 1) seem to have been the main factors influencing NISP frequencies. The OP and NC, the two study areas with the most dissimilar ecologies, show clearly different trends. The OP consistently correlates with higher frequencies of sheep and goats, and the NC, which is the wettest and the cloudiest of the four zones analyzed here, had higher cattle frequencies (Fig 5, and also Fig 12).

Environmental factors, and notably heat stress, are key determinants in cattle production (e.g. [173,180–182]), and the environmental situation in NC was likely more favorable for cattle production than the other study areas. The NC area is characterized by a complementarity between two types of climates (Fig 1B): an area near the coast characterized by a type Csa (Hot-summer Mediterranean climate; coldest month averaging above 0°C, at least one month’s average temperature above 22°C, and at least four months averaging above 10°C. At least three times as much precipitation in the wettest month of winter as in the driest month of summer, and driest month of summer receives less than 30 mm) and type Cfb (Temperate oceanic climate; coldest month averaging above 0°C, all months with average temperatures below 22°C, and at least four months averaging above 10°C; no significant precipitation difference between seasons). In addition, palynological and sedimentological studies show that the NC had large areas of wetlands until Roman times [175,177]. In this sense, the NC offers better conditions for cattle compared to the other areas, which are drier and warmer. The high percentages of cattle documented in the NC (between 30 and 40%) have also been attested in the neighboring area of Languedoc (France), which has an identical ecological setting [42,52,183].

In contrast, sheep and goats are better adapted to graze on the dry inland area, as they are more sensitive to humidity and bacteria, but are more resistant to solar radiation [184–186]. In addition, they prefer pastures and forested areas with different types of vegetation [187–190]. This is consistent with the greater correlation of sheep and goats in the OP, which is the driest of the four areas (Fig 1), with a climate that has been classified as cold and semi-arid (BsK). This climate type is typically found in continental interiors at some distance from large bodies of water, and it usually features warm-to-hot dry summers. It features major temperature swings between day and night, with dry summers, relatively wet winters, and even wetter springs and autumns (see Fig 1C). The CC, with greater solar radiation and lesser amount of rainfall than the NC, is strongly correlated with caprines and pigs. This area has a mixed ecology, with some regions closer to OP ecology (i.e. southwest zone, Bsk climate [57]) and others closer to NC (i.e. northeast zone, Csa and Cfb climate [57]). The general diversity of NISP profiles during LBA and EIA is consistent with the different ecological settings of the area.

These strong regional trends in livestock ratios, with statistically significant variations between ecological zones during LBA, EIA and MIA1, are also attested for LA assemblages (Figs 3.2 and 5). Interestingly, ecology does not seem to have the same influence in all periods. In this sense, during MIA2 there was a strong process of homogenization of livestock ratios in most sites, independent of the ecological zone or their typology (Fig 4, see also Fig 12). In addition, pig frequencies clearly increased during Roman times in all areas (Figs 5 and 12). This does not seem to be related to the ecological potentialities and/or limitations of each area, but rather reflects a global economic change driven by the well-known pig preference of Romans from central Italy [191–193]. This is mostly visible in urban contexts and the richest *villae*, which seem to be the most ’Romanized’ contexts.

Together with NISP frequencies, animal size also attests major changes in animal husbandry and may reflect adaptations to particular ecological requirements. As presented above, the results of **the biometric analyses** at the inter-regional level show (with exception of samples containing very few measurements) that mean LSI values from **cattle** widths and lengths increased from MIA1 to MIA2, and subsequently up to ERE, when the largest animals are documented (Figs 9 and 10). Both lengths and widths increased in a quite linear way, which suggests that the size change relates either to a better nourishment of the animals, import of larger animals that have a similar body conformation, or both. In any case, detailed examination of the robustness of metacarpals [194] suggests that the increase of cattle size was not related to a different sex ratio or castration practices. At the sub-regional level, the increase of cattle size happened earlier in the OP compared to CC (see Figs 9 and 10, and S5 in supplementary material in S5 File). This cannot be related with better ecological conditions in the OP, which is the driest of the four zones analyzed (Fig 1). Size increase during RR in OP is not incidental either, as large cattle in OP have the same size as those recorded during ERE. Consequently, the OP provides a clear example that, in some periods, human decisions and technical capacities may have a larger impact on animal morphology than purely the ecological setting.

This is also visible in **sheep and goats**, which experienced a similar trend, with a general size increase from LBA to Roman times, and a later decrease of size during LA, visible in both lengths and widths. At the sub-regional level, the NC displays wide variation in sheep/goat size during MIA1 (Fig 7). This could be the consequence of the presence of wild animals identified as domestic sheep/goats, although the importation of animals cannot be completely excluded. The SC had smaller and slender caprines compared with the other areas (Figs 7 and 8). These size differences between zones cannot be solely explained by environmental conditions like rainfall or solar radiation, as the same size increase process is attested in different ecological zones–OP and CC–early after the Roman conquest (RR).

Trends observed in **pigs** suggest a greater variability between zones. In OP the LSI values of widths decrease in two moments: from MIA2 to RR, and from ERE to LRE. Length data are scarce, but there are also hints–as happens with the other two species–, that before the Roman conquest (MIA2) there were larger pigs in OP than in the other areas. It should also be noted that in OP during the RR there is also a significant decrease in the robustness of animals, as is also observed in sheep/goats and cattle. This trend, which is only observed in OP, could indicate a different impact of the first moments of Romanization with respect to the other areas. On the other hand, the CC shows a progressive increase in the size of the pigs from EIA to RR–ERE, with hints of large pigs during LRE and LA. The LSI values of widths show a progressive decrease in the robustness of the animals from MIA2 to LRE, with an important change during LA with individuals of greater robustness. In this regard, it should be noted that there is also a decrease in the robustness of pigs from ERE to LRE in the other areas. The scarce information for the NC only allows us to say that a very different trend is apparent, with a progressive and slight decrease of pig from MIA1 to ERE, and the presence of larger size pigs during LA.

**Fig 13:**
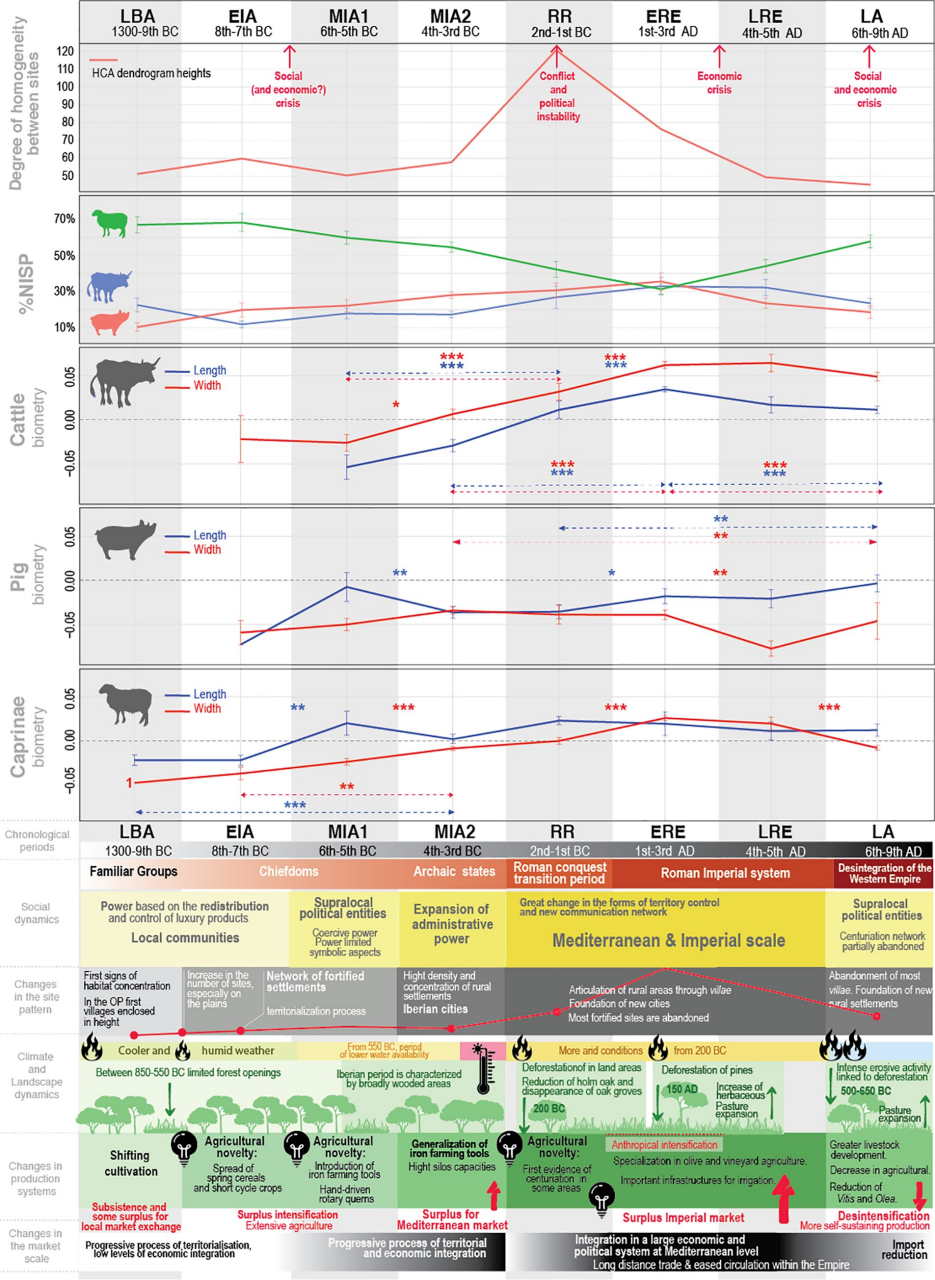
Synthesis of the NISP percentages and size change of the main domesticates (top) compared to other proxies (bottom) in Catalonia between Late Bronze Age (LBA) and Late Antiquity (LA), from left to right, LBA: Late Bronze Age, EIA: Early Iron Age, MIA1: Middle Iron Age 1, MIA2: Middle Iron Age 2, RR: Roman Republic, ERE: Early Roman Empire, LRE: Late Roman Empire, LA: Late Antiquity. Chi-square results showing significant changes between periods are displayed with their *p-value*: ***: highly significant (< 0.01); **: significant (< 0.05); * less significant (< 0.1).

Our results suggest that the size increase does not seem to be linked to those periods with more water availability or a more conducive climate. In this respect, in the OP, the LBA and MIA1 correspond to the wettest periods and with more deforestation activity, in relation to documented regional fires (Figs 12 top and 13 bottom) linked to the intensification in agriculture and animal herding (Fig 13 bottom) [82], and this is also documented in the pre-littoral plain (CC) [81]. Despite the potential for more arable land and water availability, cattle frequencies decreased over these time periods, together with animal size (Fig 12), following a tendency consistently observed from the LN and early Bronze Age [45,195]. Conversely, the transition from MIA1 to MIA2 suggests an increase in animal size before the Roman conquest (or at least the presence of some large animals), coinciding with a period of maximum drought (Fig 12 top), and evidence of an intensification of land use for agriculture (Fig 13 bottom) in most areas studied [82,176,178] (Fig 12 sup). As we will discuss below, this also coincided with the maximum territorial expansion of Iberian Archaic states (Fig 13 bottom) [12,27,99,106,107]. Another evidence suggesting that the increase in size does not appear to be solely linked to periods with a more favorable ecological context, is the divergent patterns of cattle and pig size change in the different areas during the same episode of maximum drought in Catalonia between the 3rd and the 2^nd^ c. BC [176,178] (Figs 12 top and 13 bottom). While there was a significant increase in the cattle size in the OP (Figs 7 and 8), in CC no major changes are attested from MIA2 to RR based on the data currently available.

Overall, the evidence from NISP and biometry suggests that the changes in animal husbandry were only partially related with regional environmental and/or ecological factors. Beyond the particular inter-regional dynamics observed during protohistoric periods, the results show a general tendency for common diachronic change in all areas from MIA2. Some size change patterns occur in all sites, regardless of their typology, including both urban centers and small rural settlements with only some exceptions (see results section). Other works have previously documented the same processes of progressive augmentation of cattle size from the Iron Age to Roman times in other European countries with very different ecologies, and related to the development of market economies (e.g. [43,45,50,196,197] and references within). Consequently, size change and different livestock ratios did not result from a universal or external factor (e.g. a general climatic change) but seems closely related to cultural and economic decisions as discussed below.

### 6.2 Changes in animal husbandry, demographic growth and technological innovation

Fig 13 summarizes the main socio-political events attested for the NE of the Iberian Peninsula between the Late Bronze Age and Late Antiquity, together with a synthesis of the main zooarchaeological and ecological data available.

The progressive increase of pig frequencies during **LBA** (Fig 13) is concurrent with the greater territorialization and evidence of a demographic increase, visible in, for example, the number of graves in the necropolises [27,85] and the colonization of new lands [13,28,67,71]. According to some authors, this demographic increase, together with the colonial stimulus, increased the pressure on the resources [27]. Such pressure promoted consolidation of territorial control to guarantee access to resources and probably led to a progressive decrease in the land directly accessible for each settlement. The diversity observed in the livestock ratios between sites and areas (Figs 3.1, 4 and 12) suggest that each site chose the most sustainable livestock model and prioritized the domestic species better adapted to its immediate environment. The small size of animals suggests a strategy based on community self-sufficiency but with a possible stock surplus complementary to that of surrounding regions, and it could also reflect the prioritization of more adaptable and less demanding animals on fresh pasture [194]. Consequently, our results are consistent with the population model and social organization suggested for this area, in which each village would constitute an economically self-sufficient population cell–to a notable extent–that would manage and operate on the surrounding territory according to the membership of a human group for kinship relationships [13,27,28,198–200].

Some authors have defined the **EIA** as a period of continuity (e.g. [13,28,68]), but livestock frequencies are significantly grouped by site type in this period. The fortified sites of the OP–with a greater urban complexity–had higher percentages of pigs, and the sites associated with silos fields in CC and NC–that attest of a significant presence of imported products–had notable percentages of pigs and cattle, constituting a fairly homogeneous group (Figs 4 top and 6). This pattern is also present at the aristocratic residence of Sant Jaume Mas d’En Serrà_sect. 1 (42). Pigs and cattle are the two species with higher meat yield per animal. Considering the functionality of these sites–e.g: Vilars 0-I (59a), Can Roqueta (15c), Turó Font de la Canya 0 (57a), Sant Jaume Mas d’En Serrà_sect. 1 (42)–and their significant quantities of colonial products such as amphorae, tableware related to wine consumption and other sumptuous objects, this NISP profile may be related to collective meat and wine consumption during feasting practices in order to reinforce social and territorial cohesion within and between groups [201–205]. This seems consistent with the evidence of territorial authorities, growing social hierarchy, and the impact of exchanges and redistribution of Mediterranean products on local communities. In addition, the higher frequency of pigs can also be related to the increased pressure on the landscape and the demographic increase, as pigs are prolific and efficient meat producers, and can feed in marginal forest areas and domestic refuse [206,207]. In addition, the demographic growth and grain farming may have reduced the pastures available for cattle and caprines (per animal/herd). Also in this period, the maximum storage capacity transcends, for the first time, the scale of domestic consumption in some sites, reaching profiles that are compatible with the production of a surplus for exchange [103,105]. The profiles of the domestic triad are consistent with a greater territorialization and competitive processes for the exploitation of the best resources and the control of pastures and/or routes of circulation of colonial products. It may also reflect the progressive integration of the area in larger Mediterranean markets.

From the middle of the 6^th^ c. BC **(MIA1)** and up to the 3^rd^ c. BC **(MIA2)** the evidence of social hierarchy was increasingly reflected both in the necropolises (the number of tombs is reduced but the grave goods are richer) and in the settlement pattern (diversification of the sites types, and spread of the fortified settlements (FS) that constituted the centers of power [27,84,91,208]. This period has been described as a moment of transition from a regional system (MIA1: 6^th^–5^th^ c. BC) and the expansion of an administrative system with the appearance of the first archaic states in the region (MIA2: 4^th^–3^rd^ c. BC). The increase of animal size coincided with a period of maximum drought, the intensification of land use for agriculture in most areas studied [82,176] (Fig 12 top), and also with the moment of maximum territorial expansion of the Iberian states [28,99,106]. A homogenization of livestock ratios during this period is also well attested (Figs 3.1, 4 and 13 top), and some authors have linked this pattern to an increase in interdependence between sites and the influence of the aristocratic and military elites on animal production [29,30,53]. The decrease in the percentage of cattle and the increase in their size and robustness (Fig 13) is also consistent with a more intensified agricultural system where cattle are selected to obtain more efficient individuals for work. In parallel, technological innovations such as the generalization of iron tools for agricultural work, the appearance of rotary mills from 5^th^ c. BC [27,71,102,209–211], and emergence of wheel-thrown pottery show an intensification in harvest production and post-harvest transformation/conservation. The spread of cereal farming with probable larger areas under cultivation [212]–observed also on pollen and anthracological analyses (e.g. [213–215])–, could have led to a reduction of forest resources with a limitation of the range of movement of pigs [206], and the opportunities for domestic pigs to interbreed with larger wild boar [51], contributing to their size reduction. These results are consistent with the introduction, from the end of the 4^th^ c. BC, of an Eurasian model of agriculture, based on permanent land use and sectoral fallow land [71,216–218]. The existence of a centralized administrative power, of technological advances (which could include improvements in working animals) and the availability of local labor (demographic growth), would have favored a production increase, allowing a production surplus with speculative and commercial purposes (e.g. [27,103,105]).

The moment of maximum expansion of the Iberian states coincided with the beginning of **the Roman conquest (RR)** in the NE of the peninsula. The majority of faunal assemblages from this period date from the 2^nd^ c. BC, when Romans imposed heavy taxes on the allies (e.g. [219,220]) in a period of strong instability due to the constant rebellions of the different Iberian peoples as the *Ilergetes* (in 206 and 205 BC: *Polybius*, *11*, *32*), *Sedetani* (200 BC: *Livy*, *31*, *49*, *7*), *Indigetes* (195 BC: *Appian*, *Iberia*, *40*). The indigenous territorial and political system was maintained at first and some *oppida* persisted–e.g. Sigarra (47b), Burriac (8)–but there was a general abandonment of the fortified settlements–e.g. Puig de Sant Andreu (88), Alorda Park (2), Penya del Moro (38), Puig Castellar (25). In addition, new open and small rural settlements devoted to agricultural production and with some associated silos were built–e.g. Missatges (92), Hereuet (91), la Rosella (84), Can Bartomeu (10b)–[108,109,112,116–121], perhaps to face the heavy taxes imposed by Rome and supply of the Roman army present in the area. The great diversity observed, both in the types of sites and in the frequencies of the main domesticates (Figs 3.2 and 4), seems to reflect this period of war and instability, in which each territory/site had different responses and adaptations to the invasion. In this context, there are divergent patterns of animal size in the different areas during the same episode of maximum drought between the 3^rd^ and 2^nd^ c. BC [176,178] (Fig 12). In CC and NC, there was an increase of cattle and sheep/goat sizes from MIA2 to RR. This probably reflects the introduction of new animal morphotypes during the Romanization process, as previous works on the NC have suggested [221]. In the same period, the significant decrease in the width values of animals observed for cattle and pigs in OP and CC could indicate a higher degree of economic disruption, for example due to military conflicts in the area.

During Roman Imperial times (**ERE)** a new settlement pattern is attested in the region, seen in the concentration of dispersed settlements and the spread of the *villae* system. This new and specialized farming system had a fundamental role in territorial structuring [129]. In addition, large urban centers appeared with high population densities, and they concentrated the political and administrative powers of the provinces [114,222]. The drastic reduction of sheep/goat frequencies and the rise of pig (meat producers that could feed in forests or courtyards)–is concurrent with a time of greater specialization and intensification of food production, as observed on pollen analyses (e.g. [82,176,178,223]). The high frequencies of pigs also could reflect the well-known dietary preference for pork of Roman populations from central Italy [191–193]. Interestingly, high pig frequencies are mainly attested in urban centers and the richest *villae* (Figs 3.2 and 4). This phenomenon has also been observed in Britain [191,224] and other European territories (e.g. [225] and references within). In addition, some *villae* display high cattle frequencies, perhaps related to their role as a working animal [226–229]. The increase of cattle size is well attested in all kinds of sites, irrespective of their typology (Fig 9). On the other hand, sheep/goats and pigs management show a different tendency, as their size stabilized and even decreased during Roman times (Fig 13). Further research is needed to explain this divergent phenomenon. Nonetheless, our results are consistent with the existence of a large-scale productive system with specialized livestock productions. The Roman economic system was sustained on a high index of connectivity and large-scale and long-distance road and commercial network that provided the supply and free movement of products and people within the limits of the empire (e.g. [17]). Strontium isotopic results on livestock suggest this also included animals [52,230,231].

During **the LRE period** there was a substantial reduction in the total number of settlements [129–131]. In addition, there was a significant change in the interactions between urban and rural sites. The drastic reduction in imports and the size of production facilities in many *villae* [61,129,142,147] is considered to reflect a time of economic crisis as a result of the decline of large-scale Mediterranean trade [114,152]. The zooarchaeological results are consistent with a decline of the Roman imperial system. First, there was a drastic decrease of pig frequencies compared to the previous period (Fig 13), most notably in the cities (Figs 3.2 and 4). Second, this was coupled with increased frequencies of caprines and cattle at most sites (Fig 13). The prioritization of these two taxa reflects more extensive livestock models, where the species best adapted to local landscapes are selected, but which are also those with the highest land costs (greatest need for pasture). In cities, the decline in pigs relates to changing urban supply systems, which became less concerned with surplus meat provisioning to the non-farming consumers that lived in cities, due to a decline urban population size and probably also living standards [193]. Therefore, these profiles provide the first indexes of de-intensification processes of agricultural production, consistent with a context of lower population density, as suggested by the reduction in the number of settlements. The livestock ratios are consistent with a strategy of greater self-sufficiency and perhaps a way back to more extensive livestock production, linked to the exploitation and use of local natural resources as suggested by the increase in caprines ratios. Palynological and micro-charcoal studies attest to a higher presence of fires, which probably aimed to create pasture areas during this period [176]. There was also a significant increase in the degree of homogeneity between sites, thus suggesting a decrease in specialized sites in addition to the general decline of production. This is also consistent with the reduction of animal size (Fig 13).

The **Late Antique period (LA)** marked a new structuring of settlement patterns, with the continuity of some sites–which underwent major transformations [144,146,148,149]–and the building of new settlements adapted to a new political and economic structure, as well as to new ideological needs [132]. In this context, the frequency of caprines increased compared to pigs and cattle, most notably in the CC area (Figs 5, 12 and 13). This suggests an expansion of more extensive livestock systems, more adapted to the ecology of the different environmental zones and better suited to exploitation of marginal areas. In this regard, most sites have livestock profiles that are reminiscent of those observed during the LBA and EIA (Figs 3.1, 3.2 and 4), when the economic system is thought to be significantly more self-sufficient and territorialized [129,142,149,152,232]. Cattle, which had already experienced a significant decline in size during late Roman times, decreased even further, and sheep and goats became less robust. Our results are consistent with a more sustainable and smaller-scale livestock model in a context of large-scale trade recession. Human communities focused on a strategy based on self-sufficiency, prioritizing the most resilient species for the region. Intense erosive activity and evidence of forest burning [178,223,233] also suggests the creation of pastures for extensive herding and, overall, an agricultural model adapted to a less intensified and more local economic system. This is consistent with a significant decrease in the interdependence between sites.

## 7. Conclusions

The archaeozoological meta-analysis presented here provides a general perspective on the evolution of animal husbandry over c. 1700 years in the NE of the Iberian Peninsula, taking into account the ecological characteristics of four different areas in present-day Catalonia. This broad diachronic perspective was necessary to elucidate whether animal husbandry practices were mainly related to the ecological characteristics and climatic changes in the region, or were the result of socio-economic processes. The zooarchaeological results suggest that, although the ecological character of the different zones played an important role, livestock frequencies and changes in animal size are mostly related to socio-economic choices. The impact of ecology on animal husbandry fluctuated over time in direct relation to the specific socio-economic circumstances of each cultural period. The periods with a lesser degree of economic integration–with a more fragmented political and territorial system, and with production more focused on supplying local markets (i.e. Late Bronze Age, Early Iron Age and Late Antiquity)–displayed husbandry strategies closely related to the ecological conditions of each area, thus maintaining a better balance between the carrying capacity of the local environment and the needs of the population. Their particular ecologies allowed them to generate some surplus–more fitting with their own environmental conditions–that could be traded with neighboring sites and/or in the local markets. During these periods, the sites located in the Occidental Plain (OP) and the south coast (SC) focused on the extensive grazing of sheep and goats, sites in the north coast (NC) display higher frequencies of cattle, and sites in the central coast (CC) balance between caprines and pigs. In contrast, this link between livestock exploitation and the environment is less apparent during periods when production turned towards an increasingly pan-Mediterranean market economy, which mainly happened during the 4^th^–3^rd^ c. BC and Roman times. These two periods (Iron Age and Roman times) display two different models of animal production: the Iron Age strategy focused on sheep and goats, and the Roman model focused on pigs and cattle. This probably shows a cultural choice but also a radical change of demand and production, that was not conditioned any more by regional environmental differences but by a large Mediterranean market with access to a variety of productive environments.

The study also reveals that animal size significantly changed through time and, again, this was not linked to local ecological conditions but the degree of economic integration. In the Roman period, surplus production focused on specific local products (most notably wine). This, in combination with the important increase in the number and size of cities in the area, the increase in the non-productive sector of the population, and Roman colonial land distribution practices, facilitated the adoption of large-scale agricultural production linked to the expansion of first rural *fundi* and, later on, proper *villae*. This can be related to the significant increase in size and robustness of cattle. The influence of the political and economic context is also apparent during Late Antiquity, when smaller political units and more autarkic modes of production were put in place. This is consistent with the decrease in cattle size (i.e. small cattle are better suited to small-scale economic units/small scale-exploitation strategies) as well as the prioritization of more extensive, resilient livestock systems, as shown by the increase of sheep and goat frequencies.

This study contributes to the understanding of the dynamics of landscape exploitation between the Late Bronze Age and Late Antiquity in NE Iberia, and offers a valuable foundation to further explore the impact of socio-political systems on animal husbandry over time. Overall, our results show that human communities adapted animal husbandry to their social, political, and economic environment, as well as the physical landscape. Within each chronological period, the capacity of societies to implement the most productive and most intensive economic systems–which are not always the most sustainable in the long term–depended on context-specific factors. These include technical innovations, production and consumption needs, and market demands, which are shaped by the existing political system, availability of labor, and technology. In charting the impact of these factors on animal production, zooarchaeology proves to be a key discipline to explain and characterize socio-economic and political changes through time.

## Supporting information

S1 Checklist PRISMA 2009 flow diagram. (DOC)

S1 File Studied dataset, archaeological sites with periods, geographical areas, total of livestock NISP and associated references. (PDF)

S2 File Maps of the studied area by period with site locations by types and geographical areas [55]. (PDF)

S3 File Chi-squared results on livestock NISP by periods, type of site (left aligned) and geographical areas (right aligned).
(TIFF)

S4 File Summary table of the length and width measurements ordered by taxa with the geographical areas and periods. (XLSX)

S5 File Mann-Whitney pairwise results between periods for taxon’s length and width measurements grouped by geographical areas. P-values significant differences: ***: highly significant (< 0.01); **: significant (< 0.05); * less significant (< 0.1).
(XLSX)

S6 File Mann-Whitney pairwise results between periods for taxon´s length and width measurements for the whole studied area. (PDF)

S7 File Mann-Whitney pairwise results between geographical areas for taxon´s length and width measurements grouped by periods. (PDF)

## References

[1] Collis J. The European Iron Age. Taylor&Francis. London and New York: Routledge; 2003.

[2] Cunliffe B. The Ancient Celts, Second Edition Oxford University Press; 2018.

[3] Lichardus-Itten M, Lichardus J. La protohistoire de l’Europe: Le Néolithique et le Chalcolithique entre la Méditerranée et la mer Baltique. Presses Universitaires de France; 2018.

[4] Guilaine J. La mer partagée. La Méditerranée avant l’écriture, 7000–2000 avant Jésus-Christ. Paris: Hachette; 1994.

[5] Hodder I. The Domestication of Europe | Wiley. New Jersey, United States: Wiley-Blackwell; 1991 10.1111/j.1432-1033.1991.tb15993.x

[6] Kristiansen K. Europe Before History. Cambridge University Press; 2000.

[7] Py M. Evolution des rapports sociaux de la fin de l’Âge du Bronze à la conquête romaine en Languedoc oriental. Collection de l’Institut des Sciences et Techniques de l’Antiquité. 1984;290: 171–184.

[8] Johnson AW, Earle TK. The Evolution of Human Societies: From Foraging Group to Agrarian State. Stanford University Press; 2000.

[9] Py M. Les gaulois du midi, De la fin de l’age du bronze a la conquete romaine. Paris: Hachette; 1993.

[10] Brun P. From chiefdom to state organization in Celtic Europe In: Arnold B, Gibson DB, editors. Celtic chiefdom, Celtic state The evolution of complex social systems in prehistoric Europe. Cambridge, United Kingdom: Cambridge University Press; 1995 pp. 13–155.

[11] Asensio D, Belarte C, Sanmartí J, Santacana J. Paisatges ibèrics: Tipus d’assentaments i formes d’ocupació del territori a la costa central de Catalunya durant el període ibèric ple In: Aranegui C, editor. Los Iberos, príncipes de Occidente: las estructuras de poder en la sociedad ibérica. València: Universitat de València; 1998 pp. 373–385.

[12] Sanmartí J, Belarte C. Urbanización y desarrollo de estructuras estatales en la costa de Cataluña (siglos VII-III aC) In: Berrocal-Rangel L, Gardes P, editors. Entre celtas e íberos: las poblaciones protohistóricas de las Galias e Hispania. Madrid, Spain: Real Academia de la Historia, Casa de Velázquez; 2001 pp. 161–174.

[13] López-Cachero FJ. Sociedad y economía durante el Bronce Final y la primera Edad del Hierro en el Noreste Peninsular: una aproximación a partir de las evidencias arqueológicas. Trabajos de Preshistoria. 2007;64: 99–120.

[14] Kristiansen K. Interpreting Bronze Age Trade and Migration. Cambridge, United Kingdom: Cambridge University Press; 2016 pp. 154–180.

[15] Remesal. Betican olive oil and the Roman economy In: Keay S, editor. The Archaeology of early Roman Baetica. Universitat de Michigan: Journal of Roman Archaeology; 1998 pp. 183–199.

[16] Revilla V. Producción cerámica y economía rural en el bajo Ebro en época romana: el alfar de l’Aumedina, Tivissa (Tarragona). Barcelona: Edicions Universitat Barcelona; 1993.

[17] Ward-Perkins B. The Fall of Rome: And the End of Civilization. Oxford: Oxford University Press; 2006.

[18] Keay S, editor. Rome, Portus and the Mediterranean. London: British School at Rome; 2012.

[19] Wilson A. Approaches to Quantifying Roman Trade In: Bowman A, Wilson A, editors. Quantifying the Roman Economy: Methods and Problems. Oxford: Oxford University Press; 2009 pp. 213–249.

[20] Orengo HA, Livarda A. The seeds of commerce: A network analysis-based approach to the Romano-British transport system. Journal of Archaeological Science. 2016;66: 21–35. 10.1016/j.jas.2015.12.003

[21] Sarris P. The Origins of the Manorial Economy: New Insights from Late Antiquity. Engl Hist Rev. 2004;119: 279–311. 10.1093/ehr/119.481.279

[22] Banaji J. Agrarian Change in Late Antiquity: Gold, Labour, and Aristocratic Dominance: Gold, Labour, and Aristocratic Dominance. Oxford: Oxford University Press, UK; 2002.

[23] Christie N. Landscapes of Change: Rural Evolutions in Late Antiquity and the Early Middle Ages. Routledge; 2017.

[24] Garcia D. Observations sur la production et le commerce des céréales en Languedoc méditerranéen durant l’Age du Fer: les formes de stockage des grains. Revue archéologique de Narbonnaise. 1987;20: 43–98. 10.3406/ran.1987.1306

[25] Sanmartí J. From local groups to early states: the development of complexity in protohistoric Catalonia. Pyrenae. 2004; 7–41.

[26] Gailledrat E. Espaces coloniaux et indigènes sur les rivages d’Extrême-Occident méditerranéen. Montpellier: Presses universitaires de la Méditerranée; 2014 10.1038/gt.2013.82

[27] Sanmarti J, Santacana J. Els ibers del Nord. Rafael Dalmau. Barcelona; 2005. Available: https://www.alibri.es/els-ibers-del-nord-306952.

[28] López-Melción JB. L’Evolució del poblament protohistòric a la plana occidental catalana: models d’ocupació del territori i urbanisme. TDX (Tesis Doctorals en Xarxa). 2000 [cited 17 Nov 2019]. Available: https://repositori.udl.cat/handle/10459.1/63724.

[29] Valenzuela-Lamas S. Alimentació i ramaderia al Penedès durant la protohistòria: segles VII-III aC. Societat Catalana d’Arqueologia; 2008.

[30] Nieto-Espinet A. Entre el consum i l’afecte. La interacció entre els animals i les comunitats protohistòriques de la plana occidental catalana (segles VII—IV a.C). PhD, Universitat de Lleida. 2012.

[31] Colominas L, Fernández-Rodríguez CF, Iborra MP. Animal Husbandry and Hunting Practices in Hispania Tarraconensis: An Overview. European Journal of Archaeology. 2017;20: 510–534. 10.1017/eaa.2016.30

[32] Matolcsi J. Historische Erforschung der Körpergröße des Rindes auf Grund von ungarischem Knochenmaterial. Zeitschrift für Tierzüchtung und Züchtungsbiologie. 1970;87: 89–137. 10.1111/j.1439-0388.1970.tb01330.x

[33] Bokonyi S. History of Domestic Mammals in Central and Eastern Europe. Akademiai Kiado; 1974.

[34] Altuna J. Historia de la domesticación animal en el País Vasco desde sus orígenes hasta la romanización. Munibe Antropologia—Arkeologia. 1980; 9–163.

[35] Ijzereef GF, Van Regteren Altena J-F, Kuijper WJ. Bronze age animal bones from Bovenkarspel the excavation at Het Valkje. Amersfoort: ROB; 1981.

[36] Méniel P. Contribution à l’histoire de l’élevage en Picardie. Du néolithique à la fin de l’Âge du Fer. Revue archéologique de Picardie. 1984;3: 1–56. 10.3406/pica.1984.3126

[37] Vigne J-D. Les mammifères post-glaciaires de Corse. Étude archéozoologique. Editions du Centre National de la Recherche Scientifique Gallia Préhistoire. Paris: Persée—Portail des revues scientifiques en SHS; 1988.

[38] Audoin-Rouzeau F. La taille du boeuf domestique en Europe de l’Antiquité aux temps modernes. Juan-les-Pins: APDCA; 1991.

[39] Lepetz S. Effets de la romanisation sur l’élevage dans les établissements ruraux du Nord de la Gaule: l’exemple de l’augmentation de la stature des animaux domestiques. Revue archéologique de Picardie. 1996;11: 317–324. 10.3406/pica.1996.1903

[40] Peters J. Römische Tierhaltung und Tierzucht: Eine Synthese aus archäozoologischer Untersuchung und schriftlich-bildlicher Überlieferung. Rahden/Westf: Leidorf; 1998.

[41] Breuer G, Rehazek A, Stopp B. Grössenveränderungen des Hausrindes: osteometrische Untersuchungen grosser Fundserien aus der Nordschweiz von der Spätlatènezeit bis ins Frühmittelalter am Beispiel von Basel, Augst (Augusta Raurica) und Schleitheim-Brüel. Jahresberichte aus Augst und Kaiseraugst. 1999;20: 207–228. 10.5169/seals-395607

[42] Forest V, Rodet-Belarbi I. Á propos de la corpulence des bovins en France durant les périodes historiques. Gallia. 2002;59: 273–306.

[43] Frémondeau D, Nuviala P, Duval C. Pigs and Cattle in Gaul: The Role of Gallic Societies in the Evolution of Husbandry Practices. European Journal of Archaeology. 2017;20: 494–509. 10.1017/eaa.2016.10

[44] Valenzuela-Oliver A, Alcover JA, Cau MÁ. Tracing changes in animal husbandry in Mallorca (Balearic Islands, Western Mediterranean) from the Iron Age to the Roman period In: Groot M, Lentjes D, Zeiler J, editors. Barely surviving or more than enough? Sidestone Press; 2013 pp. 201–223.

[45] Valenzuela-Lamas S, Albarella U. Animal Husbandry across the Western Roman Empire: Changes and Continuities. European Journal of Archaeology. 2017;20: 402–415. 10.1017/eaa.2017.22

[46] Duval C, Clavel B. Bœufs gaulois et bœufs français: morphologies animales et dynamiques économiques au cours de La Tène et des périodes historiques. Gallia Archéologie des Gaules. 2018;75: 141–171. 10.4000/gallia.3904

[47] Clutton-Brock J. Domesticated animals from early times. Domesticated animals from early times. 1981 [cited 26 Jul 2020]. Available: https://www.cabdirect.org/cabdirect/abstract/19820166164.

[48] Davis SJM. The effects of temperature change and domestication on the body size of Late Pleistocene to Holocene mammals of Israel. Paleobiology. 1981;7: 101–114. 10.1017/S0094837300003821

[49] Manning K, Timpson A, Shennan S, Crema E. Size Reduction in Early European Domestic Cattle Relates to Intensification of Neolithic Herding Strategies. PLOS ONE. 2015;10: 1–19. 10.1371/journal.pone.0141873 26630287 PMC4668083

[50] Duval C, Lepetz S, Horard-Herbin M-P. Diversité des cheptels et diversification des morphotypes bovins dans le tiers nord-ouest des Gaules entre la fin de l’âge du Fer et la période romaine. Gallia. 2012;69: 79–114.

[51] Trentacoste A, Nieto-Espinet A, Valenzuela-Lamas S. Pre-Roman improvements to agricultural production: Evidence from livestock husbandry in late prehistoric Italy. PLOS ONE. 2018;13: e0208109 10.1371/journal.pone.0208109 30596652 PMC6312331

[52] Nieto-Espinet A, Valenzuela-Lamas S, Bosch D, Gardeisen A. Livestock production, politics and trade: A glimpse from Iron Age and Roman Languedoc. Journal of Archaeological Science: Reports. 2020;30: 102077 10.1016/j.jasrep.2019.102077

[53] Albizuri S, Nieto-Espinet A, Valenzuela-Lamas S. Canvis en l’alimentació càrnia a Catalunya entre els segles XII i III aC. Saguntum: Papeles del Laboratorio de Arqueología de Valencia 2010; 162–171.

[54] Colominas L. Roman Conquest and Changes in Animal Husbandry in the North-East of the Iberian Peninsula: Searching for Patterns, Rates and Singularities. Valenzuela-Lamas S, Colominas L, Fernández-Rodríguez C, editors. A. 2017;26: 9–22. 10.15366/archaeofauna2017.26.001

[55] Open Street Map (OSM). Geofabrik Download Server. 2020 [cited 3 Aug 2020]. Available: http://download.geofabrik.de/.

[56] Survey (USGS) USG. 30-meter resolution elevation data from the Shuttle Radar Topography Mission version 3.0 (SRTM30 v.3.0). 2015 [cited 1 Sep 2019]. Available: https://dds.cr.usgs.gov/srtm/version2_1/SRTM30/w020n90/.

[57] Beck HE, Zimmermann NE, McVicar TR, Vergopolan N, Berg A, Wood EF. Present and future Köppen-Geiger climate classification maps at 1-km resolution. Scientific data. 2018;5: 180214 [cited 1 Jan 2021]. Available: https://zenodo.org/record/3660068. 10.1038/sdata.2018.214 30375988 PMC6207062

[58] Fick SE, Hijmans RJ. WorldClim 2: new 1-km spatial resolution climate surfaces for global land areas. International journal of climatology. 2017;37: 4302–4315. [cited 20 Dec 2020]. Available: http://www.worldclim.com/current.

[59] Folch C. El poblament al nord-est de Catalunya durant la transició a l’edat mitjana (segles V-XI dC). Annals de l’Institut d’Estudis Gironins 2005; 37–68.

[60] Gurt JM, Navarro R. Maria J, & Navarro R. (2005). Les transformacions en els assentaments i en el territori durant l’antiguitat tardana. Cota zero: revista d’arqueologia i ciència 2005; 87–98. 10.1016/j.antiviral.2005.11.006

[61] Revilla V. Hábitat rural y territorio en el litoral oriental de “Hispania Citerior”: perspectivas de análisis In: Noguera JM, editor. Poblamiento rural romano en el sureste de Hispania: 15 años después: Actas de las II Jornadas sobre Poblamiento rural romano en el sureste de Hispania. Murcia: Editum; 2010 pp. 25–70.

[62] Prevosti M. Cronologia i poblament a l’àrea rural de Baetulo: Museu de Badalona, Ajuntament de Badalona. Badalona: Oikostau; 1981 10.1007/BF00263726

[63] Francès J. Evolució de les formes d’hàbitat a la franja central de la costa catalana durant el primer mil.lenni a.n.e. RAP. 2005; 59–78.

[64] Carlús X, Lara C, López J, Oliva M, Palomo A, Rodríguez A, et al El paraje arqueológico de Can Roqueta (Sabadell, Vallés Occidental): diacronía y tipología de las ocupaciones. Bolskan. 2002; 121–139.

[65] Junyent E, Rodríguez JI, Maya JL, González JR, López-Cachero J. Excavaciones (1981–1983) en el poblado de Carretelà (Aitona, Segrià, Lleida). Revista d’arqueologia de Ponent. 2001; 151–233.

[66] Maya JL, Cuesta F, López-Cachero FJ. Genó: Un poblado del Bronce Final en el Bajo Segre. Barcelona: Publicacions i Edicions de la Universitat de Barcelona; 1998.

[67] Albizuri S, Alonso N, López-Cachero FJ. Economia i canvi social a Catalunya durant l’edat del bronze i la primera edat del ferro” In: Valenzuela-Lamas S, Padrós N, Belarte MC, Sanmartí J, editors. Economia agropecuària i canvi social a partir de les restes bioarqueològiques El primer mil·lenni aC a la Mediterrània occidental Actes de la V Reunió Internacional d’Arqueologia de Calafell (Calafell, 16 al 18 d’abril de 2009). Barcelona: Àrea d’Arqueologia (Universitat de Barcelona), Institut Català d’Arqueologia Clàssica; 2011 pp. 11–36.

[68] López-Cachero FJ. Primeros ensayos urbanísticos en el NE peninsular: el ejemplo de Genó y los poblados de espacio central. Pyrenae. 1999;30: 69–89.

[69] Pons E. La Fonollera (Torroella de Montgrí, Girona): un poblado al aire libre del Bronce Final Servicio Técnico de Investigaciones Arqueológicas de la Excma. Diputación Provincial de Girona; 1977.

[70] Pons E. L’hàbitat a Catalunya durant el primer mil-lenni aC: els precedents de l’habitació consolidada. Cota zero. 1994; 9–18.

[71] Alonso N. De la llavor a la farina: els processos agrícoles protohistòrics a la Catalunya occidental. Lattes: Milieux et Sociétés en France Méditerranéenne: Archéologie et histoire; 1999.

[72] Gracia-Alonso F, Garcia-Rubert D. La primera fase del poblamiento protohistórico en el área sur de la desembocadura del Ebro. El poblado fortificado de Sant Jaume-Mas d’en Serra (Alcanar), Campañas 1997–1998. RAP. 1999; 131–155.

[73] Sanmartí J, Belarte MC, Santacana J, Asensio D, Noguera J. L’assentament del bronze final i primera edat del ferro del Barranc de Gàfols (Ginestar, Ribera d’Ebre). Treballs de l’Àrea d’Arqueologia de la Universitat de Barcelona. Barcelona: Departament de Prehistòria, Història Antiga i Arqueologia de la Universitat; 2000.

[74] Agustí B, Mercadal O. Rituals funeraris i antropologia entre el Neolític final i l’Edat del Bronze inicial en el marc català i els territoris veïns. Pirineus i Veins al 3r Mil·lenni AC: XII Col.loqui intern d’arqueologia de Puigcerdà (Puigcerdà 2000). Puigcerdà: Institut d’Estudis Ceretans; 2002 pp. 591–642.

[75] Sperber L. Crises dans l’approvisionnement du métal en Europe de l’Ouest à l’âge du Bronze: passage du bronze au fer Réunion des Musées Nationaux. L’Europe au temps d’Ulysse Dieux et héros de l’âge du Bronze. Réunion des Musées Nationaux 1999 pp. 48–51.

[76] Pernicka E, Lutz J, Stöllner T. Bronze Age Copper Produced at Mitterberg, Austria, and its Distribution. Archaeologia Austriaca. 2016;100: 19–55.

[77] Junyent E. Els origens del ferro a Catalunya. Revista d’arqueologia de Ponent. 1992; 21–35.

[78] Armada X-L, Garcia i Rubert D, Montero Ruiz I, Moreno Martínez I, Rafel i Fontanals N, Rovira Hortalà MC. Minería y metalurgia durante la primera edad del Hierro. Procesos de cambio en el sur de Catalunya. RAP. 2005; 133–150.

[79] Pons E, Francès J. L’hàbitat del Bronze final i de la Primera Edat del Ferro a la Catalunya litoral i prelitoral. Cypsela. 1998; 31–46.

[80] Riera S, Parra I. Palinología holocénica en el litoral mediterráneo peninsular. Ed. 35 de Serie informes. In: La Serna-Ramos, editor. Polen y esporas: contribución a su conocimiento. Ed. 35 de Serie informes. Tenerife: Servicio de Publicaciones—Universidad de La Laguna; 1994 pp. 423–429.

[81] Riera S, Nadal J. Systèmes d’exploitation et anthropisation du paysage méditerranéen du Néolithique Ancien à la Premiere Âge du Fer: le cas de la dépression du Penedès (NE de la Péninsule Ibérique). Éditions du Comité des travaux historiques et scientifiques In: Richard H, Magny M, Mordant C, editors. Éditions du Comité des travaux historiques et scientifiques. Documents Préhistoriques; 2007 Available: http://portalrecerca.csuc.cat/27478410

[82] Currás Domínguez A. Estudio sobre la evolución de paisajes mediterráneos continentales en Lleida y Guadalajara durante los últimos 3000 años a partir de las secuencias polínicas de Ivars, Somolinos y Cañamares. 2012 Available: http://diposit.ub.edu/dspace/handle/2445/42650.

[83] Castellano Aragonés A. La introducció del torn a la plana occidental catalana (segles VII-VI a.n.e.). Universitat de Lleida 2014.

[84] Alonso N, Junyent E, Lafuente Á, López J (López i M. Poder, símbolo y territorio: el caso de la fortaleza de Arbeca. Saguntum: Papeles del Laboratorio de Arqueología de Valencia 1998; 355–372.

[85] López-Cachero FJ. Aproximació a la societat durant el bronze final i la primera edat del ferro: el cas de la necròpoli de Can Piteu-Can Roqueta (Sabadell, Vallès occidental, Barcelona). Barcelona: Societat Catalana d’Arqueologia; 2006.

[86] Rafel-Fontanals N. Colgantes de bronce paleoibéricos en el N.E. de la Península Ibérica. Algunas reflexiones sobre las relaciones mediterráneas. Pyrenae. 1997; 99–117.

[87] Aubet ME. El comerç fenici i les comunitats del ferro a Catalunya. Laietania: Estudis d’historia i d’arqueología de Mataró i del Maresme 1993; 21–40.

[88] Graells R. Indicis d’emergència aristocràtica al registre funerari del nord-est peninsular: la tomba Agullana 184. RAP. 2004; 61–84.

[89] Moreno-Ferrero I, Garcia-Alonso F. L’impacte del fenomen comercial fenici a les terres del Sénia durant el primer ferro a partir de l’estudi quantitatiu de la ceràmica: dades del jaciment de Sant Jaume (Alcanar, Montsià) La circulació d’àmfores al Mediterrani occidental durant la Protohistòria (segles VIII-III aC): aspectes quantitatius i anàlisi de continguts: [II Reunió Internacional d’Arqueología de Calafell]. Barcelona: Departament de Prehistòria, Història Antiga i Arqueologia (Universitat de Barcelona); 2004 pp. 191–202. Available: https://dialnet.unirioja.es/servlet/articulo?codigo=2034583.

[90] Garcia-Rubert D, Garcia F, Moreno-Ferrero I, Mercadal O. L’assentament de la primera edat del ferro de Sant Jaume-Mas d’en Serrà (Alcanar, Montsià). Balanç de les campanyes d’intervenció realitzades entre els anys 1997 i 2003 Món ibèric: als Països Catalans XIII Col·loqui Internaci- onal d’Arqueologia de Puigcerdà Homenatge a Josep Barberà. Puigcerdà: Institut d’Estudis Ceretans; 2003 pp. 117–140.

[91] Asensio D, López D, Mestres J, Molist N, Ros A, Senabre MR. De la primera edat del ferro a l’ibèric antic: la formació de les societats complexes a la zona del Penedès In: Belarte MC, Sanmarti J, editors. De les comunitats locals als espais arcaics: la formació de les societats complexes a la costa del Mediterrani occidental. Barcelona: Universitat de Barcelona—Institut Català d’Arqueologia Clàssica; 2006 pp. 1–19.

[92] Belarte MC, Sanmartí J, editors. De les comunitats locals als estats arcaics: la formació de les societats complexes a la costa del Mediterrani occidental. Barcelona: Universitat de Barcelona, Departament de Prehistòria, Història antiga i Arqueologia—Institut d’Arqueologia Clàssica (ICAC); 2006.

[93] Vázquez MP, Medina Morales J, González Pérez JR, Rodríguez JI. El jaciment de la serra del Calvari (la Granja d’Escarp, el Segrià, Lleida): estat de la qüestió. Revista d’arqueologia de Ponent. 2006; 64–110.

[94] Ferrer C, Noguera J, Santacana J, Belarte MC, Sanmarti J, Asensio D. El poblament de les comarques del curs inferior de l’Ebre durant el Bronze Final i la Primera Edat del Ferro In: Rovira-Port J, editor. Models d’ocupació, transformació i explotació del territori entre el 1600 i el 500 ANE a la Catalunya meridional i zones limítrofes de la depressió de l’Ebre. Sant Feliu de Codines: Museu Nacional d’Art de Catalunya; 1996 pp. 301–318. Available: https://dialnet.unirioja.es/servlet/articulo?codigo=605107.

[95] Asensio D, Belarte MC, Noguera J. El poblament ibèric al curs inferior de l’ebre (ribera d’ebre i baix ebre) In: Martin-Ortega A, Plana R, editors. Territori politic i territori rural durant l’Edat del Ferro a la Mediterrània occidental. Ullastret: Museu d’Arqueologia de Catalunya; 2001 pp. 283–299.

[96] Ruiz-Rodríguez A, Molinos M. Los iberos: análisis arqueológico de un proceso histórico. Crítica; 1993.

[97] Junyent E, Garcés I, López-Melción JB, Lafuente A. Els Vilars (Arbeca, Les Garrigues): primera edat del ferro i època ibèrica a la plana occidental catalana. Laietania: Estudis d’historia i d’arqueología de Mataró i del Maresme 1993; 41–60.

[98] Junyent E, Pérez- Almoguera A. L’antiguitat, d’Iltirta a Ilerda | Pagès Editors. Lleida: Pagès Editors; 2003 10.1016/s0025-7753(03)73876-7

[99] Sanmarti J. La formació i desenvolupament de les societats ibèriques a Catalunya. Butlletí Arqueològic. 2001; 101–132.

[100] Asensio D, Morer de Llorens J, Pou J, Santacana J, Sanmartí J. Evidències arqueològiques del procés d’emergència “d’élites” aristocràtiques a la ciutadella ibèrica d’Alorda Park (Calafell, Baix Penedès) In: Mercadal F, Barberá J, editors. Món ibèric als Països Catalans: XIII Col·loqui Internacional d’Arqueologia de Puigcerdà. Puigcerdà: Institut d’Estudis Ceretans; 2005 pp. 597–614.

[101] Graells R. ¿Culto heroíco durante la primera edad del Hierro e Ibérico antiguo en el noreste peninsular?: algunas consideraciones a partir del registro funerario. Cuadernos de prehistoria y arqueología. 2007; 91–115.

[102] Belarte MC, López Cachero FJ, Pons E, Rovira MC, Sanmartí J. From prestige objects to the productive revolution: iron and siderurgy in Catalonia during the first millennium BC In: Belarte MC, Rovira MC, Santmartí J, editors. Iron Metallurgy and the Formation of Complex Societies in the Western Mediterranean (1st Millennium BC) Proceedings of the 8th International Archaeological Meeting of Calafell (Calafell, from 6th to 8th October 2016). Barcelona: Àrea d’Arqueologia, Universitat de Barcelona; Institut Català d’Arqueologia Clàssica (ICAC); 2020 pp. 125–140. Available: https://recercat.cat//handle/2072/374098.

[103] Asensio D, Francès J, Pons E. Les implicacions econòmiques i socials de la concentració de reserves de cereals a la Catalunya costanera en època ibérica. Cypsela. 2002; 125–140.

[104] Sanmarti J. Demografía y cambio socio-cultural: el caso de la Iberia septentrional. Arqueología espacial. 2010; 91–108.

[105] Prats G, Antolín F, Alonso N. From the earliest farmers to the first urban centres: a socio-economic analysis of underground storage practices in north-eastern Iberia. Antiquity. 2020;94: 653–668. 10.15184/aqy.2019.153

[106] Fatás G. Apunt sobre els ilergets i llurs terres occidentals. Fonaments: prehistòria i món antic als Països Catalans. 1987; 11–22.

[107] Junyent E. L’evidència arqueològica en la definició de la societat estatal arcaica ilergeta In: Belarte MC, Dominique G, Sanmarti J, editors. Les estructures socials protohistòriques a la Gàl·lia i a Ibèria. Barcelona: Departament de Prehistòria, Història Antiga i Arqueologia (UB)—Institut Català d’Arqueologia Clàssica (ICAC); 2015 pp. 165–192.

[108] Olesti O. Integració i transformació de les comunitats ibèriques del Maresme durant el s. II-I a.C: un model de romanització per a la Catalunya litoral i prelitoral. Empúries. 2000; 55–86.

[109] Járrega R. El poblament rural i l’origen de les villae al nord-est d’Hispania durant l’època romana republicana (segles II-I aC). Quaderns de Preshistòria i Arqueologia de Castelló. 2000; 271–302.

[110] Martín A, Plana R. El territorio del oppidum de Ullastret (Girona) frente a la romanización. In: Oliveira V, editor. 3o Congresso de Arqueología Peninsular: UTAD. Vila Real: ADECAP; 2000. pp. 11–32.

[111] Castanyer P, Tremoleda J. La producció agrícola d’època romana al nord-est de Catalunya. Cota zero: revista d’arqueologia i ciència. 2005; 67–77.

[112] Arrayás I. Al voltant de la “romanització” del nord-est de la Península Ibérica. Reflexions sobre l’organització territorial i els fluxos comercials. Pyrenae. 2007; 47–72.

[113] Nolla J, Palahí L, Vivo J. De l’oppidum a la ciuitas.: La romanització inicial de la Indigècia. Girona: Universitat de Girona. Servei de Publicacions; 2010.

[114] Prevosti M. Els grans canvis del poblament a Catalunya, de la protohistòria a l’antiguitat. Butlletí de la Societat Catalana d’Estudis Històrics 2010; 45–46. 10.2436/20.1001.01.54.

[115] Aguilar Á, Picón P. Aproximación a la estructuración territorial en época romano- republicana y alto imperial en la comarca del Valles Occidental (Barcelona). Studia historica Historia antigua. 1989; 29–42.

[116] Aguilar Á. Avanç preliminar a I’estudi des cadastres romans a la comarca del Vallès. Barcelona: Publicaciones de la Universitat Autònoma de Barcelona; 1993.

[117] Plana R. Romanisation et aménagements fonciers dans le Nord-est catalan In: Doukellis P, Mendoni L, editors. Structures rurales et sociétés antiques. Besançon: Université de Franche-Comté; 1994 pp. 339–350.

[118] Burch J. L’ús de sitges en època republicana al nord-est de Catalunya. Revista d’arqueologia de Ponent. 1996; 207–216.

[119] Revilla V, Miret M. El poblament romá al litoral central de Catalunya. Quaderns de prehistòria i arqueologia de Castelló. 1995; 189–210.

[120] Escala O, Moya A, Tartera E, Vidal A. El jaciment de la Rosella (Tàrrega, Urgell): un camp de Sitges associat a un hàbitat de l’ibèric tardà (segles II i I a. de la n.e.). Urtx: Revista cultural de l’Urgell. 2011; 211–241.

[121] Padrós C, Belmonte C, Garcés I. Indicis d’un campament romà tardorepublicà en el Serrat dels Espinyers (Isona i Conca Dellà, Pallars Jussà), nova evidència anterior a la fundació d’Aeso. Pyrenae. 2016;47: 39–52.

[122] Garcés I, Belmonte C, Bermúdez X, Reyes T. Serrat dels Espinyers (Isona i Conca Dellà, Lleida, Catalonia), a multi-period storage site in the Pre-Pyrenees. Journal of Archaeological Science: Reports. 2020;30: 102173 10.1016/j.jasrep.2019.102173

[123] Revilla V. El poblamiento rural en el noreste de Hispania entre los siglos II a. C. y I d. C.: organización y dinámicas culturales y socioeconómicas Torres, atalayas y casas fortificadas: explotación y control del territorio en Hispania (s III a de C- s I d de C). Jaén: Universidad de Jaén; 2004 pp. 175–204.

[124] Remesal J, Revilla V, Martín-Arroyo DJ, Martín-Oliveras A, editors. Paisajes productivos y Redes comerciales En El Imperio Romano/ Productive Lands: 65. Barcelona: Publicacions i Edicions de la Universitat de Barcelona; 2019.

[125] Revilla V. Viticultura y actividades complementarias en el fundus: el ejemplo de la “Hispania Tarraconensis.” Latomus. 1999;58: 30–55.

[126] Macias JM, Fiz I, Lluís P, Miró MT, Guitart J, editors. Planimetria arqueològica de Tàrraco. Tarragona: Institut Català d’Arqueologia Clàssica (ICAC); 2007.

[127] Olesti O, Carreras C. New methods for the study of the social landscape from the Laietania wine production region of Northeastern Spain In: Funari PPA, Garraffoni RS, Letalien B, editors. New Perspectives on the Ancient World: Modern perceptions, ancient representations. Oxford, England: British Archaeological Reports Oxford Ltd; 2008 pp. 131–143.

[128] Martín-Oliveras A, Revilla V. The Economy of Laetanian Wine: A Conceptual Framework to Analyse an Intensive/Specialized Winegrowing Production System and Trade (First Century BC to Third Century AD). In: Verhagen P, Joyce J, Groenhuijzen MR, editors. Finding the Limits of the Limes: Modelling Demography, Economy and Transport on the Edge of the Roman Empire. Cham: Springer International Publishing; 2019 pp. 129–164. 10.1007/978-3-030-04576-0_8

[129] Revilla V. Territori, poblament i sistemes agraris al Penedès en època romana In: Esteve X, Molist N, Sabaté G, editors. Jornades d’Arqueologia del Penedès 2011. Vilafranca del Penedès (Barcelona): Institut d’Estudis Penedesencs; 2015 pp. 3–27.

[130] Miret M. El poblament d’època ibèrica i romana a la costa oriental de la Cossetània: la comarca del Garraf In: Prevosti M, Guitart J, Palet JM, editors. Territoris antics a la Mediterrània i a la Cossetània oriental: actes del Simposi Internacional d’Arqueologia del Baix Penedès. Barcelona: Generalitat de Catalunya, Departament de Cultura; 2003 pp. 363–376.

[131] Járrega R. L’Antiguitat tardana a les comarques de l’Alt Penedès, el Baix Penedès i el Garraf: estat actual dels coneixements In: Prevosti M, Guitart J, Palet JM, editors. Territoris antics a la Mediterrània i a la Cossetània oriental: actes del Simposi Internacional d’Arqueologia del Baix Penedès, El Vendrell, del 8 al 10 de novembre de 2001. Barcelona: Generalitat de Catalunya, Departament de Cultura; 2003 pp. 393–404.

[132] Revilla V. Territori, poblament i sistemes agraris al Penedès en època romana Esteve X, Miró C, Molist N, Sabaté G (Ed) Jornades d’Arqueologia del Penedès. Vilafranca del Penedès (Barcelona); 2011 pp. 3–27. Available: https://www.academia.edu/27365635/Territori_poblament_i_sistemes_agraris_al_Pened%C3%A8s_en_%C3%A8poca_romana.

[133] Gil I, Loriente A, Morán M, Payà X, Pérez-Almoguera A. De la Iltiŕta prerromana a la Ilerda tardorromana. Nuevos datos tras dos décadas de investigación continuada en Lérida. Archivo Español de Arqueología. 2001;74: 161–181. 10.3989/aespa.2001.v74.152

[134] Revilla V, Cela X. La transformación material e ideológica de una ciudad de Hispania: Iluro (Mataró) entre los siglos I y VII d.C. Archivo Español de Arqueología. 2006;79: 89–114.

[135] Pérez-Martínez M. Obsessa Terrachona marithimas urbes obtinuitThe aftermath of the Visigothic conquest of Tarraco by Euric according to the written sources and archaeology. Revista d’Arqueologia de Ponent. 2013; 237–248.

[136] Nolla JM. Girona romana: de la fundació a la fi del món antic. Girona: Publicacions de la diputació de Girona; 1987.

[137] Casas J. El Món rural d’època romana a Catalunya: (l’exemple del nord-est). Girona; 1995.

[138] Chavarría A. Establiments rurals del llevant de la Tarraconesa durant l’antiguitat tardana: transformacions arquitectòniques i funcionals, Els. Annals de l’Institut d’Estudis Gironins 1998;39: 9–30.

[139] Chavarría A. Poblamiento rural en el territorium de Tarraco durante la antigüedad tardía. Arqueología y Territorio Medieval. 2001;8: 55–76. 10.17561/aytm.v8i0.1673

[140] Chavarría A, Arce J, Brogiolo GP, editors. Villas tardoantiguas en el Mediterráneo Occidental. Madrid: Editorial CSIC—CSIC Press; 2006.

[141] Castanyer P, Tremoleda J, Dehesa R. De Vilauba a Villa Alba. L’hàbitat dels segles VI-VII dC de la vil·la romana de Vilauba (Camós, Pla de l’Estany). Tribuna d’ arqueologia. 2011;2010–2011: 9–21.

[142] Castanyer P, Tremoleda J, Colominas L, Antolín F. Després de les villæ. La transformació del camp al nord-est català en els segles VI i VII a partir de l’exemple de Vilauba / Villa Alba (Pla de l’Estany). Estudis d’història agrària. 2015; 43–65.

[143] López-Quiroga J. Arqueología del hábitat rural en la Península Ibérica (siglos V al X). Madrid: La Ergástula; 2009.

[144] Canal J, Canal E, Nolla JM, Sagrera J. La crisi de les “villae” i de la noblesa de la “Tarraconensis” en el canvi del segle V al VI: fonts textuals i evidències arqueològiques. Empúries. 2007; 185–197.

[145] Revilla V. Hàbitat rural, sistemes agraris i dinàmiques de la romanització a les Terres de l’Ebre. Miscel·lània del Centre d’Estudis de la Ribera d’Ebre 2018; 247–262.

[146] Coll JM, Roig J. La fi de les vil· les romanes baiximperials a la Depressió Prelitoral (segles IV-V): contextos estratigràfics i registre material per datar-los In: Fernández del Moral I, Menchon J, Vila JM, editors. Actes del IV Congrés d’Arqueologia Medieval i Moderna a Catalunya (2010). Tarragona: Associació Catalana per a la Recerca en Arqueologia Medieval (ACRAM)- Ajuntament de Tarragona; 2011 pp. 161–172.

[147] Chavarría A. El final de las Villae en Hispania (siglos IV-VII d.C.). Turnhout: Brepols Publishers; 2007.

[148] Revilla V. Producción agrícola, territorio y formas del hábitat en el NE de Hispania. Anales de Prehistoria y Arqueología. 2008; 311–329.

[149] Folch C, Gibert J, Martí R. Les explotacions rurals tardoantigues i altmedievals a la Catalunya Vella: una síntesi arqueològica | Estudis d’història agrària. Estudis d’història agrària. 2015.

[150] Carreté J-M, Keay S, Millett M. A Roman provincial capital and its hinterland: the survey of the territory of Tarragona, Spain, 1985–1990. Arbor A, editor. Cambridge, United Kingdom: Journal of Roman Archaeology; 1995.

[151] Artigues PL. La vil·la de Can Cabassa en els segles IV al VII. Arqueologia medieval: revista catalana d’arqueologia medieval. 2011; 8–23.

[152] Keay SJ. La importación de vino y aceite en la Tarraconense Oriental en la antigüedad El vi a l’Antiguitat: economia, producció i comerç al Mediterrani occidental. Badalona: Museu de Badalona; 1987 pp. 383–395.

[153] López-Cachero J, Pons E. La periodització del bronze final al ferro inicial a Catalunya. Cypsela. 2007; 51–64.

[154] Ringrose TJ. Bootstrapping and correspondence analysis in archaeology. Journal of Archaeological Science. 1992;19: 615–629. 10.1016/0305-4403(92)90032-X

[155] Lyman RL. Quantitative Paleozoology. Cambridge; New York: Cambridge University Press; 2008.

[156] Husson F, Lê S, Pagès J. Exploratory multivariate analysis by example using R. Chapman and Hall/CRC; 2017 Available: https://cran.r-project.org/web/packages/FactoMineR/index.html.

[157] Borg I, Groenen P. Modern multidimensional scaling: Theory and applications. Journal of Educational Measurement. 2003;40: 277–280.

[158] Llorente Rodríguez L, Quiralte V. A post-cranial osteometrical database for the Spanish ibex (Capra pyrenaica Schinz, 1838). Archaeofauna. 2016; 127–184. 10.13039/501100000780.

[159] Meadow RH. The use of size index scaling techniques for research on archaeozoological collections from the Middle East In: Becker C, Manhart H, Peters J, Schibler J, editors. Historia Animalium ex Ossibus Festschrift für Angela von den Driesch. Rahden/Westf: Verlag Marie Leidorf GmbH; 1999 pp. 285–300.

[160] Nieto-Espinet A. Element measure standard biometrical data from a cow dated to the Early Bronze Age (Minferri, Catalonia) [digital resource]. 2018 Available: Available from: https://www.researchgate.net/publication/326010953. 10.13140/RG.2.2.13512.78081.

[161] Davis SJM. Measurements of a Group of Adult Female Shetland Sheep Skeletons from a Single Flock: a Baseline for Zooarchaeologists. Journal of Archaeological Science. 1996;23: 593–612. 10.1006/jasc.1996.0056

[162] Albarella U, Payne S. Neolithic pigs from Durrington Walls, Wiltshire, England: a biometrical database. Journal of Archaeological Science. 2005;32: 589–599. 10.1016/j.jas.2004.11.008

[163] Driesch A von den. A Guide to the Measurement of Animal Bones from Archaeological Sites: As Developed by the Institut Für Palaeoanatomie, Domestikationsforschung und Geschichte Der Tiermedizin of the University of Munich. Harvard University Press; 1976.

[164] Hollander M, Wolfe DA, Chicken E. Nonparametric Statistical Methods. Edición: 3 Hoboken, New Jersey: Wiley; 2013.

[165] Harris M. Cannibales et monarques: essai sur l’origine des cultures. Flammarion; 1979.

[166] Harris M. Cultural Materialism: The Struggle for a Science of Culture. AltaMira Press; 2001.

[167] Bertoncello F, Fovet É, Tannier C, Gandini C, Lautier L, Nouvel P, et al Configurations spatiales et hiérarchiques du peuplement antique: des indicateurs quantitatifs pour une confrontation interrégionale. Variabilités environnementales, mutations sociales Nautre, intensités, échelles et temporalités des changements. 2011; 175–190.

[168] Ferguson B. Warfare, Culture, and Environment. Academic Press; 1984.

[169] Halstead P. Pastoralism or household herding? Problems of scale and specialization in early Greek animal husbandry. World Archaeology. 1996;28: 20–42. 10.1080/00438243.1996.9980329

[170] Boone JL. Subsistence strategies and early human population history: An evolutionary ecological perspective. World Archaeology. 2002;34: 6–25. 10.1080/00438240220134232 16475305

[171] Davis SJM. The Archaeology of Animals. New Haven Conn: Yale University Press; 1988.

[172] Eisenmann V. Sur quelques caractères adaptatifs du squelette d’Equus et leurs implications paléoécologiques. Bulletin du Muséum national d’histoire naturelle. 1984; 185–195.

[173] Rensis FD, Scaramuzzi RJ. Heat stress and seasonal effects on reproduction in the dairy cow—a review. Theriogenology. 2003;60: 1139–1151. 10.1016/s0093-691x(03)00126-2 12935853

[174] Connor SE, Vannière B, Colombaroli D, Anderson RS, Carrión JS, Ejarque A, et al Humans take control of fire-driven diversity changes in Mediterranean Iberia’s vegetation during the mid–late Holocene. The Holocene. 2019;29: 886–901. 10.1177/0959683619826652

[175] Palet JM, Julia R, Riera S, Ejarque A, Orengo HA, Miras Y, et al. Landscape Systems and Human Land-Use Interactions in Mediterranean Highlands and Littoral Plains during the Late Holocene: Integrated Analysis from the InterAmbAr Project (North-Eastern Catalonia). Bebermeier W, Hebenstreit R, Kaiser E, Krause J, editors. LandscapeArchaeology Proceedings of the International Conference. 2012;Special volume 3: 305–310.

[176] Riera S. Canvis ambientals i modelació antròpica del territori entre l’època ibèrica i l’altmedieval a Catalunya: aportacions de la palinologia. Cota zero: revista d’arqueologia i ciència. 2005; 99–107–107.

[177] Julià R, Montaner J, Ejarque A, Riera S, Castanyer P. Cambios del paisaje fluvio-estuarino del entorno de Empúries durante el Holoceno. Seminario: Paleopaisajes del litoral mediterráneo ibérico entre los cabos de Creus y Gata. Cartagena, Spain; 2016 Available: https://hal.archives-ouvertes.fr/hal-01879996.

[178] Riera S, Currás A, Palet J-M, Ejarque A, Orengo H, Miras Y. Variabilité climatique, occupation du sol et paysage en Espagne de l’Age du fer à l’époque médiévale: intégration des données paléoenvironnementales et de l’archéologie du paysage In: Hermon E, editor. Société et climats dans l’Empire romaine: pour une perspective historique et systémique de la gestion des ressources en eau dans l’Empire romain. Napoli: Editoriale scientifica; 2009 pp. 251–280.

[179] Orengo HA, Ejarque A, Albiach R. Water management and land-use practices from the Iron-Age to the Roman period in Eastern Iberia. Journal of Archaeological Science. 2014;49: 265–275. 10.1016/j.jas.2014.05.005.

[180] Roman-Ponce H, Thatcher WW, Caton D, Barron DH, Wilcox CJ. Thermal Stress Effects on Uterine Blood Flow in Dairy Cows. J Anim Sci. 1978;46: 175–180. 10.2527/jas1978.461175x 565348

[181] Hafez ESE, Hafez B. Reproduction in Farm Animals. John Wiley & Sons; 2013.

[182] García-Ispierto I, López-Gatius F, Bech-Sabat G, Santolaria P, Yániz JL, Nogareda C, et al Climate factors affecting conception rate of high producing dairy cows in northeastern Spain. Theriogenology. 2007;67: 1379–1385. 10.1016/j.theriogenology.2007.02.009 17412409

[183] Besso MA, Gardeisen A, Perrier X. Les restes fauniques du Rocher de l’Aigle à Nant (Aveyron). Documents d’archéologie méridionale. 2013;33: 235–247.

[184] Vincent C, Holder E. Synthèse bibliographique de dix ans d’étude du pâturage sur les landes du Cragou. Conseil Général du Finistère;Bretagne Vivante—SEPNB; 2008.

[185] Dupieux N. La gestion conservatoire des tourbières de France Premiers éléments scientifiques et techniques. Espaces Naturels de France; 1998 Available: https://www.decitre.fr/livres/la-gestion-conservatoire-des-tourbieres-de-france-9782951309807.html.

[186] Thulliez P, Magueur A. Guide technique d’aménagement et de gestion des zones humides du Finistère. Conseil général du Finistère, Forum des marais atlantiques Loire-Bretagne (France) 2012 Available: https://pole-lagunes.org/guide-technique-damenagement-et-de-gestion-des-zones-humides-du-finistere/.

[187] Rogosic J, Pfister JA, Provenza FD, Grbesa D. Sheep and goat preference for and nutritional value of Mediterranean maquis shrubs. Small Ruminant Research. 2006; 169–179. 10.1016/j.smallrumres.2005.04.01

[188] Ibáñez-Estévez JJ, Jiménez-Manchón S, Blaise É, Nieto-Espinet A, Valenzuela-Lamas S. Discriminating Management Strategies In Modern And Archaeological Domestic Caprines Using Low-Magnification And Confocal Dental Microwear Analyses. 2020 10.1016/j.quaint.2020.03.006

[189] Papachristou TG. Foraging behaviour of goats and sheep on Mediterranean kermes oak shrublands. Small Ruminant Research. 1997;24: 85–93. 10.1016/S0921-4488(96)00942-X

[190] Decandia M, Yiakoulaki M, Pinna G, Cabiddu A, Molle G. Foraging Behaviour and Intake of Goats Browsing on Mediterranean Shrublands In: Cannas A, Pulina G, Francesconi AHD, editors. Dairy Goats Feeding and Nutrition. CABI; 2008 pp. 161–188.

[191] King A. Diet in the Roman world: a regional inter-site comparison of the mammal bones. Journal of Roman Archaeology. 1999;12: 168–202. 10.1017/S1047759400017979

[192] Mackinnon M. Production and Consumption of Animals in Roman Italy: Integrating the Zooarchaeological and Textual Evidence. Portsmouth, R.I: Journal of Roman Archaeology; 2004.

[193] de Grossi Mazzorin J, Minniti C. Changes in lifestyle in ancient Rome (Italy) across the Iron Age/Roman transition In: Albarella U, Rizzetto M, Russ H, Vickers K, Viner-Daniels S, editors. The Oxford Handbook of Zooarchaeology. Oxford: Oxford University Press; 2017 pp. 128–146.

[194] Nieto-Espinet A, Trentacoste A, Guimarães S, Valenzuela-Lamas S. Cattle from the East, cattle from the West: diversity of cattle morphotypes in the Iberian Peninsula during late prehistoric and Roman times. In: Albarella U, Cleia D, Ginja C, Tereso J, Gabriel S, Pires AE, editors. Oxford: Oxbow Books; 2020.

[195] Nieto-Espinet A, Moya i Garra A, López-Melción J, Agusti B. Ofrenes o deixalles? El cas dels bovins (Bos taurus) en context funerari del jaciment del bronze ple de Minferri (Lleida, Catalunya). Monographies d’Archéologie Méditerranéenne. 2014;Hors-série: 53–112.

[196] Bökönyi S. History of domestic mammals in Central and Eastern Europe. 1974 [cited 15 Nov 2019]. Available: http://agris.fao.org/agris-search/search.do?recordID=US201300513105.

[197] Duval C. La taille du bétail est loin d’être un détail Lire les paysages économiques et culturels à travers la morphologie animale. Les nouvelles de l’archéologie 2017; 56–61. 10.4000/nda.3730

[198] López-Melción J, Gallart J. La societat a l’Edat del bronze In: Ribes-Foguet JL, editor. Sala d’Arqueologia Catàleg. Lleida: Institut d’Estudis Ilerdencs; 2002 pp. 119–134.

[199] Moya A, López-Melción JB, Lafuente A, Rey J, Tartera E, Vidal-Aixalà A. El Grup del Segre-Cinca II (1250–950 cal. a.n.e.) a les terres del Báix Cinca: el poblat clos de Vincament (Fraga, Osca). Revista d’arqueologia de Ponent. 2005; 13–58.

[200] Sanmarti J, Belarte MC, Santacana J, Asensio D, Noguera J. L’assentament Del Bronza Final I Primera Edat Del Ferro Del Barranc De Gàfols Barcelona: Universitat de Barcelona. Facultat de Geografía i Historia. Departament de Prehistòria, Història Antiga i Arqueología; 2000.

[201] Sardà S. Pràctiques de consum ritual al curs inferior de l’Ebre, Comensalitat, ideologia i canvi social (S.VII-VI ANE). TDX (Tesis Doctorals en Xarxa). Ph.D. Thesis, Universitat Rovira i Virgili. 2010. Available: http://www.tdx.cat/handle/10803/8637.

[202] Moret P. Poder, vaixella de luxe i cervesa: Polibi i els banquets dels reis ibèrics. Universitat Rovira i Virgili In: Diloli J, Sardà S, editors. Ideologia, pràctiques rituals i banquet al nord-est de la Península Ibèrica durant la Protohistòria. Universitat Rovira i Virgili. Tarragona: Arola Editors; 2009 pp. 239–252. Available: https://hal.archives-ouvertes.fr/hal-00723939.

[203] Sanmarti J, Belarte MC. Espais de culte i pràctiques rituals a la Catalunya protohistòrica. Quaderns de prehistòria i arqueologia de Castelló. 1997; 7–32.

[204] Sanmarti J, Asensio D, Belarte MC, Noguera J. Comerç colonial, comensalitat i canvi social a la protohistòria de Catalunya. Citerior Revista d"Arqueologia i Ciències de l’Antiguitat. 2009; 219–238.

[205] Dietler M. Feasts. Edición: 1 Tuscaloosa, Ala: The University of Alabama Press; 2010.

[206] Halstead P, Isaakidou V. A pig fed by hand is worth two in the bush:: Ethnoarchaeology of pig husbandry in Greece and its archaeological implications. Oxbow Books In: Albarella U, Trentacoste A, editors. Ethnozooarchaeology: The Present and Past of Human-Animal Relations. Oxbow Books 2011 pp. 160–174. 10.2307/j.ctvh1dwvg.20

[207] Wealleans AL. Such as pigs eat: the rise and fall of the pannage pig in the UK. Journal of the Science of Food and Agriculture. 2013;93: 2076–2083. 10.1002/jsfa.6145 23553313

[208] Rafel-Fontanals N. El conjunt arqueològic del Coll del Moro de Gandesa: algunes dades sobre el procés d’iberització a la zona In: Rovira-Port J, editor. Models d’ocupació, transformació i explotació del territori entre el 1600 i el 500 ANE a la Catalunya meridional i zones limítrofes de la depressió de l’Ebre. Barcelona: Museu Nacional d’Art de Catalunya; 1996 pp. 341–348.

[209] Asensio D, Belarte MC, Sanmartí J, Santacana J. Les meules rotatives du site ibérique d’Alcorda Park (Calafell, Baix Penedès, Tarragona). Pyrenae: Revista de Prehistòria i Antiguitat de la Mediterrània Occidental 2001; 57–73.

[210] Alonso N. Els molins rotatius: origen i expansió en la Mediterrània occidental. Revista d’Arqueologia de Ponent,. 1996; 183–198.

[211] Alonso N. «Moliendo en ibero, moliendo en griego»: aculturación y resistencia tecnológica en el Mediterráneo occidental durante la Edad del Hierro / “Milling in Iberian, milling in Greek”: acculturation and technological resistance in the Western Mediterranean. Vegueta: Anuario de la Facultad de Geografía e Historia 2015; 23–35.

[212] Rovira Hortalà MC. Aproximación a la agricultura protohistórica del Noreste de la Península Ibérica mediante el utillaje metálico In: Buxó R, Pons E, editors. Els productes alimentaris d’origen vegetal a l’Edat del Ferro de l’Europa occidental: de la producció al consum; Actes del XXII Col.loqui international per a l’Estudi de l’Edat del Ferro (Girona, 21–24 de Maig de 1998). Girona: Departament de Cultura, Generalitat de Catalunya; 2000 pp. 269–280.

[213] Burjachs F, Blech M, Marzoli D, Julià R. Evolución del uso del paisaje en relación con el uso del territorio en la Edad del Hierro en el NE de la península iberica In: Buxó R, Pons E, editors. productes alimentaris d’origen vegetal a l’edat del ferro de l’Europa Occidental: de la producció al consum Vol I: Actes del XXII Col·loqui Internac. Girona: Departament de Cultura, Generalitat de Catalunya; 1999 pp. 31–42.

[214] Piqué R. Paisatge i explotació forestal durant el I mil.lenni a.n.e. a la plana empordanesa. Cypsela. 2002; 211–228.

[215] Piqué R, Vila S, Alonso N. Changes in vegetation and fuel use from the Neolithic to the Middle Ages in the western Catalan plain. Saguntum: Papeles del Laboratorio de Arqueología de Valencia 2012; 85–95.

[216] Wolf ER. Los campesinos. Barcelona: Edit. Labor Sa; 1992.

[217] Picazo M, Curià E, McGlade J, Buxó R. Continuidad y transformación del paisaje: mil años de ocupación humana del Empordà. Revista d’arqueologia de Ponent. 1999; 7–28.

[218] Garcia D. Économie et réseau urbain protohistorique dans le Nord-Est du monde ibérique (Roussillon et Languedoc occidental) (VI-IIe s. av. J.-C.). Saguntum: Papeles del Laboratorio de Arqueología de Valencia 2000; 69–80.

[219] Burch J, Castanyer P, Nolla JM, Tremoleda J. Temps de canvis La romanització del nord-est de Catalunya. Time of changes: in the beginning of the romanization, 2010, ISBN, págs 89–108. 2010; 89–108.

[220] Olesti O. Urbanització, integració i gestió del territori al nord-est de la península Ibèrica en època republicana (segles II-I aC). Times of changes In the begining of the Romanisation. 2010; 11–59.

[221] Colominas L, Schlumbaum A, Saña M. The impact of the Roman Empire on animal husbandry practices: study of the changes in cattle morphology in the north-east of the Iberian Peninsula through osteometric and ancient DNA analyses. Archaeol Anthropol Sci. 2014;6: 1–16. 10.1007/s12520-013-0116-9

[222] Calvo VR. El poblamiento rural en el noreste de Hispania entre los siglos II a. C. y I d. C.: organización y dinámicas culturales y socioeconómicas Torres, atalayas y casas fortificadas: explotación y control del territorio en Hispania (s III a de C- s I d de C), 2004, ISBN 84-8439-212-0, págs 175–204. Universidad de Jaén; 2004 pp. 175–204. Available: https://dialnet.unirioja.es/servlet/articulo?codigo=1185354.

[223] Riera S, Palet J-M. Evolució del sector de Montjuïc-el Port entre l’època romana i almedieval (s. III–X): una contribució a l’estudi diacrònic del paisatge. In: Mestre J, editor. II Congrés d’història de Barcelona La ciutat i el seu territorio, dos mil anys d’història, Barcelona,. Institut Ramon Muntaner; 1993. pp. 49–70.

[224] Allen M, Sykes N. New animals, new landscapes and new worldviews: the Iron Age to Roman transition at Fishbourne. Sussex Archaeological Collections. 2011;149: 7–24. 10.5284/1000334

[225] Albarella U, Valenzuela-Lamas S. Animal Husbandry in the Western Roman Empire: A Zooarchaeological Perspective. Cambridge: Cambridge University Press; 2017.

[226] Colominas L. Arqueozoología y Romanización: Producción, distribución y consumo de animales en el nordeste de la Península Ibérica entre los siglos V ane-V dne. Edición: Bilingual. Oxford, England: British Archaeological Reports Oxford Ltd; 2013.

[227] Padrós N, Valenzuela-Lamas S. La Llosa i els Antigons, una aproximació a la producció ramadera de les villae de l’ager Tarraconensis. Segles III-VI dC: La Llosa and Els Antigons, an approach to stockbreeding in the villae of the ager Tarraconensis. 3rd - 6th centuries AD In: Prevosti M, Guitart J, editors. Ager Tarraconensis (Aspectes històrics i marc natural). Tarragona: Institut Català d’Arqueologia Clàssica (ICAC); 2010 pp. 200–206.

[228] Valenzuela-Lamas S. Paisatge, alimentació i gestió dels ramats als Antigons a partir de les restes de fauna (vertebrats i mol·luscos) In: Prevosti M, Guitart J, editors. Ager Tarraconensis (Aspectes històrics i marc natural). Institut Català d’Arqueologia Clàssica (ICAC); 2010 pp. 181–191.

[229] Colominas L, Saña M. Animal husbandry in the North-East of Catalonia from the 1ST to the 5TH Century AD: improvement and importation. Studies on the Rural World in the Roman Period. 2009.

[230] Minniti C, Valenzuela-Lamas S, Evans JA, Albarella U. Widening the market. Strontium isotope analysis on cattle teeth from Owslebury (Hampshire, UK) highlights changes in livestock supply between the Iron Age and the Roman period. Journal of Archaeological Science. 2014;42: 305–314. 10.1016/j.jas.2013.10.008

[231] Madgwick R, Lewis J, Grimes V, Guest P. On the hoof: exploring the supply of animals to the Roman legionary fortress at Caerleon using strontium (87Sr/86Sr) isotope analysis. Archaeol Anthropol Sci. 2019;11: 223–235. 10.1007/s12520-017-0539-9

[232] Ripoll G, Arce J. Transformación y final de las villae en occidente (siglos IV-VIII): problemas y perspectivas. Arqueología y Territorio Medieval. 2001;8: 21–54. 10.17561/aytm.v8i0.1672

[233] Leveau P, Provansal M, Bruneton H, Palet J-M, Poupet P, Walsh K. La Crise environnementale de la fin de l’Antiquité et du Haut moyen âge: Définition d’un modèle et retour aux milieux réels. Annales Littéraires In: Richard H, Vignot A, editors. Équilibres et ruptures dans les écosystèmes durant les 20 derniers millénaires en Europe de l’Ouest, Actes du colloque international de Besançon. Annales Littéraires. Presses universitaires de Franche-Comtoises; 2002 pp. 291–303.

