# Supplementary material for: Resilience and livestock adaptations to demographic growth and technological change: A diachronic perspective from the Late Bronze Age to Late Antiquity in NE Iberia
Source: PLoS One. 2021 Feb 17;16(2):e0246201. doi: 10.1371/journal.pone.0246201 (PMC7888671; doi:10.1371/journal.pone.0246201)
Supplement: S1 Checklist — (DOC) [file pone.0246201.s001.doc]

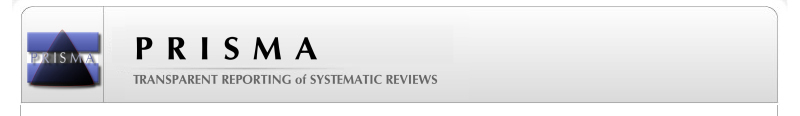
**PRISMA 2009 Flow Diagram**

**Screening**

**Included**

**Eligibility**

**Identification**

Records identified through database searching
(n = 101 sites )

Additional records identified through other sources
(n = 0 )

Records after duplicates removed
(n = 101 sites )

Records screened
(n = 101 sites )

Records excluded
(n = 16 sites )

Full-text articles assessed for eligibility
(n = 77 )

Full-text articles excluded, with reasons
(n = 0 )

Studies included in qualitative synthesis
(n = 85 sites )

Studies included in quantitative synthesis (meta-analysis)
(n = 85 sites )
