# Supplementary material for: Resilience and livestock adaptations to demographic growth and technological change: A diachronic perspective from the Late Bronze Age to Late Antiquity in NE Iberia
Source: PLoS One. 2021 Feb 17;16(2):e0246201. doi: 10.1371/journal.pone.0246201 (PMC7888671; doi:10.1371/journal.pone.0246201)
Supplement: S1 File — (PDF) [file pone.0246201.s002.pdf]

Table of data set for the sites cited in the text

ZooMWest ERC-StG project

Zone	Period	Site names	num	Type of site	Ab	NISP	References
OP	LBA	**Carretelà**	16	Closed settlement	CS	393	Albizuri & Colomer, 2001-2002
OP	LBA	**Vilot de Montagut 0-III**	63	Open settlement	OS	324	Gómez, 2000
OP	LBA	**Vincament**	64	Closed settlement	CS	106	Nieto, 2002
OP	LBA	**Zafranales**	65	Closed settlement	CS	532	Castaños, 1988
OP	LBA	**Cova Punta Farisa**	18	Shelter	S	176	Albizuri & Nadal, 1993
CC	LBA	**Can Roqueta CRV**	15a	Scattered village with silos	SVS	316	Albizuri, 2008
CC	LBA	**Can Roqueta TR**	15b	Scattered village with silos	SVS	310	Piña & Saña, 2004
CC	LBA	**Can Gambús 3**	14a	Scattered village with silos	SVS	106	Orri, 2005-2006
NC	LBA	**Sant Martí Empúries**	43a	Scattered village	SV	137	Casellas, 1999a
NC	LBA	**Fonollera**	21	Scattered village	SV	1315	Estévez,1997; Colomer, 1989; Albizuri, 2018
OP	EIA	**La Pedrera IV-VII**	37	Fortified settlement	FS	343	Miró, 1989a
OP	EIA	**Tossal Molinet I-II**	55a	Open settlement	OS	560	Gómez, 2000
OP	EIA	**Vilars 0-I**	59a	Fortified settlement	FS	627	Miró, 1992; Nieto Espinet, 2012
SC	EIA	**Puig de la Misericordia**	39	Fortified settlement	FS	854	Castaños, 1994
SC	EIA	**Puig de la Nau**	40a	Fortified settlement	FS	321	Castaños,1995
SC	EIA	**Barranc de Gàfols_1**	6a	Scattered village	SV	166	Albizuri & Nadal, 1999
SC	EIA	**Sant Jaume Mas d'en Serra_sect. 1**	42	Aristocratic residence	AR	394	Font, 2016
SC	EIA	**Barranc de Gàfols_2**	6b	Open settlement	OS	1101	Albizuri & Nadal, 1999
CC	EIA	**Sitges UAB**	48	Scattered village with silos	SVS	98	Albizuri et al.,1985; Albizuri et al., 2010
CC	EIA	**Can Roqueta_DIASA**	15c	Scattered village with silos	SVS	307	Casellas et al. 1999
CC	EIA	**Turo Font de la Canya 0**	57a	Silo field	SF	208	Valenzuela Lamas, 2008
CC	EIA	**Mas d en Boixos**	90a	Silo field	SF	*	Nieto Espinet et al. 2019
NC	EIA	**Sant Martí Empúries**	43b	Silo field	SF	1073	Casellas, 1999a; Albizuri, 2018
OP	MIA1	**Tossal Molinet III**	55b	Open settlement	OS	220	Gómez, 2000
OP	MIA1	**Vilars II**	59b	Fortified settlement	FS	2968	Miró, 1992; Nieto Espinet, 2012
OP	MIA1	**Molí Espígol**	33a	Urban	U	*	Valenzuela Lamas & Nieto Espinet, 2020a
SC	MIA1	**Puig de la Nau**	40b	Fortified settlement	FS	3106	Castaños, 1995
CC	MIA1	**Can Gambús 3**	14b	Silo field	SF	168	Orri, 2005-2006
CC	MIA1	**Alorda Park 2a**	2a	Aristocratic residence	AR	509	Valenzuela Lamas, 2008
CC	MIA1	**Turó Font de la Canya 1**	57b	Silo field	SF	232	Valenzuela Lamas, 2008
CC	MIA1	**Ca n'Oliver 1**	9a	Fortified settlement	FS	332	Albizuri, 1990; Albizuri et al. 2010
CC	MIA1	**Torre Roja**	89a	Fortified settlement	FS	*	Valenzuela Lamas, 2008
NC	MIA1	**Sant Sebastià de la Guarda**	44a	Fortified settlement	FS	3997	Colominas, 2017
NC	MIA1	**Illa d'en Reixac 2-3**	24a	Urban	U	131	Casellas, 1999b
NC	MIA1	**Sant Martí Empúries**	43c	Open settlement	OS	338	Casellas, 1999a
CC	MIA1	**Mas d'en Boixos**	90b	Silo field	SF	*	Nieto Espinet et al. 2019
OP	MIA2	**Vilars III-IV**	59c	Fortified settlement	FS	110	Miró,1992; Nieto Espinet, 2012
OP	MIA2	**Molí d'Espígol**	33b	Urban	U	2280	Colominas, 2017
OP	MIA2	**Sigarra**	47a	Urban	U	563	Nieto Espinet, 2014
OP	MIA2	**Olius**	35	Silo field	SF	1535	Colominas et al., 2017b
SC	MIA2	**Moleta del Remei 1_2**	32	Fortified settlement	FS	1364	Albizuri & Nadal, 1991
SC	MIA2	**Coll del Moro**	71	Fortified settlement	FS	*	Font 2016
CC	MIA2	**Mas d'en Gual**	78	Rural settlement with silo field	RSSF	*	Valenzuela Lamas et al. 2010
CC	MIA2	**Can Rodon**	103a	Rural settlement with silo field	RSSF	*	Valenzuela-Lamas & Nieto Espinet, 2020
CC	MIA2	**Alorda Park 2b**	2b	Aristocratic residence	AR	692	Valenzuela Lamas, 2008
CC	MIA2	**Penya del Moro**	38	Fortified settlement	FS	1136	Miró & Molist, 1982
CC	MIA2	**Can Bartomeu**	10a	Silo field	SF	1061	Valenzuela Lamas & Nieto Espinet, 2019
CC	MIA2	**Olèrdola 1**	34a	Fortified settlement	FS	123	Valenzuela Lamas, 2008
CC	MIA2	**Torre Roja**	89b	Fortified settlement	FS	*	Valenzuela Lamas, 2008
CC	MIA2	**Puig Castellar**	25	Fortified settlement	FS	*	Nadal & Estrada, 2003
CC	MIA2	**Turó del Vent 1-2**	56	Silo field	SF	1814	Oliva,1998; Oliva, 2000
CC	MIA2	**Can Gambús 1**	14c	Silo field	SF	*	Molina Vallmitjana, 2006

CC	MIA2	**Turó Font de la Canya 2-3**	57c	Silo field	SF	586	Valenzuela Lamas, 2008
CC	MIA2	**Ca n Oliver 2-3**	9b	Fortified settlement	FS	1939	Albizuri, 1990; Albizuri et al. 2010
CC	MIA2	**Turó de la Rovira**	13	Fortified settlement	FS	932	Colominas, 2019
NC	MIA2	**Mas Castellar fortified**	29a	Fortified settlement	FS	2033	Colominas, 2013a
NC	MIA2	**Ciutadella de Roses**	17	Urban	U	180	Montero, 2000
NC	MIA2	**Gou Batlle**	22	Periurban	PU	639	Valenzuela Lamas et al., 2017
NC	MIA2	**Illa d'en Reixac 4-6**	24b	Urban	U	2467	Casellas, 1999b
NC	MIA2	**St. Julià de Ramis**	50a	Fortified settlement	FS	510	Colominas et al. 2017
NC	MIA2	**Bosc del Congost**	7a	Silo field	SF	108	Colominas & Saña, 2009a; Colominas & Saña, 2009b
NC	MIA2	**Saus**	46	Silo field	SF	434	Colominas et al. 2017
NC	MIA2	**Puig de Sant Andreu**	88	Urban	U	*	Valenzuela Lamas, 2017
NC	MIA2	**Mas Castellar rural**	29b	Rural settlement with silo field	RSSF	904	Colominas, 2013
NC	MIA2	**Esquerda**	1	Fortified settlement	FS	394	Buxó & Martí, 1994
OP	RR	**Sigarra**	47b	Urban	U	172	Nieto Espinet, 2014
OP	RR	**Rosella**	84	Rural settlement with silo field	RSSF	*	Nieto Espinet, 2013
OP	RR	**Puig Castellar Biosca**	87	Fortified settlement	FS	*	Valenzuela Lamas & Padrós, 2010
OP	RR	**Hereuet**	91	Rural settlement with silo field	RSSF	*	Valenzuela Lamas & Padrós, 2010
OP	RR	**Missatges**	92	Rural settlement with silo field	RSSF	*	Font, 2016
OP	RR	**Ilerda**	23a	Urban	U	*	Nieto Espinet & Valenzuela Lamas, 2020b
SC	RR	**Torre Cremada**	53	Fortified settlement	FS	110	Lignereux & Périn, 2006
CC	RR	**Can Mateu**	101	Urban	U	128	Nieto Espinet & Valenzuela-Lamas, 2019
CC	RR	**Can Benet**	102	Urban	U	95	Nieto Espinet & Valenzuela-Lamas, 2019
CC	RR	**Can Bartomeu**	10b	Silo field	SF	462	Valenzuela Lamas & Nieto Espinet, 2019
CC	RR	**Vilarenc**	58a	Villa	V	*	Valenzuela Lamas, 2012
CC	RR	**Burriac**	8	Urban	U	264	Albizuri & Colomer, 1998
CC	RR	**Can Feu**	12b	Silo field	SF	1177	Mañosa, 1990
CC	RR	**Can Roqueta TR**	15d	Silo field	SF	803	Piña & Saña, 2004
CC	RR	**Olèrdola 2**	34b	Fortified settlement	FS	318	Valenzuela Lamas, 2008
CC	RR	**Torre Roja**	89c	Fortified settlement	FS	*	Valenzuela Lamas, 2008
CC	RR	**Sant Boi_Pl. Constitució**	41b	Villa	V	370	Molinas Amorós, 2006
NC	RR	**Mas Castellar**	29c	Silo field	SF	1264	Colominas, 2017
NC	RR	**St. Julià de Ramis**	50b	Fortified settlement	FS	495	Colominas, 2017
NC	RR	**Sant Sebastià de la Guarda**	44b	Fortified settlement	FS	1043	Colominas et al. 2017
NC	RR	**Mas Gusó**	30	Villa	V	889	Colominas et al. 2017
NC	RR	**Olivet d'en Pujol**	36	Villa	V	1017	Colominas , 2013b
NC	RR	**Bosc del Congost**	7b	Silo field	SF	621	Colominas & Saña, 2009a; Colominas & Saña, 2009b
OP	ERE	**Iesso**	94	Urban	U	*	Valenzuela-Lamas & Padrós, 2010
OP	ERE	**Villa Torre Andreu**	95	Villa	V	*	Casellas, 1993
OP	ERE	**Ilerda_ C/ Magdalena 47**	23c	Urban	U	442	Casellas, 1990a; Casellas ,1990b
OP	ERE	**Ilerda_ C/ Bafart 46**	23d	Urban	U	143	Casellas, 1990a; Casellas, 1990b
CC	ERE	**Villa Vinyet**	61a	Villa	V	540	Colominas et al., 2017
CC	ERE	**Vilarenc**	58b	Villa	V	153	Valenzuela Lamas, 2012
CC	ERE	**Baetulo**	5	Urban	U	1207	Colominas, 2013a
CC	ERE	**Can Sant Joan**	106	Silo field	SF	*	Molina Vallmitjana, 2008-2009
CC	ERE	**Can Rodon**	103b	Villa	V	146	Nieto Espinet & Valenzuela Lamas, 2019b
CC	ERE	**Can Feu**	12a	Villa	V	130	Mañosa, 1990
CC	ERE	**La Llosa**	27a	Villa	V	334	Padrós & Valenzuela Lamas, 2010; Valenzuela Lamas, 2010a
CC	ERE	**Antigons- wast dump**	3a	Villa	V	537	Valenzuela Lamas, 2010a
CC	ERE	**Torre Llauder**	54a	Villa	V	363	Valenzuela Lamas & Nieto Espinet, 2020b
CC	ERE	**Sant Boi_Pl. Constitució**	41c	Villa	V	1499	Molinas Amorós, 2006
CC	ERE	**Can Cruzate**	11b	Urban	U	*	Valenzuela Lamas & Nieto Espinet, 2020a
NC	ERE	**Vilauba**	60a	Villa	V	1907	Colominas, 2013a; Colominas et al. 2017
NC	ERE	**Tolegassos**	52	Villa	V	6488	Casas & Soler Fusté, 2003; Colominas et al., 2017
NC	ERE	**Empúries**	19b	Urban	U	2235	Buxó et al., 2007; Colominas, 2017
OP	LRE	**Ilerda**	23b	Urban	U	105	Casellas, 1990a; Casellas, 1990b
CC	LRE	**Can Cruzate**	11a	Urban	U	289	Valenzuela Lamas & Nieto Espinet 2020a

CC	LRE	**Tarraco**	51	Urban	U	345	Miró, 1989b
CC	LRE	**Villa Vinyet**	61b	Villa	V	540	Colominas et al. 2017
CC	LRE	**Antigons_*Nymphaeum***	3b	Villa	V	108	Valenzuela Lamas, 2010
CC	LRE	**Torre Llauder**	54b	Villa	V	1077	Valenzuela Lamas & Nieto Espinet, 2020b
CC	LRE	**La Llosa**	27b	Rural settlement	RS	281	Padrós & Valenzuela Lamas, 2010; Valenzuela Lamas, 2010
CC	LRE	**Sant Boi_Pl. Constitució**	41d	Rural settlement with silo field	RSSF	371	Molinas Amorós, 2006
NC	LRE	**Villa dels Ametllers**	62	Villa	V	251	Colominas & Saña, 2009b
NC	LRE	**Vilauba**	60b	Villa	V	638	Colominas, 2013a; Colominas et al., 2017
OP	LA	**Vilans de Reig**	97	Rural settlement with silo field	RSSF	*	Nieto Espinet & Escala, 2004
CC	LA	**Iluro_VI_VII**	26	Urban	U	1569	Orri & Estrada, 2004
CC	LA	**Mallols**	28	Rural settlement with silo field	RSSF	1684	Nadal & Estrada, 2007b
CC	LA	**Santa Margarida**	45	Ecclesiastical	E	860	Valenzuela Lamas & Navarro, 2007
CC	LA	**Solana**	49	Rural settlement with silo field	RSSF	1400	Estrada & Nadal, 2007
CC	LA	**Can Gambús 1**	14d	Rural settlement with silo field	RSSF	8125	Molina Vallmitjana, 2006
CC	LA	**Torre Llauder**	54c	Rural settlement	RS	1992	Valenzuela Lamas & Nieto Espinet, 2020b
NC	LA	**Sant Martí Empúries**	43c	Rural settlement	RS	*	Valenzuela Lamas & Nieto Espinet, 2018
NC	LA	**Aubert**	4	Rural settlement with silo field	RSSF	195	Folch & Gibert, 2014
NC	LA	**Empúries**	43d	Rural settlement	RS	223	Colominas et al. 2017
NC	LA	**Vilauba**	60c	Rural settlement	RS	718	Colominas, 2013a; Colominas et al. 2017

** The sites that have empty NISP cells correspond to those for which we only have biometric data*

Zones: Occcidental Plaine (OP), South Coast (SC), Central Coast (CC), North Coast (NC).
Periods: Late Bronze Age (LBA), Early Iron Age (EIA), Middle Iron Age 1 (MIA1), Middle Iron Age 2 (MIA2), Roman Republic (RR), Early Roman Empire (ERE), Late Roman Empire (LRE), Late Antiquity (LA)

References

ALBIZURI, S. (1990). *Paleoeconomía y ritualidad. Análisis zooarqueológico del yacimiento ibérico "Turó de Ca n'Olivé" (Cerdanyola, Vallès Occidental).* Licence Thesi. Departament de Prehistòria, Història Antiga i Arqueologia: Universitat de Barcelona.

ALBIZURI, S. (2008). "Informe de l'estudi arqueozoològic de Can Roqueta / Can Revella 2008 (Sabadell i Barberà del Vallès, Vallès Occidental)". In TERRATS, N.; OLIVA, M. (2009). *El jaciment arqueològic de Can Roqueta-Can Revella (Sabadell i Barberà del Vallès, Vallès Occidental) 2005/2006*. Vol. II. Barcelona: Departament de Cultura, Servei d'Arqueologia: Generalitat de Catalunya. Estudi complementari. Faunal report.

ALBIZURI, S. (2018). "Noves dades sobre la ramaderia a les societats camperoles del bronze final i la primera edat del ferro en les depressions de l'Empordà i la Selva". *Cypsela*. Girona: Museu d'Arqueologia de Catalunya, núm. 21, p. 97-118.

ALBIZURI, S.; COLOMER, S.; RAMADA, X. (1985). "Estudio de la fauna de los silos de la UAB". *Estudios de la Antigüedad*. Barcelona: Universitat Autònoma de Barcelona, núm. 2, p. 219-229.

ALBIZURI, S.; NADAL, J. (1991). *Informe de l'estudi faunístic del jaciment de la Moleta del Remei (Alcanar, Montsià)*. Barcelona: Departament d'Història i Arqueologia, Secció de Prehistòria i Arqueologia, Universitat de Barcelona. Faunal report, cited in Font (2016).

ALBIZURI, S.; NADAL, J. (1993). "Análisis faunístico del yacimiento de Punta Farisa (Fraga, Huesca)". *Estudios de la Antigüedad*. Barcelona: Universitat Autònoma de Barcelona, núm. 6/7, p. 31-37.

ALBIZURI, S.; COLOMER, S. (1998). "Annex: Anàlisis de les restes faunístiques". In MIRÓ, J., PUJOL, J., GARCIA, J. "El dipòsit del sector occidental del poblat ibèric de Búrriac (Cabrera de Mar. El Maresme). Una aportació al coneixement de l'època ibèrica tardana al Maresme (s. I a. C.)". *Laietània*. Mataró: Museu de Mataró, núm. 4, p. 159-180.

ALBIZURI, S.; NADAL, J. (1999a). "Aprovechamiento y producción animal en época ibérica. Consideraciones generales económicas". *Limes: Revista de Arqueología*. Cerdanyola: Col·lectiu de recerques arqueològiques de Cerdanyola, núm. 6-7, p. 40-51.

ALBIZURI, S.; NADAL, J. (1999). "El Barranc de Gàfols (Ginestar, Tarragona) y Aldovesta (Benifallet, Tarragona): El Estudio Arqueozoológico como base de teorización sobre la dieta humana a principios de la edad del hierro y la complejidad económica en el curso bajo del Ebro". *Pyrenae*. Barcelona: Departament d'Història i Arqueologia, Secció de Prehistòria i Arqueologia, Universitat de Barcelona, núm. 30, p. 207-221.

ALBIZURI, S.; COLOMER, S. (2001-2002). "Informe arqueozoológico de Carretelà (Aitona, Segrià, Lleida)". *Revista d'Arqueologia de Ponent*. Lleida: Departament d'Història, Universitat de Lleida, núm. 11-12, p. 235-245.

ALBIZURI, S.; NIETO ESPINET, A.; VALENZUELA LAMAS, S. (2010). "Canvis en l'alimentació càrnia a Catalunya entre els segles XII i III aC". In MATA, C.; PÉREZ JORDÀ; G.; VIVES-FERRÁNDIZ, J. (ed.). *De la cuina a la taula.* IV reunió d'economia en el primer mil·lenni aC celebrada a Caudete de las Fuentes el 22-23 d'octubre del 2009. València: Departament de Prehistòria i Arqueologia, Universitat de València, p. 161-170. (SAGVNTVM: Papeles del laboratorio de arqueología de Valencia; Extra-9).

BUXÓ, R.; MARTÍ, J. (1994). “La fauna”. In OLLICH, I.; ROCAFIGUERA, M. (ed.). *L'oppidum ibèric de l'Esquerda, campanyes 1981-1991 (Les Masies de Roda de Ter, Osona)*. Barcelona: Departament de Cultura, Servei d'Arqueologia: Generalitat de Catalunya, p. 65–68. (Memòries d'Intervencions Arqueològiques a Catalunya; 7).

BUXÓ, R.; COLOMINAS, L.; SAÑA, M. (2007). “Les espècies de fauna d'Empúries documentades a través de l'arqueozoologia: Palaiapolis, Neapolis i ciutat romana”. In AQUILUÉ, X. (ed.). *Animals d'Empúries. La fauna i l'home a l'antiguitat*. L'Escala: Museu d'Arqueologia de Catalunya-Empúries, p. 38-42.

CASAS, J.; SOLER, V. (2003). *La villa de Tolegassos. Una explotación agrícola de época romana en el territorio de Ampurias*. Oxford: Hadrian Books. (BAR International Series; 1101).

CASELLAS, S. (1990a). *Anàlisi de les restes faunístiques de la intervenció 46 (Carrer Bafart) de la ciutat de Lleida.* Lleida: Servei d'Arqueologia (Ajuntament de Lleida), Departament d'Història (Universitat de Lleida). Faunal report.

CASELLAS, S. (1990b). *Anàlisi de les restes faunístiques de la intervenció 47 (c/ costa de Magdalena) de la ciutat de Lleida.* Lleida: Servei d'Arqueologia (Ajuntament de Lleida), Departament d'Història (Universitat de Lleida). Faunal report.

CASELLAS, S. (1993). "Restes faunístiques". In Pérez, A., Rafel, N. (dir.). *La vil·la romana de Torre Andreu (La Bordeta, Lleida). Un establiment suburbà dels segles UU-III dC*. Monografies d'Arqueologia Urbana, núm. 5, p. 91-97.

CASELLAS, S. (1999a). “Les restes de fauna”. In AQUILUÉ, X.; SANTOS, M.; ALCALDE, G. (ed.). *Intervencions arqueològiques a Sant Martí d'Empúries (1994-1996). De l'assentament precolonial a l'Empúries actual*. Girona: Museu d'Arqueologia de Catalunya-Empúries, Generalitat de Catalunya, p. 636-638. (Monografies Emporitanes; 9).

CASELLAS, S. (1999b). “Els Macromamífers”. In MARTÍN, A.; BUXÓ, R.; LÓPEZ MELCIÓN, J.; MATARÓ, M. (dir.). *Excavacions arqueològiques a l'Illa d'en Reixac (1987-1992)*. Girona: Museu d'Arqueologia de Catalunya-Ullastret, p. 299-303. (Monografies d'Ullastret; 1).

CASELLAS, S.; MONTÓN, S.; MARTÍNEZ, J. (1999). “Les restes faunístiques de Can Roqueta. Campanyes de 1990 i 1992”. In GONZÀLEZ MARCÉN, P.; MARTÍN, A.; MORA, R. (ed.) *Can Roqueta. Un establiment pagès prehistòric i medieval (Sabadell, Vallès Occidental)*. Barcelona: Direcció General del Patrimoni Cultural, Servei d'Arqueologia, p. 255-258. (Excavacions arqueològiques a Catalunya; 16).

CASTAÑOS, P. (1988). “Estudio de los restos faunísticos del yacimiento de Zafranales”. In MONTÓN, F. (ed.). *Zafranales, un asentamiento musulmán y un hábitat del Bronce*, *Annales*. Barbastro-Zaragoza: Universidad Nacional de Educación a Distancia, p. 147-165. (Anuario del Centro de la Universidad Nacional de Educación a Distancia; 5).

CASTAÑOS, P. (1994). “Estudio de los restos óseos”. In OLIVER, A. (ed.). *El poblado ibérico del Puig de la Misericòrdia de Vinaròs*. Vinaròs: Associació Cultural Amics de Vinaròs, p. 155-186.

CASTAÑOS, P. (1995). “Análisis faunístico”. In OLIVER, A.; GUSI, F. (ed.). *El Puig de la Nau: un hábitat fortificado ibérico en el ámbito mediterráneo peninsular.* Castelló: Diputació Provincial de Castelló. Servei d'Investigacions Arqueològiques i Prehistòriques, p. 307-336.

COLOMER, M. À. (1989). *Estudi arqueozoològic del jaciment del bronze final de la Fonollera (Torroella del Montgrí, Baix Empord*à). Treball de recerca. Barcelona: Departament de la Universitat Autònoma de Barcelona.

COLOMINAS, L. (2013a). *Arqueozoología y Romanización. Producción, distribución y consumo de animales en el noreste de la península ibérica entre los siglos V a. n. e. - V d. n. e.* Oxford: Hadrian Books. (BAR International Series; 2480).
COLOMINAS, L. (2013b). “Gestió i explotació ramadera a l'Olivet d'en Pujol (Viladamat, Alt Empordà) durant la segona meitat del segle I aC: l'exemple d'un model ramader totalment romanitzat”. *Annals.* Girona: Institut d'Estudis Gironins, núm. 54, p. 185-198.

COLOMINAS, L. (2017). “Roman conquest and changes in animal husbandry in the North-East of the Iberian Peninsula: Searching for patterns, rates and singularities”. In VALENZUELA LAMAS, S.; COLOMINAS, L.; FERNÁNDEZ RODRÍGUEZ, C. (ed.). *La Romanización de la Península Ibérica, una visión desde la Arqueozoología. Archaeofauna: International Journal of archaeozoology.* Madrid: Universidad Autónoma de Madrid, núm. 26, p. 9-22.

COLOMINAS, L.; SAÑA, M. (2009a). “Dinàmica de formació i variabilitat dels conjunts de restes de fauna recuperats al jaciment del Bosc del Congost: gestió animal entre el 325 aC i el 100aC”. In BURCH, J.; SAGRERA, J. (ed.). *Els sitjars*. Girona i Sant Julià de Ramis: Universitat de Girona i Ajuntament de Sant Julià de Ramis, p. 155–78. (Excavacions arqueològiques a la muntanya de Sant Julià de Ramis; 3).

COLOMINAS, L.; SAÑA, M. (2009b). “Animal husbandry in the North-East of Catalonia from the 1st to the 5th Century AD: improvement and importation”. *The territory and its resources*. Girona: Laboratori d'Arqueologia i Prehistòria de la Universitat de Girona, p. 9-26 (Studies on the Rural World in the Roman Period; 4).

COLOMINAS, L.; ANTOLÍN, F.; FERRER, M.; CASTANYER, P.; TREMOLEDA, J. (2017a). “From Vilauba to Vila Alba: Changes and continuities in animal and crop husbandry practices from the Early Roman to the beginning of the Middle Ages in the north-east of the Iberian Peninsula”. *Quaternary International*. Vol. 499. Amsterdam: Elsevier, p. 67-79.

COLOMINAS, L.; RODRÍGUEZ, C. F.; ERES, M. P. I. (2017b). Animal husbandry and hunting practices in Hispania Tarraconensis: an overview. *European Journal of Archaeology*, *20*(3), 510.

ESTÉVEZ, J. (1977). “Estudio de la fauna”. In PONS, E. (dir.). *La Fonollera (Torroella de Montgrí, Girona). Un poblado al aire libre del Bronce Final.* Girona: Centre d'Investigacions Arqueològiques. (Sèrie Monogràfica; 1).

ESTRADA, A.; NADAL, J. (2007). “La Solana. Estudi zooarqueològic del jaciment”. In BARRASETAS, E. (coord.). *La Solana. Memòria de l'excavació arqueològica al jaciment (Cubelles, el Garraf)*. Barcelona: Departament de Cultura i Mitjans de Comunicació de la Generalitat de Catalunya, Barcelona. (Memòries d'Intervenció Arqueològica).

FOLCH, C., GIBERT, J. (2014). “L'Aubert (la vall d'en Bas, la Garrotxa) (segles VI-X dC): un assentament rural en els orígens del Comitatus Bisuldunense”. *Quaderns de les*

Assemblees d'Estudis. Centre d'Estudis Amics de Besalú i el seu comtat, núm. 1, p. 143-156.

FONT, L. (2016). *La gestió dels recursos animals a la Catalunya meridional i de ponent durant la protohistòria (segles VII-I a. n. e.). Avaluació econòmica, política i social a partir de les restes de fauna*. PhD. Barcelona: Departament d'Història i Arqueologia, Secció de Prehistòria i Arqueologia, Universitat de Barcelona.

GÓMEZ, X. (2000). *Noves dades sobre l'explotació de base animal durant la protohistòria a la plana occidental catalana*. Lleida: Departament d'Història, Universitat de Lleida. Licence These unpublished.

LIGNEREUX, Y.; PÉRIN, N. (2006). "Les restes fauniques de Torre Cremada". In MORET, P. M.; BENAVENTE, J. A.; GORGUES, A. (ed.). *Iberos del Matarraña: investigaciones arqueológicas en Valdeltormo, Calaceite, Cretas y La Fresneda (Teruel)*. Alcañiz: Taller de Arqueología de Alcañiz i Casa de Velázquez, p. 293-298. (Al-Quannis, Boletín del Taller de Arqueología de Alcañiz; 11).

MAÑOSA, M. (1990). *Informe de l'anàlisis de la fauna del jaciment de Can Feu (St. Quirze del Vallès, Vallès Occidental.* Barcelona: Departament de Cultura, Servei d'Arqueologia de Barcelona, Generalitat de Catalunya. Faunal report.

MIRÓ, J. M. (1989a). "Estudi de la fauna". In GALLART, J.; JUNYENT, E. (ed.). *Un nou tall estratigràfic a la Pedrera, Vallfogona de Balaguer (Termens, La Noguera, Lleida)*. Lleida: Quaderns del Departament de Geografia i Història de l'Estudi General de Lleida, p. 80-101. (Col·lecció Espai/Temps; 3).

MIRÓ, J. M. (1989b). "La fauna". In DUPRÉ, X. (ed.). *Un abocador del segle V d. C. en el fòrum provincial de Tarraco.* Tarragona: Taller Escola d'Arqueologia, p. 403-414. (Memòries d'Excavacio; 2).

MIRÓ, J. M. (1992). "La Macrofauna". In GARCÉS, I.; JUNYENT, E.; LAFUENTE, Lafuente; LÓPEZ MELCIÓN, Joan B. (dir.). *Dinàmica Paleoceològica i Paleoeconòmica en l'assentament de "Els Vilars" (Arbeca, les Garrigues)*. Lleida: Secció d'Arqueologia Prehistòria i Història Antiga, Departament d'Història, Universitat de Lleida. Faunal report.

MIRÓ, C.; MOLIST, N. (1982). "La fauna". In BARBERÀ, J.; SANMARTÍ GREGO, E. (ed.). *Excavacions al poblat ibèric de la Penya del Moro de Sant Just Desvern (Barcelonès): campanyes 1974-1975 i 1977-1981*. Barcelona: Diputació de Barcelona, Institut de Prehistòria i Arqueologia, p. 123-132. (Monografies Arqueològiques; 1).

MOLINA VALLMITJANA, J. A.. (2006). "Annex 26: Estudi de les restes arqueozoològiques dels nivells tardoantics (s. VI-VII) de Can Gambús-1 (Sabadell, Vallès Occidental)". In ROIG, J.; COLL RIERA, J. M. (2006). *Memòria d'intervenció arqueològica a Can Gambus 1 (Sabadell, Vallès Occidental). Abril 2003-Desembre 2004 i agost 2006*. Arrago SL. Barcelona: Departament de Cultura, Servei d'Arqueologia: Generalitat de Catalunya. (Memòries d'Intervenció Arqueològica; 7416, vol. 22).

MOLINA VALLMITJANA, J. A. (2008-2009). "Estudi de les restes arqueozoològiques del jaciment de Can Sant Joan". In: Gea Bullich, M. (2008-2009). *Memòria de la intervenció arqueològica a Can Sant Joan, parcel·la 15A, Rubí. (novembre 2008*). Arxiu Servei d'Arqueologia i Paleontologia. Mem.Núm. 7323.

MOLINAS AMORÓS, R. (2006). *Informe arqueològic sobre la gestió dels recursos animals al jaciment de Plaça Constitució (Sant Boi)*. ArqueoCat SL: Arqueologia i Patrimoni. Faunal report.

MONTERO, M. (2000). *Les restes de macrofauna del jaciment de Ciutadella de Roses (Girona)*. Barcelona: Laboratori d'Arqueozoologia, Departament de Prehistòria, Universitat Autònoma de Barcelona. Citat per Colominas i Saña, 2011. License These unpublished.

NADAL, J.; ESTRADA, A. (2003). "Estudi de les restes faunístiques de Puig Castellar (Annex 3)". In FERRER, C.; RIGO, A. (ed.). *Els ibers a Santa Coloma de Gramanet.* 5 anys d'intervenció arqueològica (1998-2002). Santa Coloma de Gramanet: Museu Torre Balldovina, Ajuntament de Santa Coloma de Gramanet, p. 174-182. (Monografies locals; 2).

NADAL, J.; ESTRADA, A. (2007b). "Estudi de fauna". In FRANCÈS, J. (coord.). *Els Mallols. Un jaciment de la plana del Vallès entre el neolític i l'antiguitat tardana.* Barcelona: Departament de Cultura i Mitjans de Comunicació, Generalitat de Catalunya, p. 265-301. (Excavacions arqueològiques a Catalunya; 17).

NIETO ESPINET, A. (2002). *Anàlisi arqueofaunístic del jaciment protohistòric de Vincamet (Fraga, Huesca)*. TAD (Treball Acadèmicament Dirigit). Lleida: Departament d'Història, Universitat de Lleida.

NIETO ESPINET, A. (2012). *Entre el consum i l'afecte. La interacció entre els animals i les comunitats protohistòriques de la plana occidental catalana (segles VII-IV aC)*. Lleida: Departament d'Història, Universitat de Lleida. Accés lliure al TDX (https://www.tdx.cat/handle/10803/96488).

NIETO ESPINET, A. (2013). *Informe de les restes faunístiques recuperades al jaciment de la Rosella (Tàrrega, Lleida)*. Lleida: Departament d'Història, Universitat de Lleida. Faunal report.

NIETO ESPINET, A. (2014). *Informe de les restes faunístiques recuperades Sikarra (Prats del Rei, Barcelona)*. Lleida: Departament d'Història, Universitat de Lleida. Faunal report.

NIETO ESPINET, A.; ESCALA, Ò. (2004). "Vilans de Reig (els Torms, les Garrigues): un assentament rural del s. VII dC". *Revista d'arqueologia de Ponent*, (14), 275-286.

NIETO ESPINET, A.; VALENZUELA LAMAS, S.; TRENTACOSTE, A. (2019). *Informe de la fauna recuperada al jaciment de Mas d'en Boixos (Vilafranca del Penèdes, Barcelona)*. Faunal report, Vinseum, Museu de Vilafranca.

NIETO ESPINET, A.; VALENZUELA LAMAS, S. (2019) *Informe de la fauna recuperada al jaciments de Can Mateu, Can Rodon i Can Benet (Cabrera de Mar, Barcelona)*, Faunal report, Museu de Mataró.

NIETO ESPINET, A.; VALENZUELA LAMAS, S. (2020a) *Informe de la fauna recuperada al jaciment del Molí Espígol (Tornabous, Lleida)*, Faunal report, MAC-Barcelona.

NIETO ESPINET, A.; VALENZUELA LAMAS, S. (2020b) *Primers resultats de l'estudi de la fauna recuperada en els nivells romans de l'antiga Ilerda (Lleida)*. Faunal report, Servei d'Arqueologia, Ajuntament de Lleida.

OLIVA, M. A. (1998). *Anàlisi arqueozoològica de l'assentament ibèric del Turó del Vent (Llinars del Vallès).*

Treball de recerca. Barcelona: Departament d'Antropologia Social i Prehistòria, Universitat Autònoma de Barcelona.
OLIVA, M. A. (2000). "La ramaderia del poblat ibèric del Turó del Vent". *SAGVNTVM Extra*. Vol. 3. València: Departament de Prehistòria i Arqueologia de la Universitat de València, p. 165-166.

ORRI, E. (2005-2006). "Informe de l'estudi de la fauna recuperada al jaciment de Can Gambus-3 (Sabadell, Vallès Occidental)". In CODINA, D.; ARTIGUES, P. L. *Memòria de la intervenció arqueològica a Can Gambús 3*. Agost del 2005-2006. Sabadell (Vallès Oriental). Janus SL. Barcelona: Departament de Cultura, Servei d'Arqueologia: Generalitat de Catalunya. (Memòries d'Intervenció Arqueològica; 7017). Faunal report.

ORRI, E.; ESTRADA, A. (2004). "Annex III. Estudi de les restes faunístiques i malacològiques". In REVILLA, V.; CELA, X. *La transició del municipium d'Iluro a Alarona (Mataró): Cultura material i transformacions d'un espai urbà entre els segles V i VII dC. Laietània: estudis d'arqueologia del Maresme*. Mataró: Museu de Mataró, núm. 15, p. 543-555.

PADRÓS, N.; VALENZUELA LAMAS, S. (2010). "La Llosa i els Antigons, una aproximació a la producció ramadera de les *villae* de l'*ager Tarraconensis*. Segles III-VI dC". In PREVOSTI, M.; GUITART, J. (ed.). *Ager Tarraconensis 1. Aspectes històrics i marc natural*. Tarragona: Institut Català d'Arqueologia Clàssica, p. 200-207. (Documenta; 16).

PIÑA, A.; SAÑA, M. (2004). "Informe de l'anàlisi arqueozoològica dels conjunts de restes de fauna recuperats al jaciment de Can Roqueta / Torre-romeu (Sabadell, Vallès Occidental)". In OLIVA, M., TERRATS, N. (2004). *El jaciment arqueològic de Can Roqueta / Torre Romeu (Sabadell, Vallès Occidental). Campanya 2002-2003*. Barcelona: Departament de Cultura, Servei d'Arqueologia: Generalitat de Catalunya. Faunal report.

VALENZUELA LAMAS, S. (2008). *Alimentació i ramaderia al Penedès durant la protohistòria: segles VII-III aC*. Barcelona: Societat Catalana d'Arqueologia.

VALENZUELA LAMAS, S. (2010a). "Paisatge, alimentació i gestió de ramats als Antigons a partir de les restes de fauna (vertebrats i mol·luscos)". In PREVOSTI, M.; GUITART, J. (ed.). *Ager Tarraconensis 1. Aspectes històrics i marc natural*. Tarragona: Institut Català d'Arqueologia Clàssica, p. 181-192. (Documenta; 16).

VALENZUELA LAMAS, S. (2012). *Informe de les restes faunístiques de la vil·la romana del Vilarenc*. Barcelona: Departament d'Història i Arqueologia, Secció de Prehistòria i Arqueologia, Universitat de Barcelona. Faunal report.

VALENZUELA LAMAS (2017). *Informe de la fauna recuperada a la zona 14 del Puig de Sant Andreu (Ullastret, Girona)*. Faunal report, MAC-Ullastret.

VALENZUELA LAMAS, S.; NAVARRO, R. (2007). "Un conjunt faunístic de finals de l'Antiguitat tardana al jaciment de Santa Margarida (Martorell, Baix Llobregat)". *Pyrenae*. Barcelona: Departament d'Història i Arqueologia, Secció de Prehistòria i Arqueologia, Universitat de Barcelona, núm. 38, p. 105-127.

VALENZUELA LAMAS, S.; NADAL, J.; LÓPEZ, D. (2010). "La fauna del jaciment de Mas d'en Gual (el Vendrell, Baix Penedès) en el context dels assentaments especialitzats d'època ibèrica". *Revista d'Arqueologia de Ponent*. Lleida: Departament d'Història, Universitat de Lleida, núm. 20, p. 215-224.

VALENZUELA LAMAS, S.; NIETO ESPINET, A. 2020. "Noves evidències de la pràctica d'un banquet en el món ibèric? Els conjunts faunístics de tres sitges del jaciment de Can Rodon de l'Hort (Cabrera de Mar, El Maresme)". *Laietania,* núm. 21, p. 57-75.

VALENZUELA LAMAS, S., PADRÓS, N. (2010). Què diu la mida dels animals? Osteometria dels macromamífers de la *Tarraconensis* entre l'edat del ferro i l'època imperial, *Simposi Internacional Ager Tarraconensis*, Tarragona, octubre 2010.

VALENZUELA LAMAS, S.; NIETO ESPINET, A. (2018) *Informe de la fauna recuperada al jaciment de Sant Martí d'Empúries (Empúries, Girona)*. Faunal report, MAC-Empúries.

VALENZUELA LAMAS, S.; NIETO ESPINET, A. (2019) *Informe de la fauna recuperada al jaciment de Can Bartomeu (Cabrera de Mar, Barcelona)*. Faunal report, Museu de Mataró.

VALENZUELA LAMAS, S.; NIETO ESPINET, A. (2020a) *Informe de la fauna recuperada al jaciment de Can Cruzate (Mataró, Barcelona)*. Faunal report, Museu de Mataró.

VALENZUELA LAMAS, S.; NIETO ESPINET, A. (2020b) *Informe de la fauna recuperada a la domus romana de Torre Llauder (Mataró, Barcelona)*, Faunal report, Museu de Mataró.

VALENZUELA LAMAS, S.; VALENZUELA SUAU, L.; JIMÉNEZ MANCHÓN, S.; CODINA, F.; PLANA, R.; MARTÍN, A. (2017). "Consum carni i artesanat en os als afores del Puig de Sant Andreu: el jaciment ibèric del Camp d'en Gou, Gorg d'en Batlle (Ullastret, Baix Empordà)". *Empúries*. Girona: Museu d'Arqueologia de Catalunya, núm. 57, p. 7-26.
