# Supplementary material for: Resilience and livestock adaptations to demographic growth and technological change: A diachronic perspective from the Late Bronze Age to Late Antiquity in NE Iberia
Source: PLoS One. 2021 Feb 17;16(2):e0246201. doi: 10.1371/journal.pone.0246201 (PMC7888671; doi:10.1371/journal.pone.0246201)

types and geographical areas of sites (n=10) during the 'LBA' period

num	Site
14a	Can Gambus 3
15a	Can Roqueta CRV
15b	Can Roqueta TR
16	Carretela
18	Cova Punta Farisa
21	Fonollera
43a	Sant Marti Empuries
63	Vilot de Montagut 0_I_II_III
64	Vincament
65	Zafranales

type.of.site

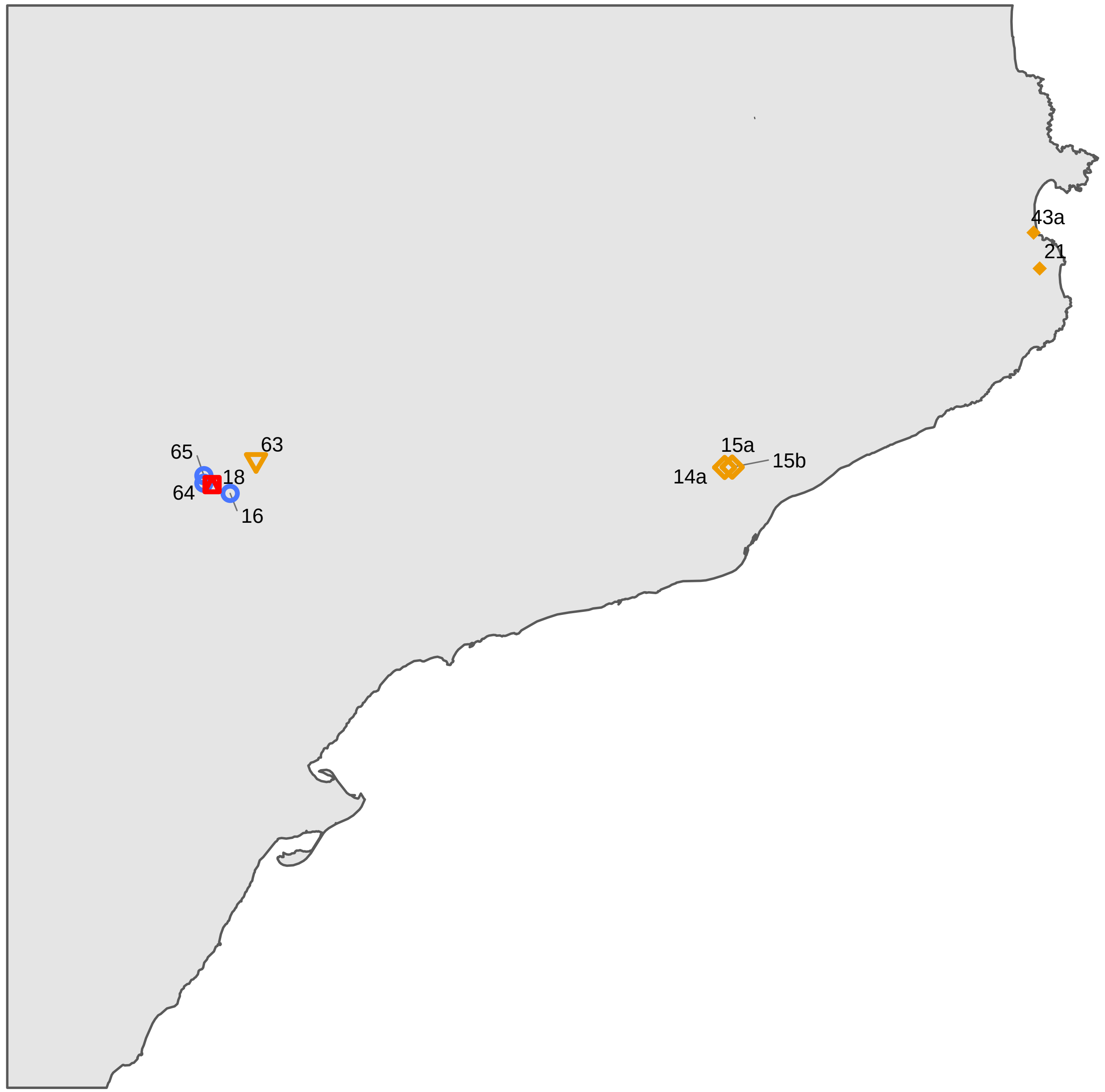

geographical.area

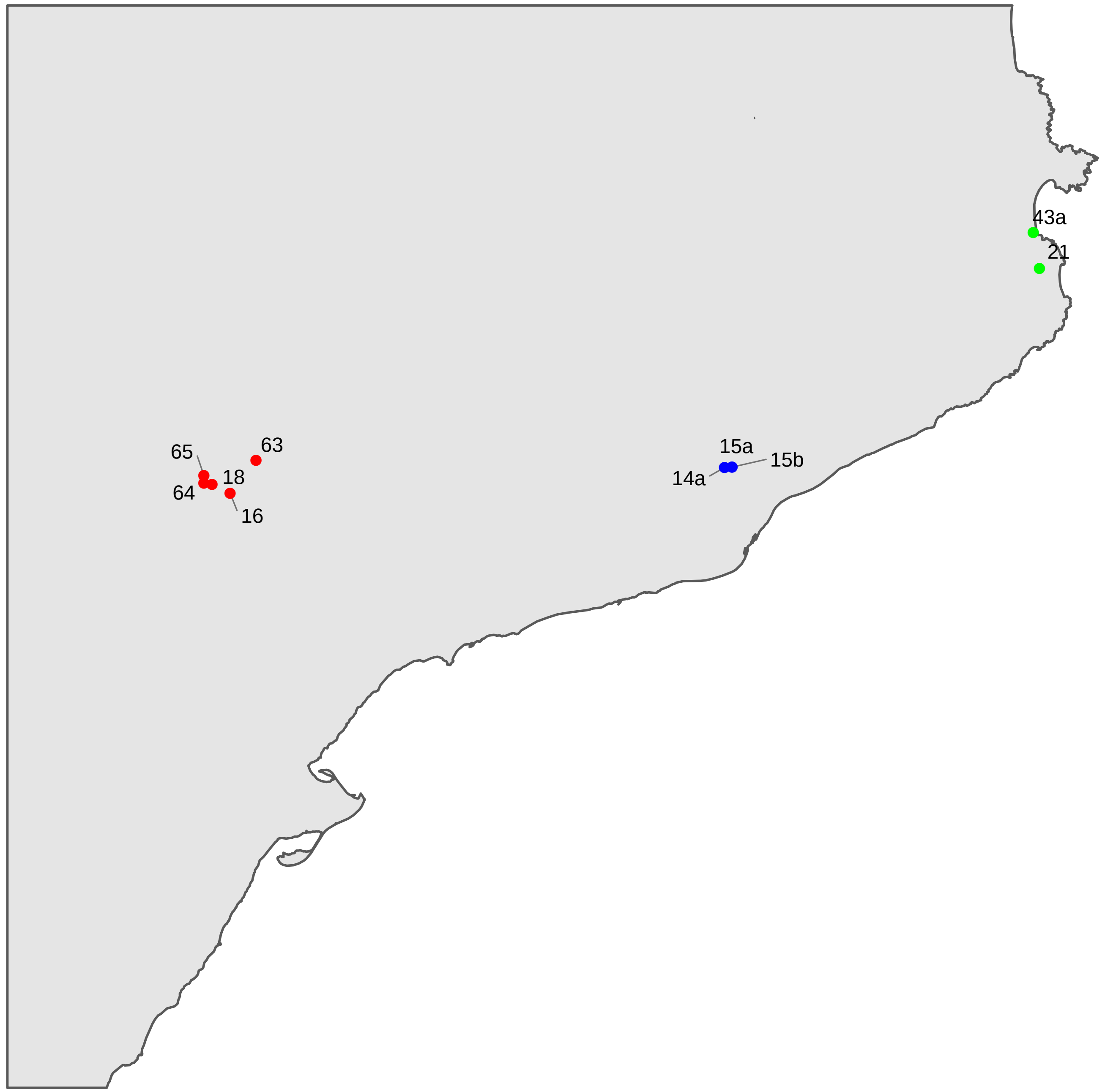

types and geographical areas of sites (n=14) during the 'EIA' period

num	Site
107	Mas Duran
15c	Can Roqueta_DIASA
37	La Pedrera IV_V_VI_VII
39	Puig de la Misericordia
40a	Puig de la Nau
42	Sant Jaume Mas d en Serra_sect 1
43b	Sant Marti Empuries
48	Sitges UAB
55a	Tossal Molinet I_II
57a	Turo Font de la Canya 0
59a	Vilars 0_I
6a	Barranc de Gafols_1
6b	Barranc de Gafols_2
90a	Mas d en Boixos

type.of.site

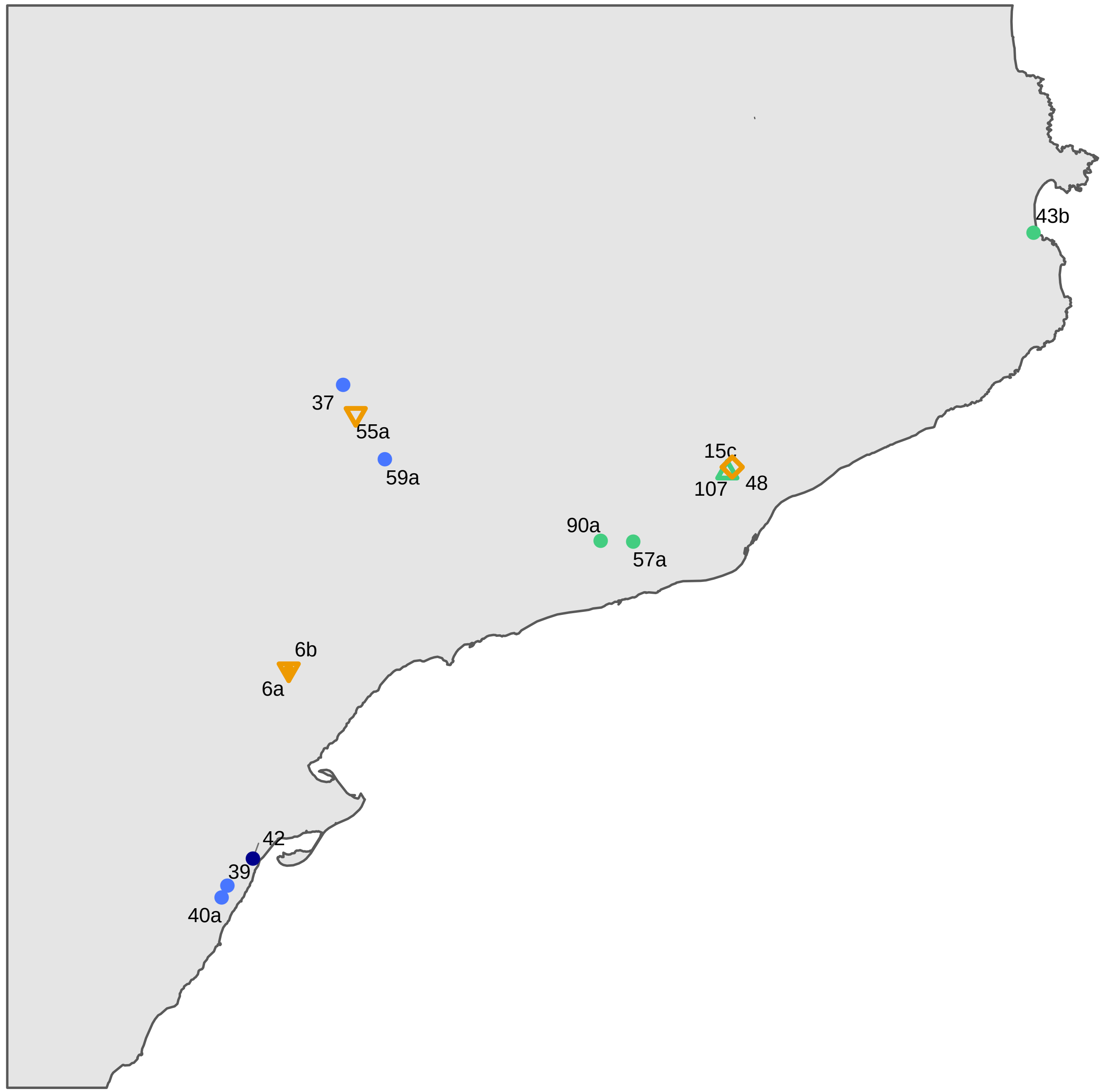

geographical.area

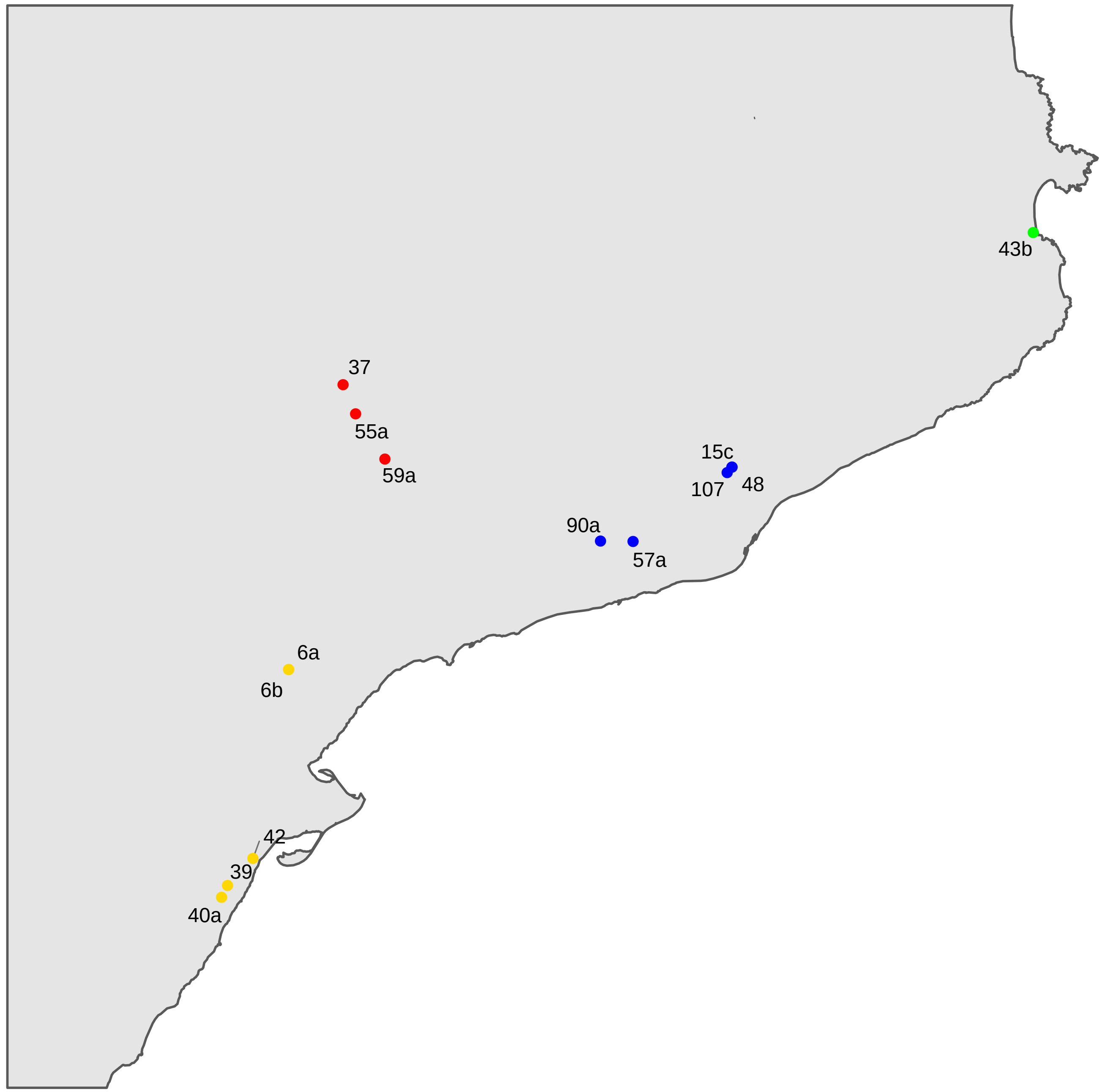

types and geographical areas of sites (n=14) during the 'MIA1' period

num	Site
14b	Can Gambus 3
19a	Empuries
24a	Illa d en Reixach 2_3
2a	Alorda Park 2a
33a	Moli Espigol
40b	Puig de la Nau
43c	Sant Marti Empuries
44a	Sant Sebastia de la Guarda
55b	Tossal Molinet III
57b	Turo Font de la Canya 1
59b	Vilars II
89a	Torre Roja
90b	Mas d en Boixos
9a	Ca n Oliver 1

type.of.site

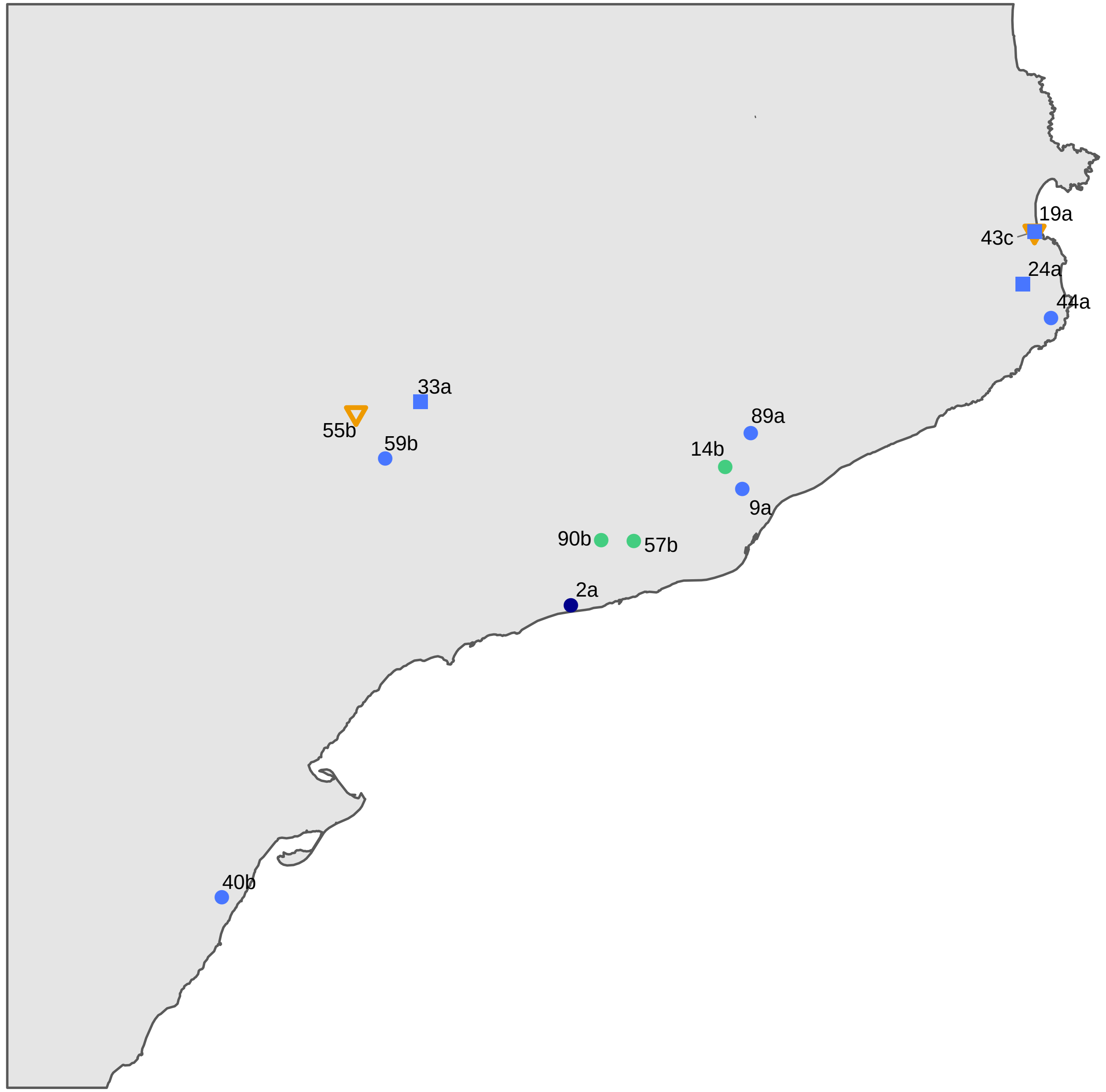

geographical.area

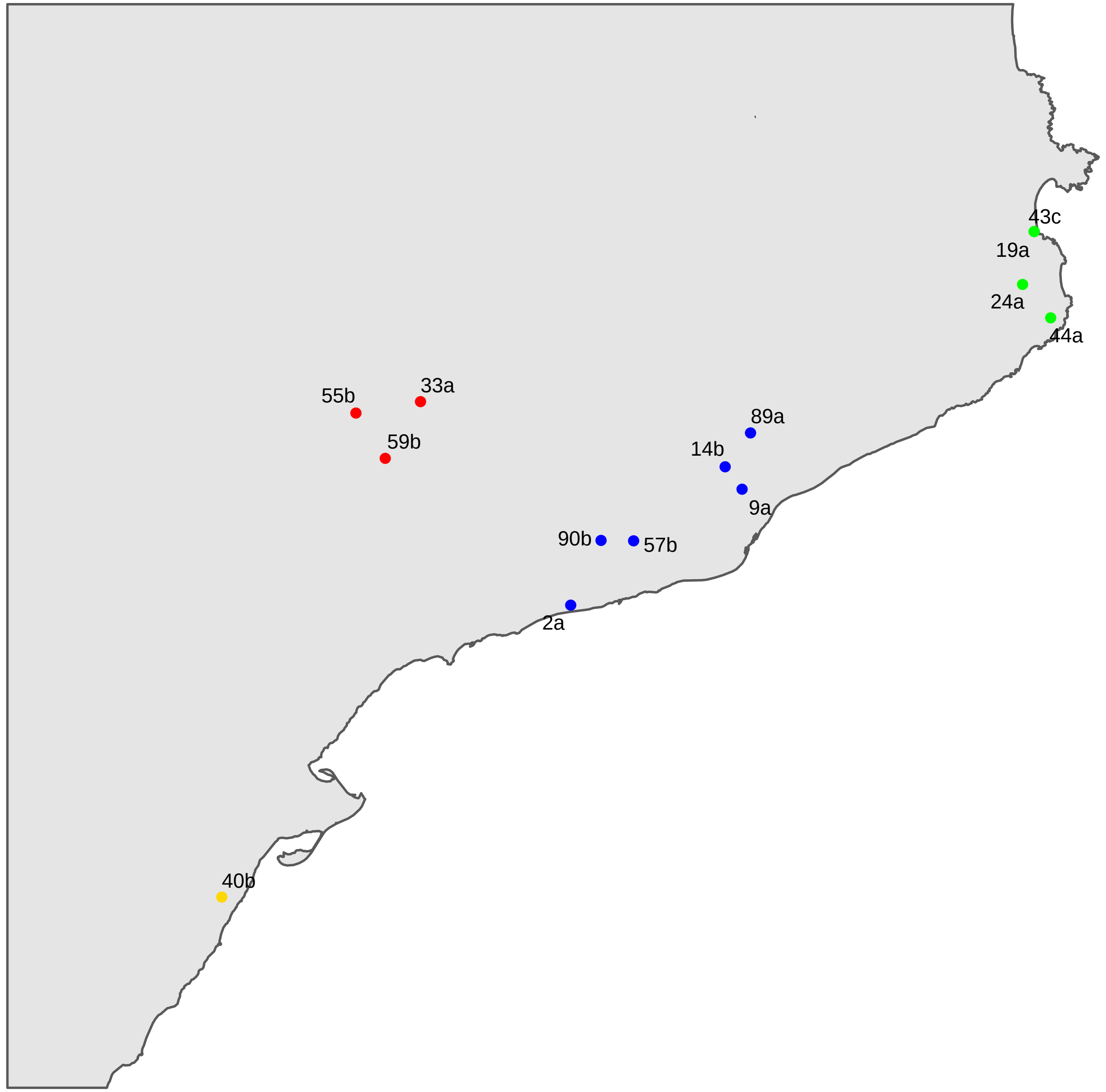

types and geographical areas of sites (n=28) during the 'MIA2' period

num	Site
1	Esquerda
103a	Can Rodon
10a	Can Bartomeu
14c	Can Gambus 1
17	Ciutadella de Roses
22	Gou Batlle
24b	Illa d en Reixach 4_5_6
25	Puig Castellar
29a	Mas Castellar fortificat
29b	Mas Castellar rural
2b	Alorda Park 2b
32	Moleta del Remei 1_2
33b	Moli Espigol
34a	Olerdola 1
35	Olius
38	Penya del Moro
46	Saus
47a	Sigarra
50a	St. Julia de Ramis
56	Turo del Vent 1_2
57c	Turo Font de la Canya 2_3
59c	Vilars III_IV
71	Coll del Moro
78	Mas d en Gual
7a	Bosc del Congost
88	Puig de Sant Andreu
89b	Torre Roja
9b	Ca n Oliver 2_3

type.of.site

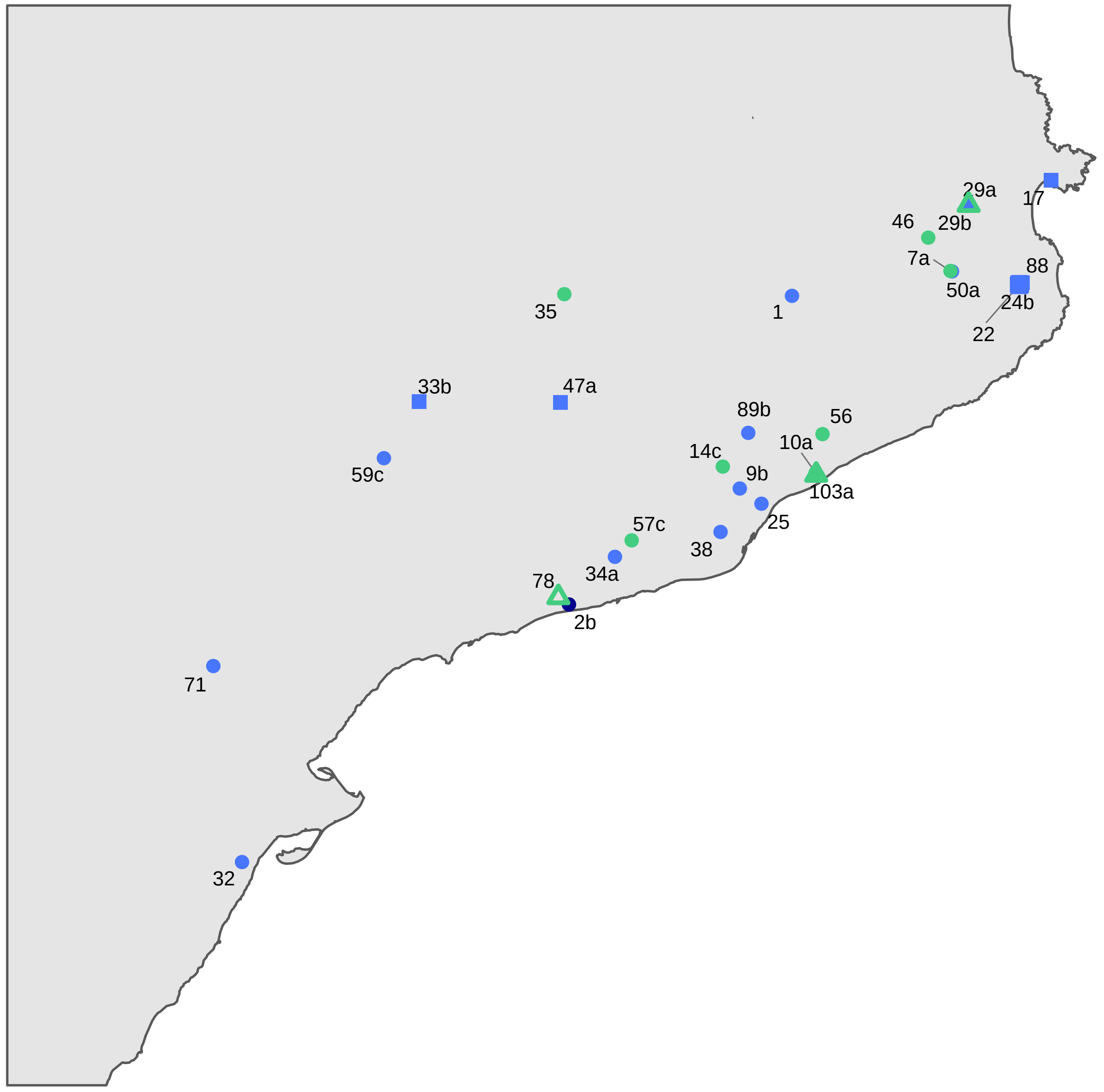

geographical.area

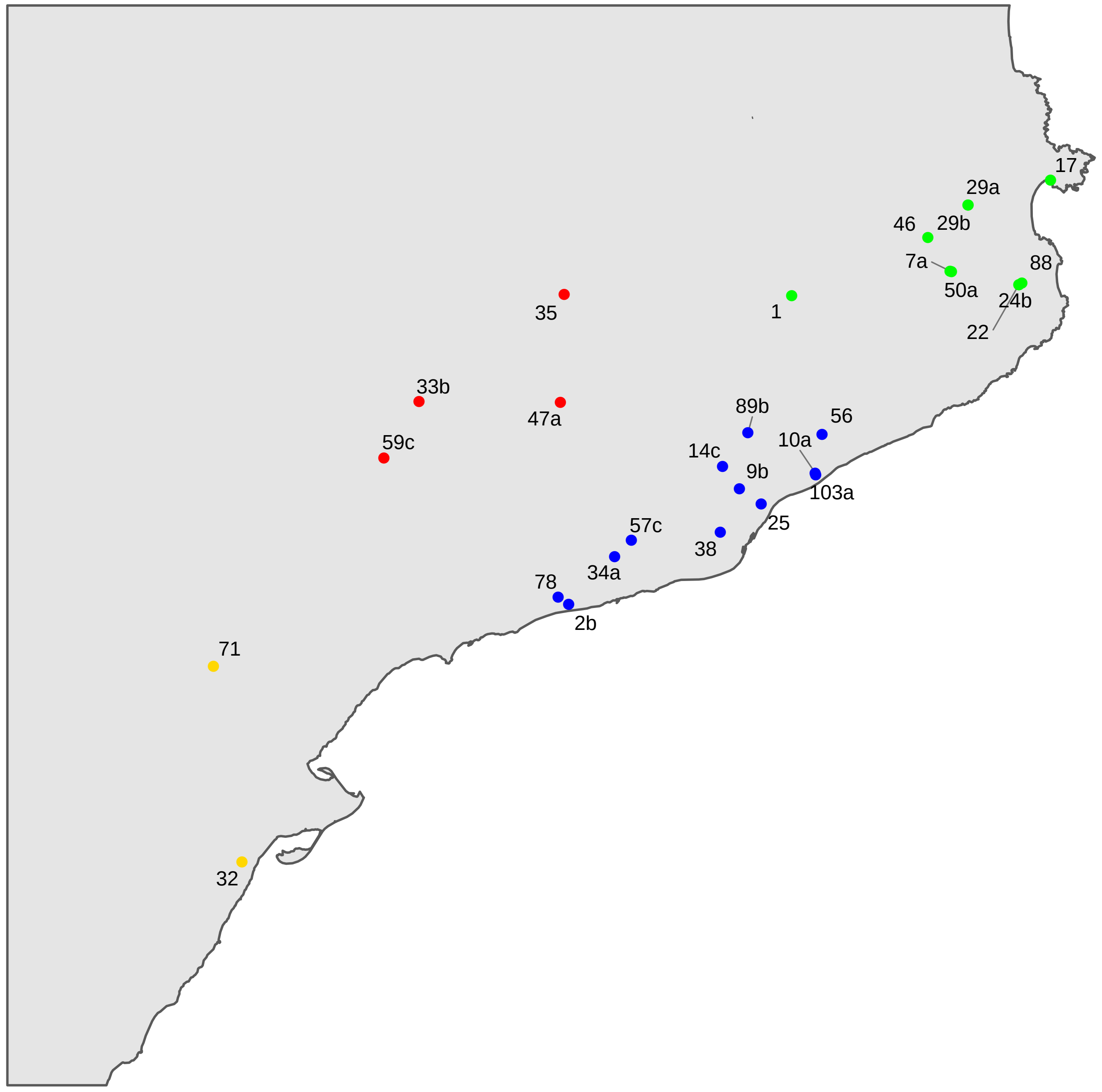

types and geographical areas of sites (n=23) during the 'RR' period

num	Site
101	Can Mateu
102	Can Bernat
10b	Can Bartomeu
12b	Can Feu
15d	Can Roqueta TR
23a	Ilerda
29c	Mas Castellar
30	Mas Guso
34b	Olerdola 2
36	Olivet d en Pujol
41b	Sant Boi_Pl Constitucio
44b	Sant Sebastia de la Guarda
47b	Sigarra
50b	St. Julia de Ramis
53	Torre Cremada
58a	Vilarenc
7b	Bosc del Congost
8	Burriac
84	Rosella
87	Puig Castellar Biosca
89c	Torre Roja
91	Hereuet
92	Missatges

type.of.site

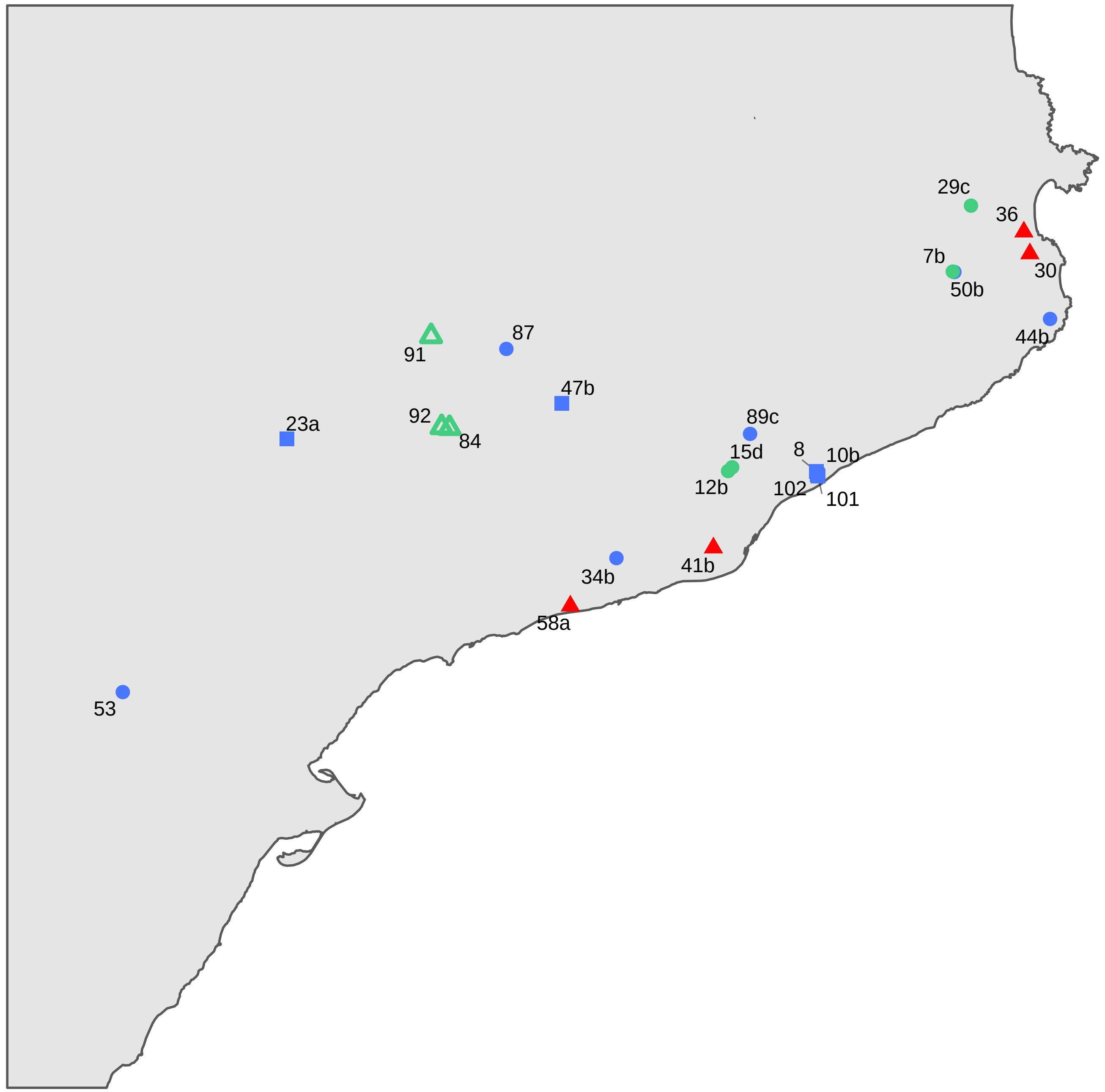

geographical.area

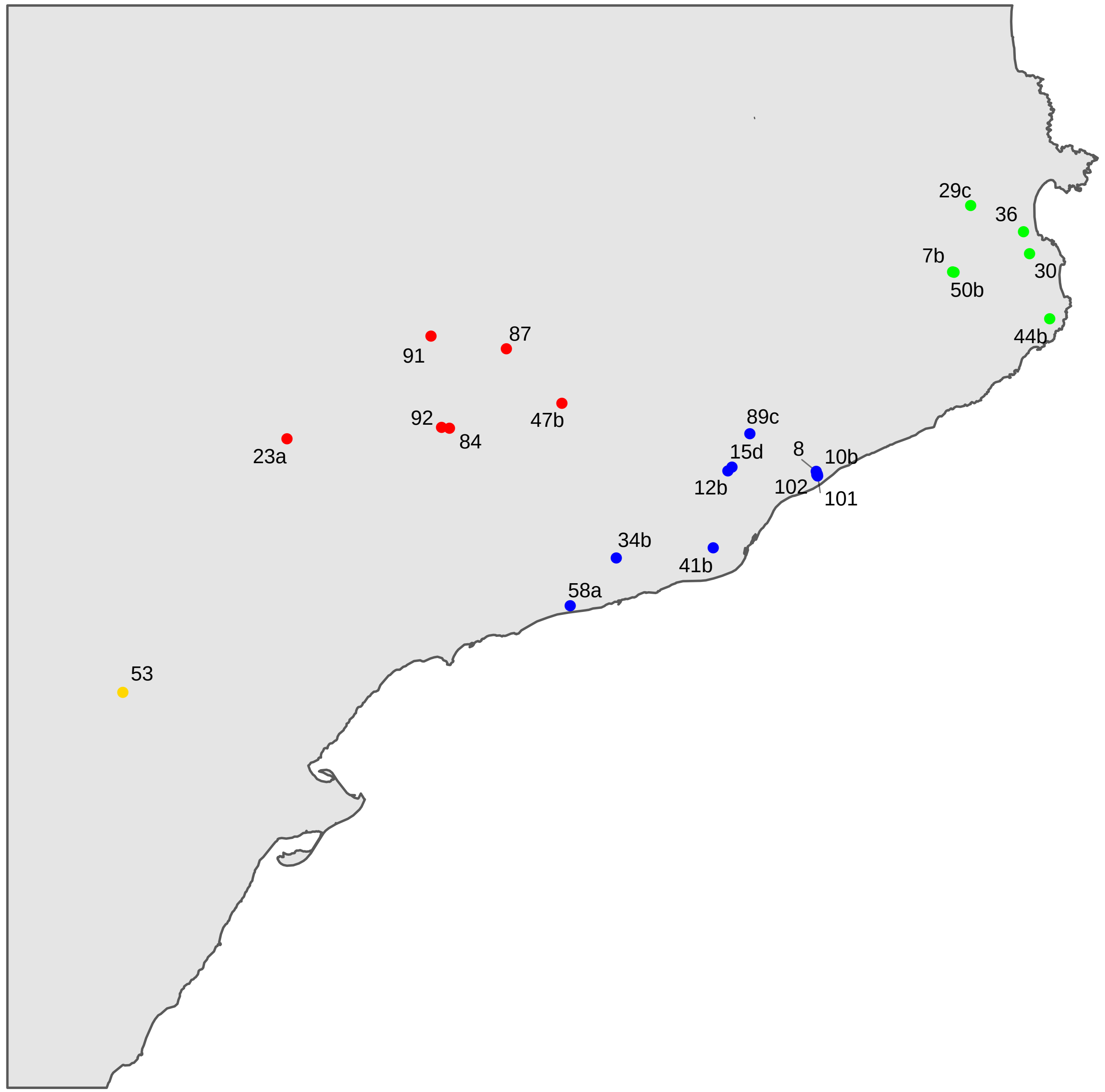

types and geographical areas of sites (n=18) during the 'ERE' period

num	Site
103b	Can Rodon
106	Can Sant Joan
11b	Can Cruzate
12a	Can Feu
19b	Empuries
23c	Ilerda _Carrer Magdalena 47
23d	Ilerda_Carrer Bafart 46
27a	La Llosa
3a	Antigons_abocador
41c	Sant Boi_Pl Constitucio
5	Baetulo
52	Tolegassos
54a	Torre Llauder
58b	Vilarenc
60a	Vilauba
61a	Vil la Vinyet
94	Iesso
95	Vil la Torre Andreu

type.of.site

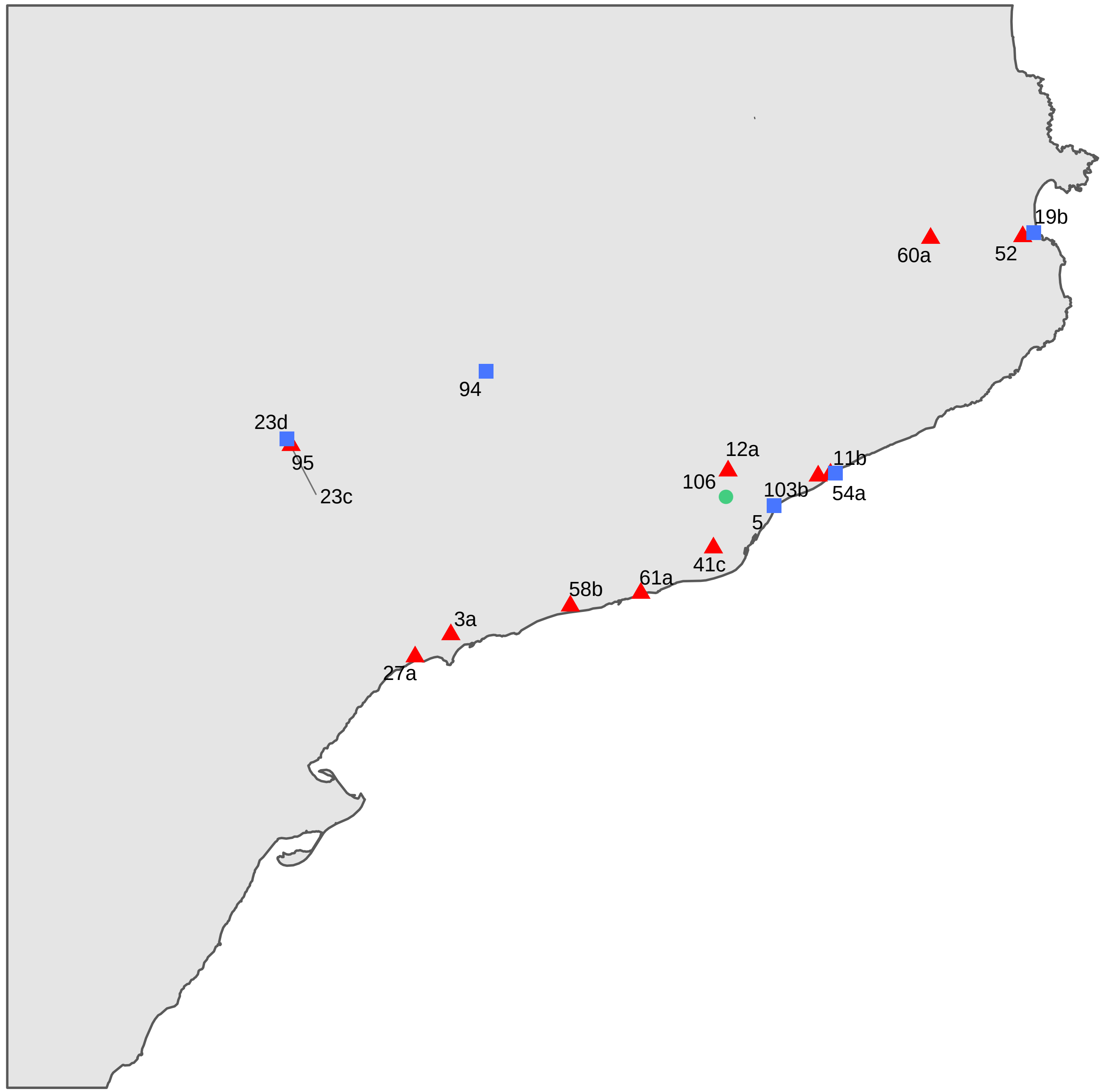

geographical.area

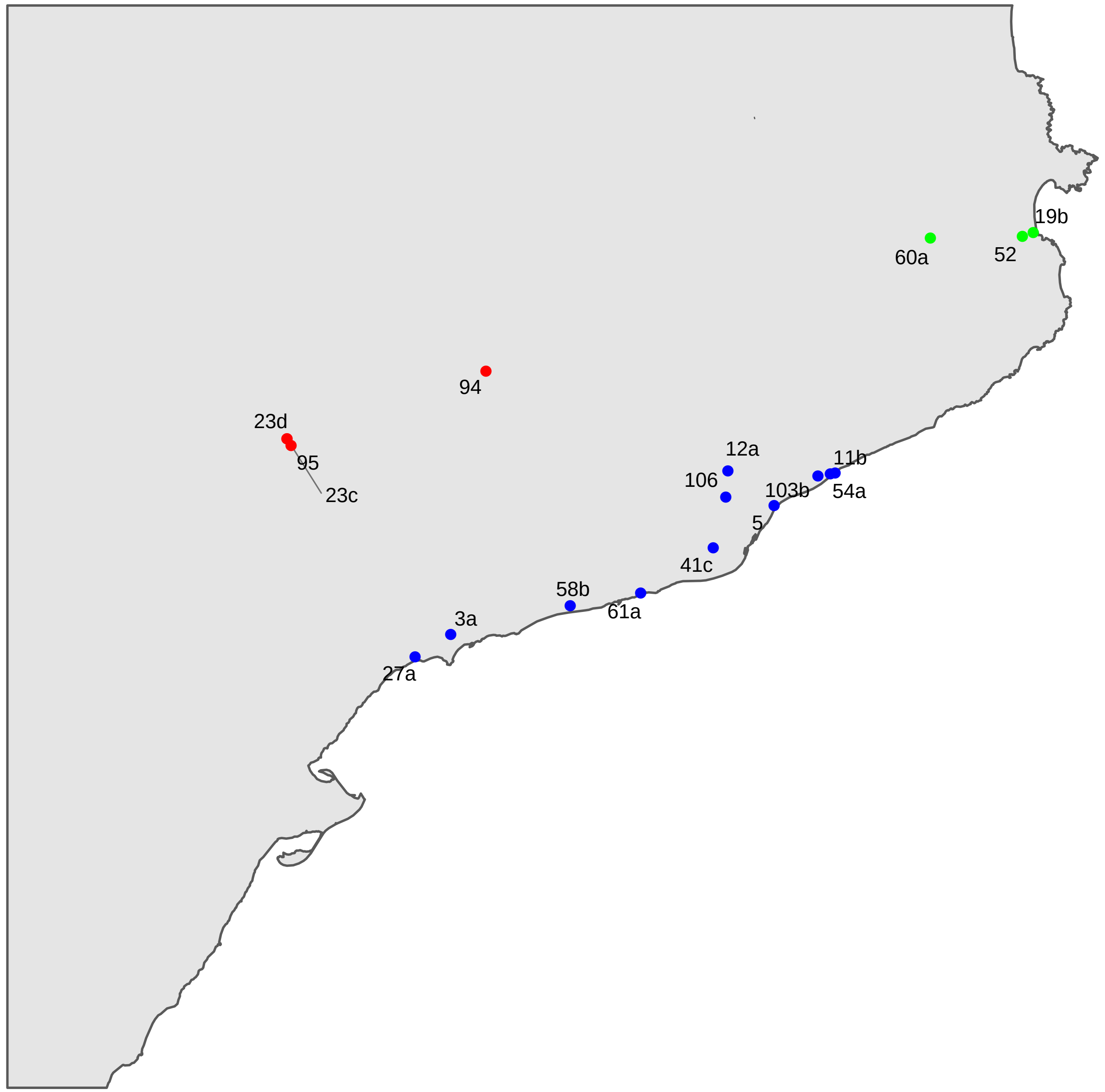

types and geographical areas of sites (n=10) during the 'LRE' period

num	Site
11a	Can Cruzate
23b	Ilerda
27b	La Llosa
3b	Antigons_Nymphaeum
41d	Sant Boi_Pl Constitucio
51	Tarraco
54b	Torre Llauder
60b	Vilauba
61b	Vil la del Vinyet
62	Vil la dels Ametllers

type.of.site

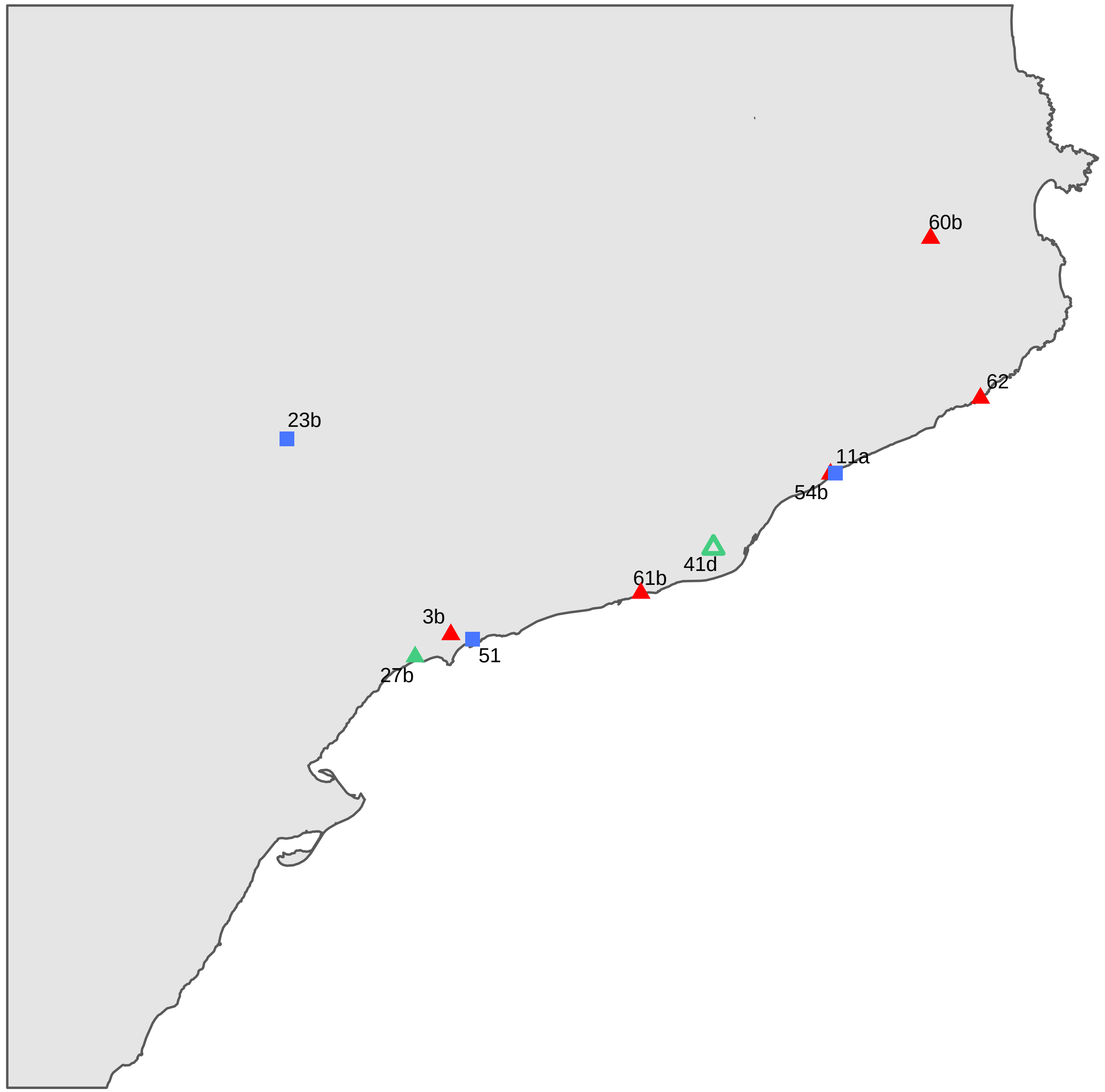

geographical.area

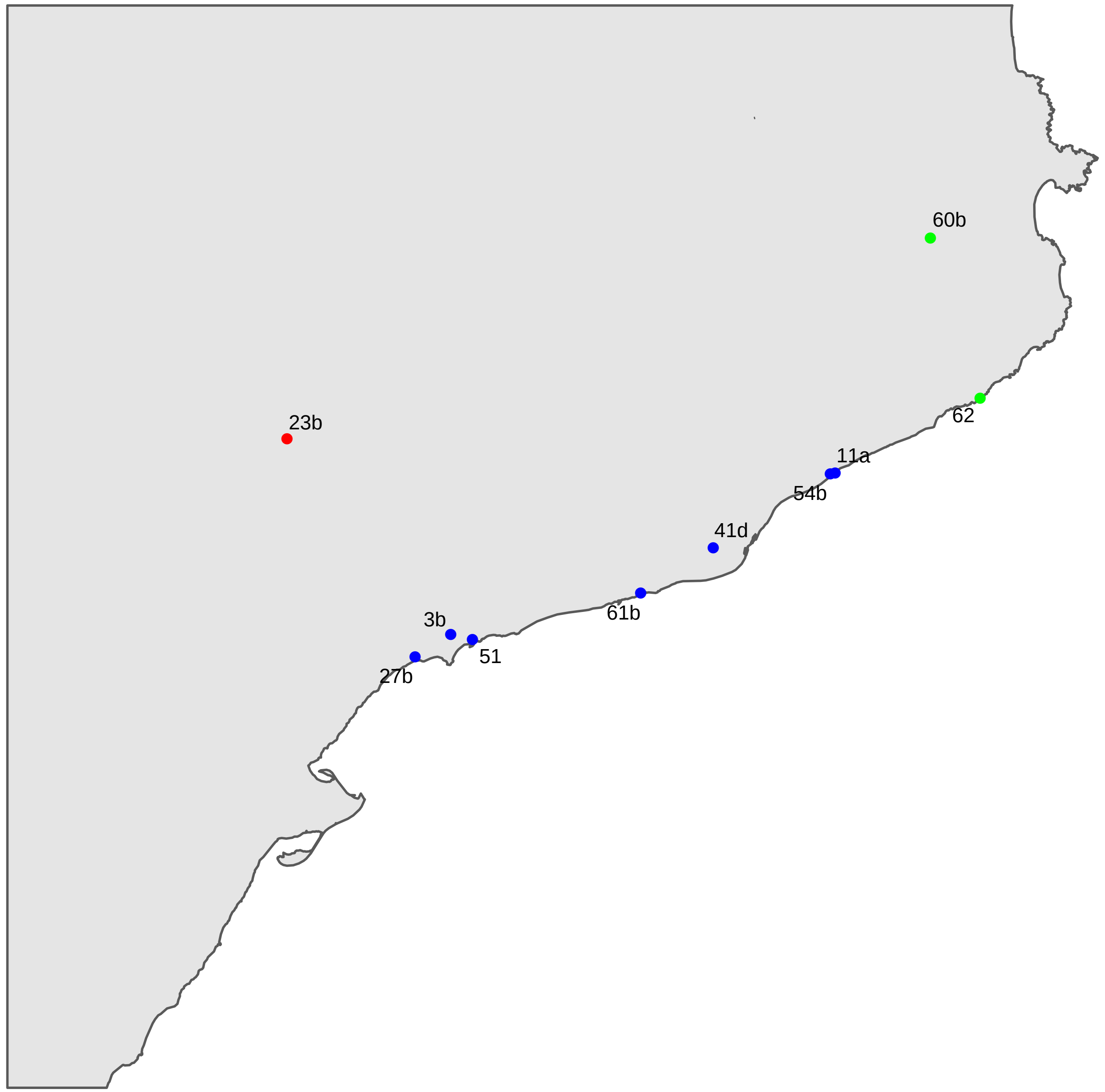

types and geographical areas of sites (n=11) during the 'LA' period

num	Site
14d	Can Gambus 1
19c	Empuries
26	Iluro_VI_VII
28	Mallols
4	Aubert
43d	Sant Marti Empuries
45	Santa Margarida
49	Solana
54c	Torre Llauder
60c	Vilauba
97	Vilans de Reig

type.of.site

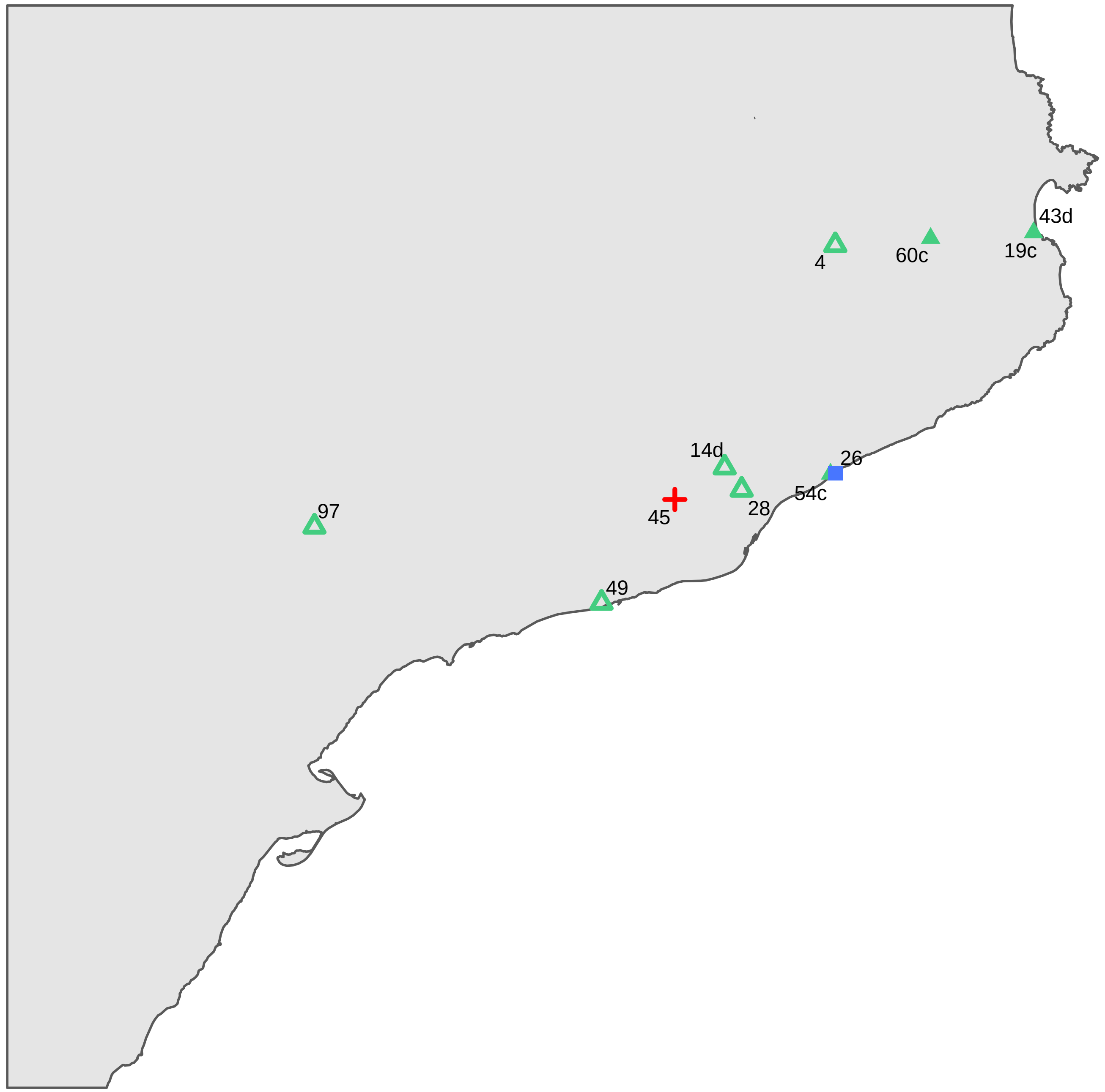

geographical.area

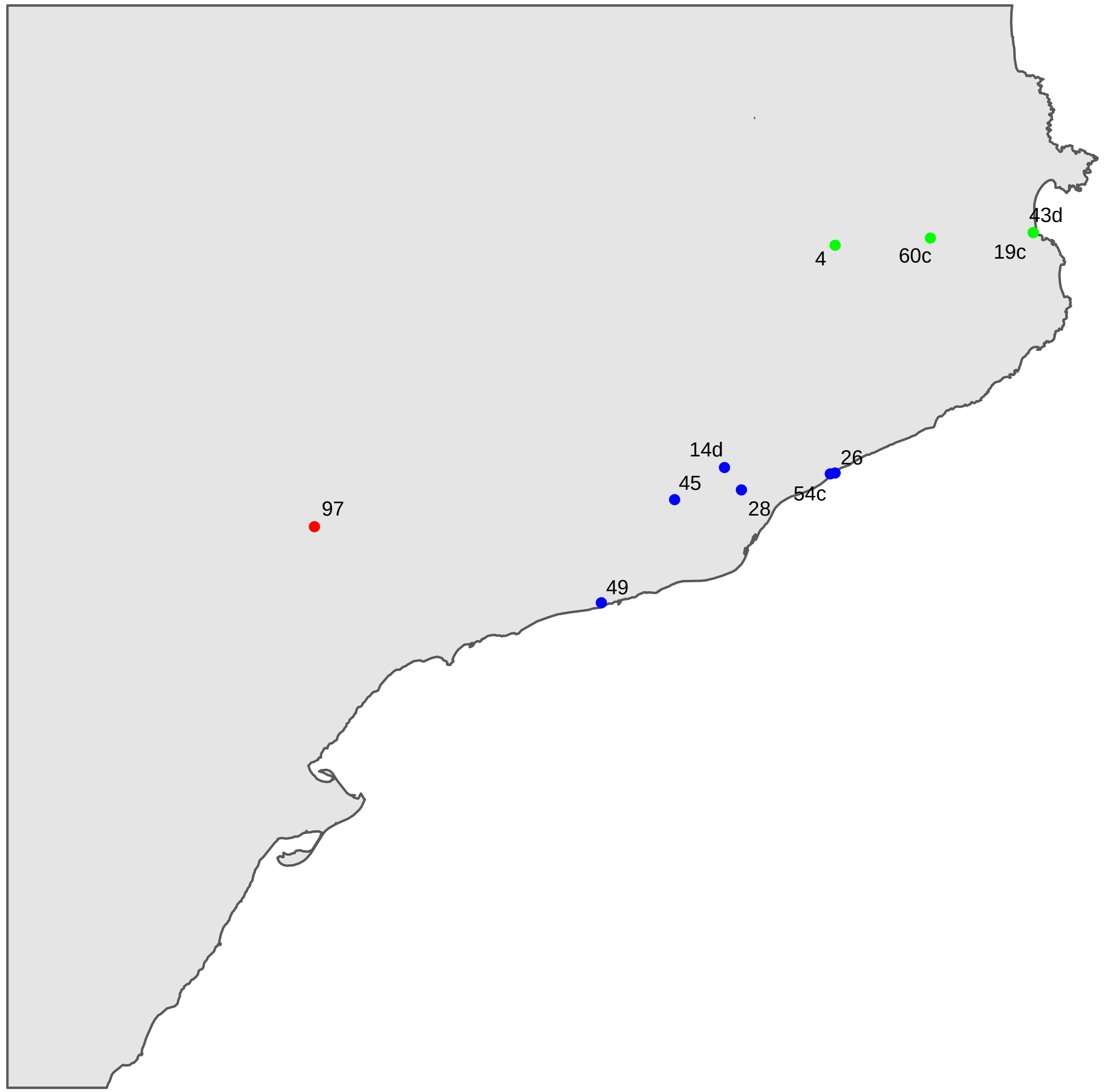

Supplement: S2 File — (PDF) [file pone.0246201.s003.pdf]
