# Supplementary figures and images for: Resilience and livestock adaptations to demographic growth and technological change: A diachronic perspective from the Late Bronze Age to Late Antiquity in NE Iberia
Source: PLoS One. 2021 Feb 17;16(2):e0246201. doi: 10.1371/journal.pone.0246201 (PMC7888671; doi:10.1371/journal.pone.0246201)

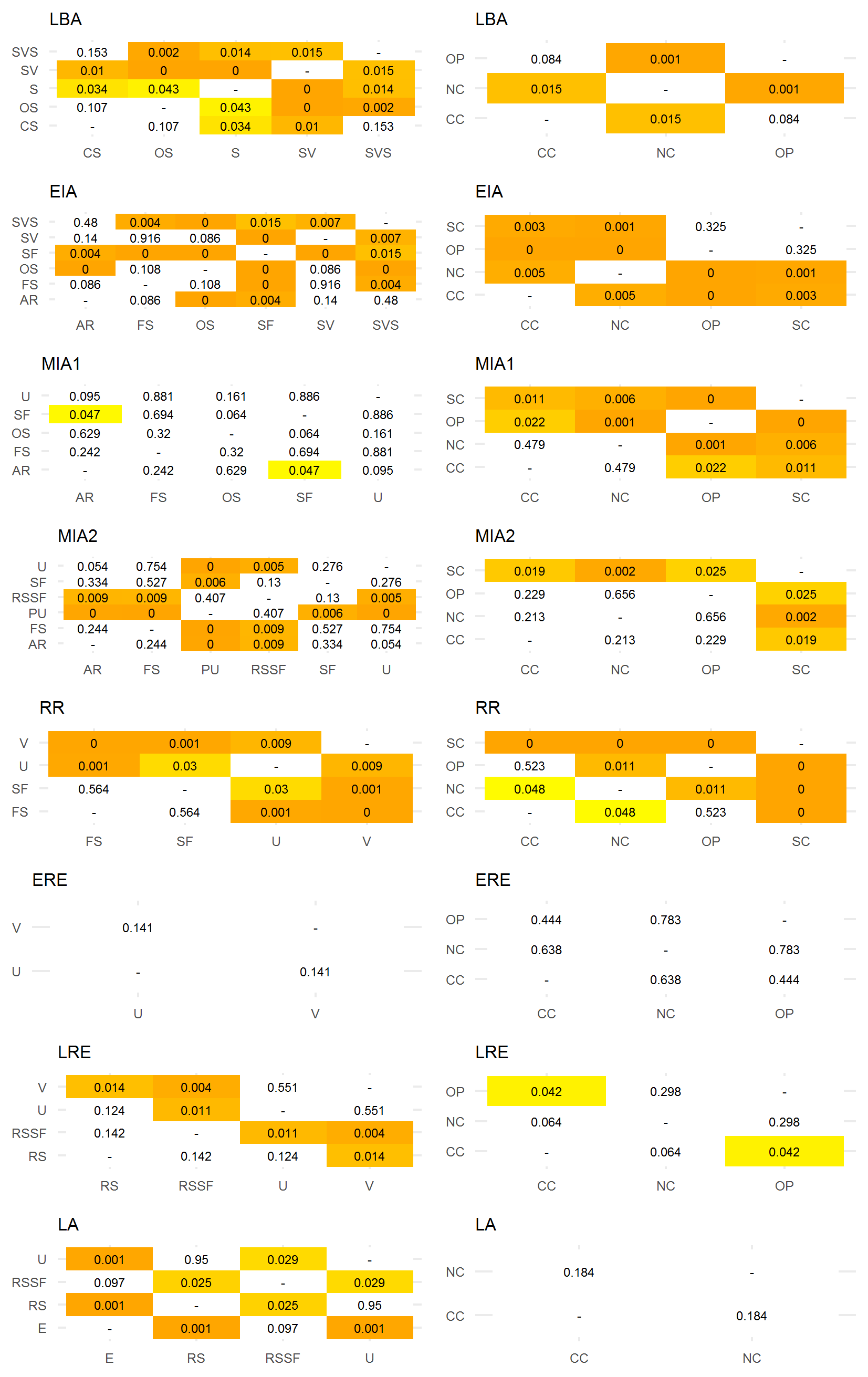

Supplement: S3 File — Chi-squared results on livestock NISP by periods, type of site (left aligned) and geographical areas (right aligned). (TIFF) [file pone.0246201.s004.tiff]
