# Supplementary material for: Resilience and livestock adaptations to demographic growth and technological change: A diachronic perspective from the Late Bronze Age to Late Antiquity in NE Iberia
Source: PLoS One. 2021 Feb 17;16(2):e0246201. doi: 10.1371/journal.pone.0246201 (PMC7888671; doi:10.1371/journal.pone.0246201)
Supplement: S6 File — (PDF) [file pone.0246201.s007.pdf]

BOTA Length

	MIA1	MIA2	RR	ERE	LRE	LA
LA	0	0	0	0	0.6	–
LRE	0.001	0.001	0.016	0.051	–	0.6
ERE	0	0	0	–	0.051	0
RR	0.003	0.057	–	0	0.016	0
MIA2	0.064	–	0.057	0	0.001	0
MIA1	–	0.064	0.003	0	0.001	0

BOTA Width

	LBA	EIA	MIA1	MIA2	RR	ERE	LRE	LA
LA	0.001	0.001	0	0	0	0.006	0.185	–
LRE	0.001	0.001	0	0	0	1	–	0.185
ERE	0	0	0	0	0	–	1	0.006
RR	0.085	0.084	0.001	0.153	–	0	0	0
MIA2	0.234	0.22	0.009	–	0.153	0	0	0
MIA1	0.712	0.894	–	0.009	0.001	0	0	0
EIA	0.955	–	0.894	0.22	0.084	0	0.001	0.001
LBA	–	0.955	0.712	0.234	0.085	0	0.001	0.001

SUDO Length

	EIA	MIA1	MIA2	RR	ERE	LRE	LA
LA	0.4	0.643	0.098	0.035	0.44	0.667	–
LRE	0.8	0.429	0.498	0.12	1	–	0.667
ERE	0.248	0.335	0.202	0.009	–	1	0.44
RR	1	0.039	0.247	–	0.009	0.12	0.035
MIA2	0.799	0.033	–	0.247	0.202	0.498	0.098
MIA1	0.262	–	0.033	0.039	0.335	0.429	0.643
EIA	–	0.262	0.799	1	0.248	0.8	0.4

SUDO Width

	EIA	MIA1	MIA2	RR	ERE	LRE	LA
LA	0.63	0.935	0.612	0.934	0.708	0.282	–
LRE	0.497	0.108	0.028	0.064	0.027	–	0.282
ERE	0.229	0.382	0.445	0.492	–	0.027	0.708
RR	0.624	0.911	0.292	–	0.492	0.064	0.934
MIA2	0.139	0.152	–	0.292	0.445	0.028	0.612
MIA1	0.543	–	0.152	0.911	0.382	0.108	0.935
EIA	–	0.543	0.139	0.624	0.229	0.497	0.63

OC Length

	LBA	EIA	MIA1	MIA2	RR	ERE	LRE	LA
LA	0.059	0.003	0.954	0.569	0.162	0.415	0.611	–
LRE	0.029	0.016	0.907	0.772	0.588	0.631	–	0.611
ERE	0.08	0.007	0.892	0.374	0.993	–	0.631	0.415
RR	0.011	0	0.681	0.187	–	0.993	0.588	0.162
MIA2	0.037	0.001	0.847	–	0.187	0.374	0.772	0.569
MIA1	0.242	0.044	–	0.847	0.681	0.892	0.907	0.954
EIA	0.795	–	0.044	0.001	0	0.007	0.016	0.003
LBA	–	0.795	0.242	0.037	0.011	0.08	0.029	0.059

OC Width

	LBA	EIA	MIA1	MIA2	RR	ERE	LRE	LA
LA	0.136	0	0	0.906	0.277	0	0	–
LRE	0.103	0	0	0.001	0.029	0.103	–	0
ERE	0.093	0	0	0	0	–	0.103	0
RR	0.23	0	0	0.214	–	0	0.029	0.277
MIA2	0.274	0	0	–	0.214	0	0.001	0.906
MIA1	0.55	0.162	–	0	0	0	0	0
EIA	0.689	–	0.162	0	0	0	0	0
LBA	–	0.689	0.55	0.274	0.23	0.093	0.103	0.136
