# Supplementary material for: Resilience and livestock adaptations to demographic growth and technological change: A diachronic perspective from the Late Bronze Age to Late Antiquity in NE Iberia
Source: PLoS One. 2021 Feb 17;16(2):e0246201. doi: 10.1371/journal.pone.0246201 (PMC7888671; doi:10.1371/journal.pone.0246201)
Supplement: S7 File — (PDF) [file pone.0246201.s008.pdf]

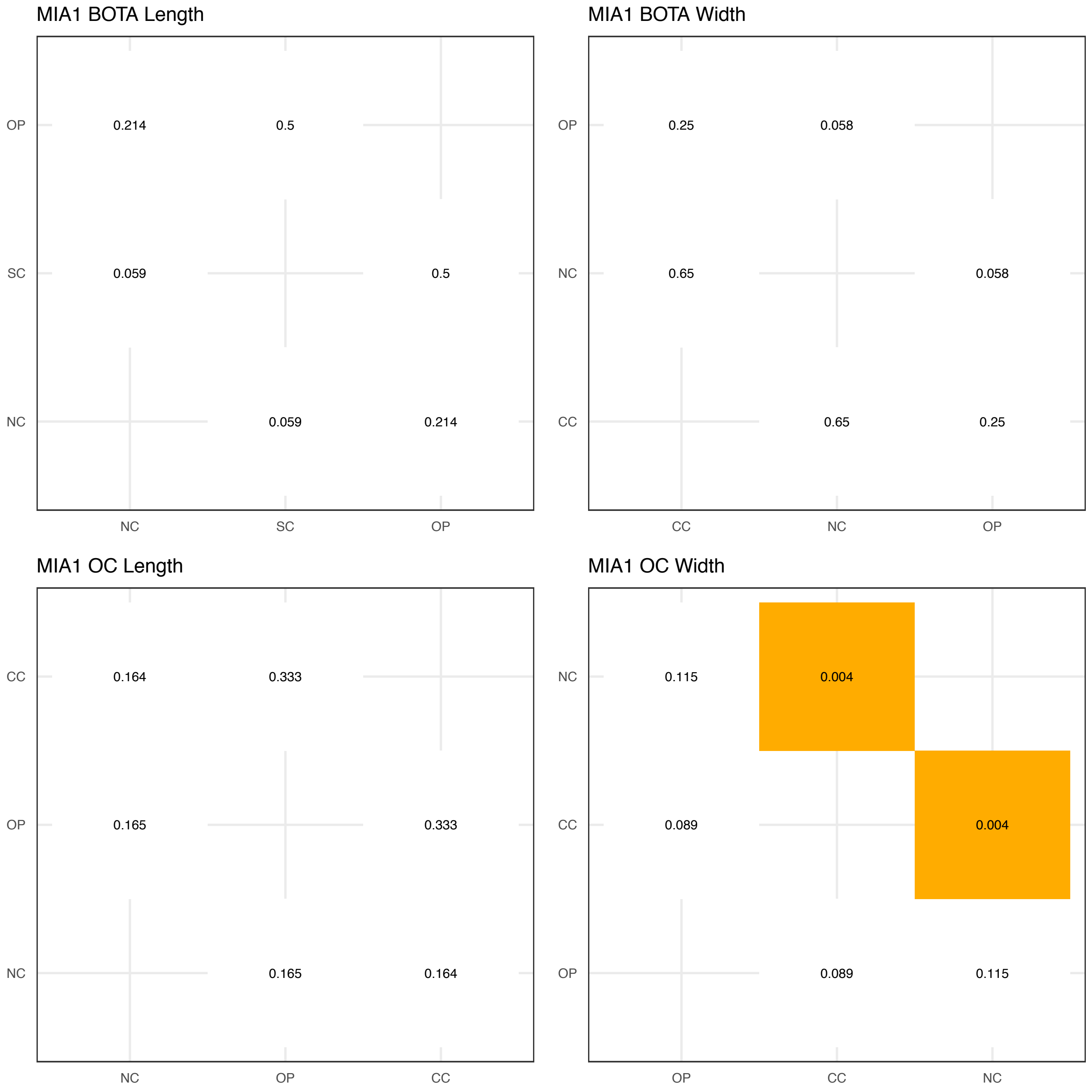
MIA1 BOTA Length
OP
SC
NC
0.214
0.5
0.059
0.5
0.059
0.214
NC
SC
OP
MIA1 BOTA Width
OP
NC
CC
0.25
0.058
0.65
0.058
0.65
0.25
CC
NC
OP
MIA1 OC Length
CC
OP
NC
0.164
0.333
0.165
0.333
0.165
0.164
NC
OP
CC
MIA1 OC Width
NC
CC
OP
0.115
0.004
0.089
0.004
0.089
0.115
OP
CC
NC

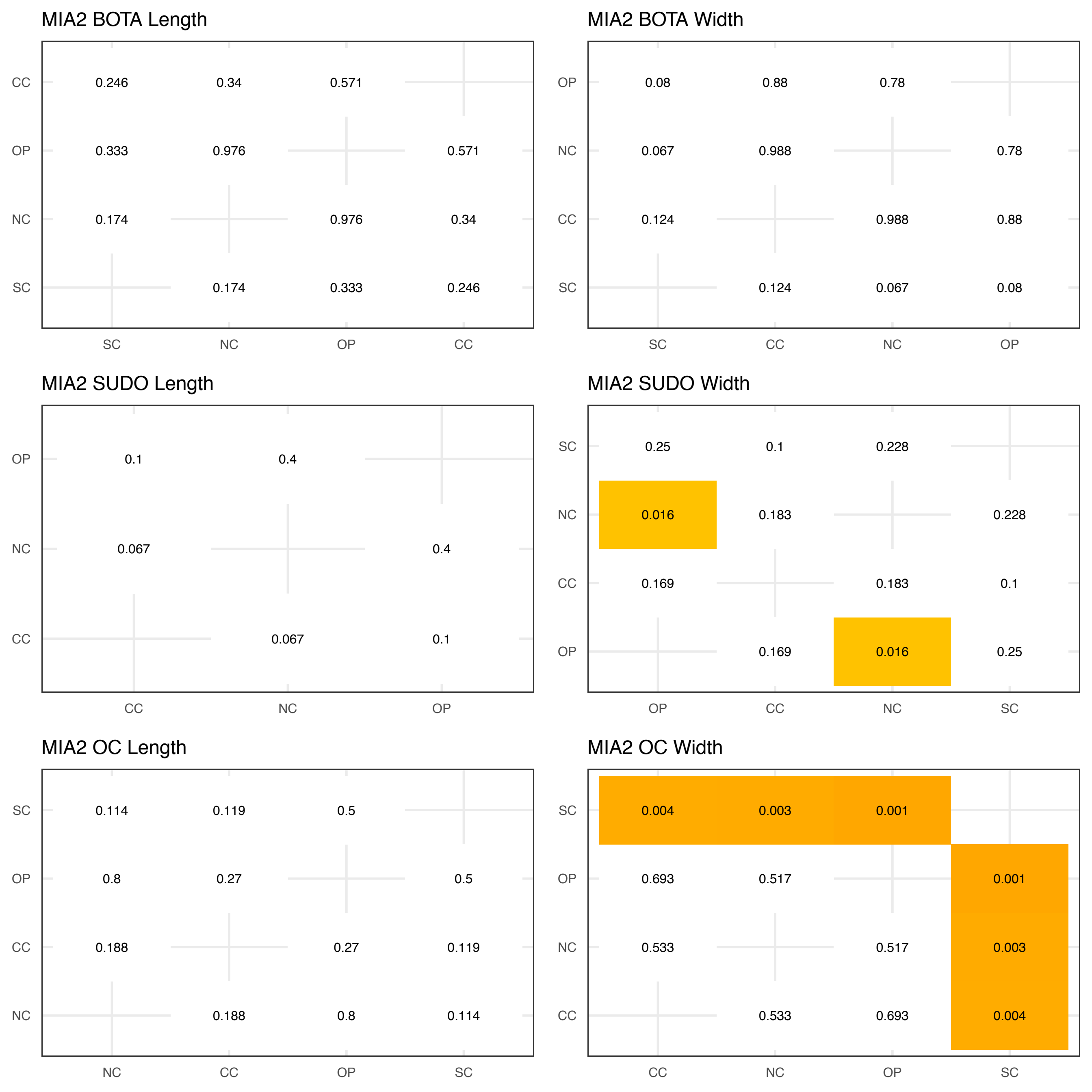
MIA2 BOTA Length
MIA2 BOTA Width
MIA2 SUDO Length
MIA2 SUDO Width
MIA2 OC Length
MIA2 OC Width
CC
OP
NC
SC
0.246
0.34
0.571
0.333
0.976
0.174
0.08
0.88
0.78
0.067
0.988
0.124
0.1
0.4
0.067
0.25
0.228
0.016
0.183
0.169
0.114
0.119
0.5
0.8
0.27
0.188
0.004
0.003
0.001
0.693
0.517
0.533

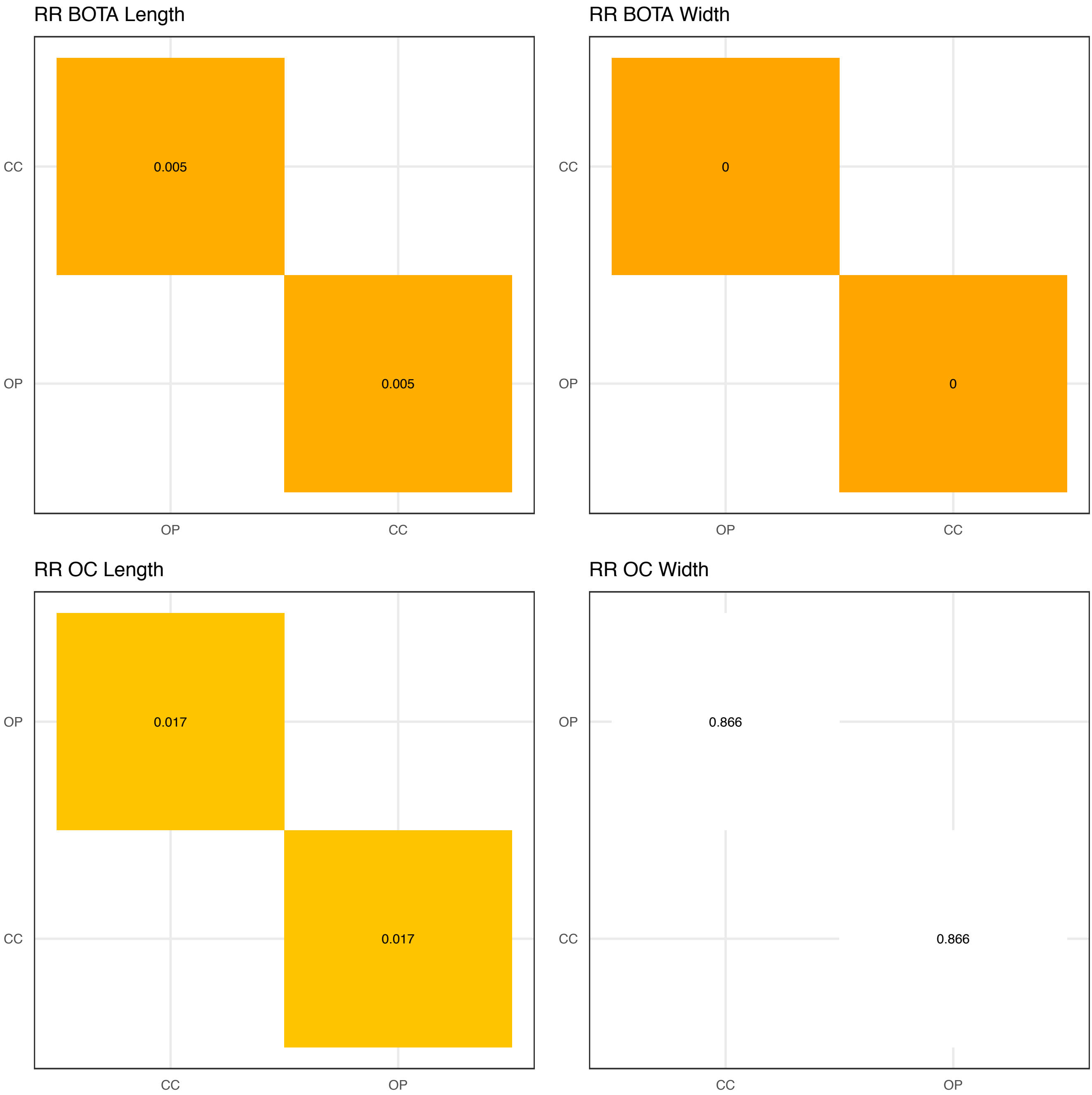
RR BOTA Length
CC
OP
0.005
0.005
OP
CC
RR BOTA Width
CC
OP
0
0
OP
CC
RR OC Length
OP
CC
0.017
0.017
CC
OP
RR OC Width
OP
CC
0.866
0.866
CC
OP

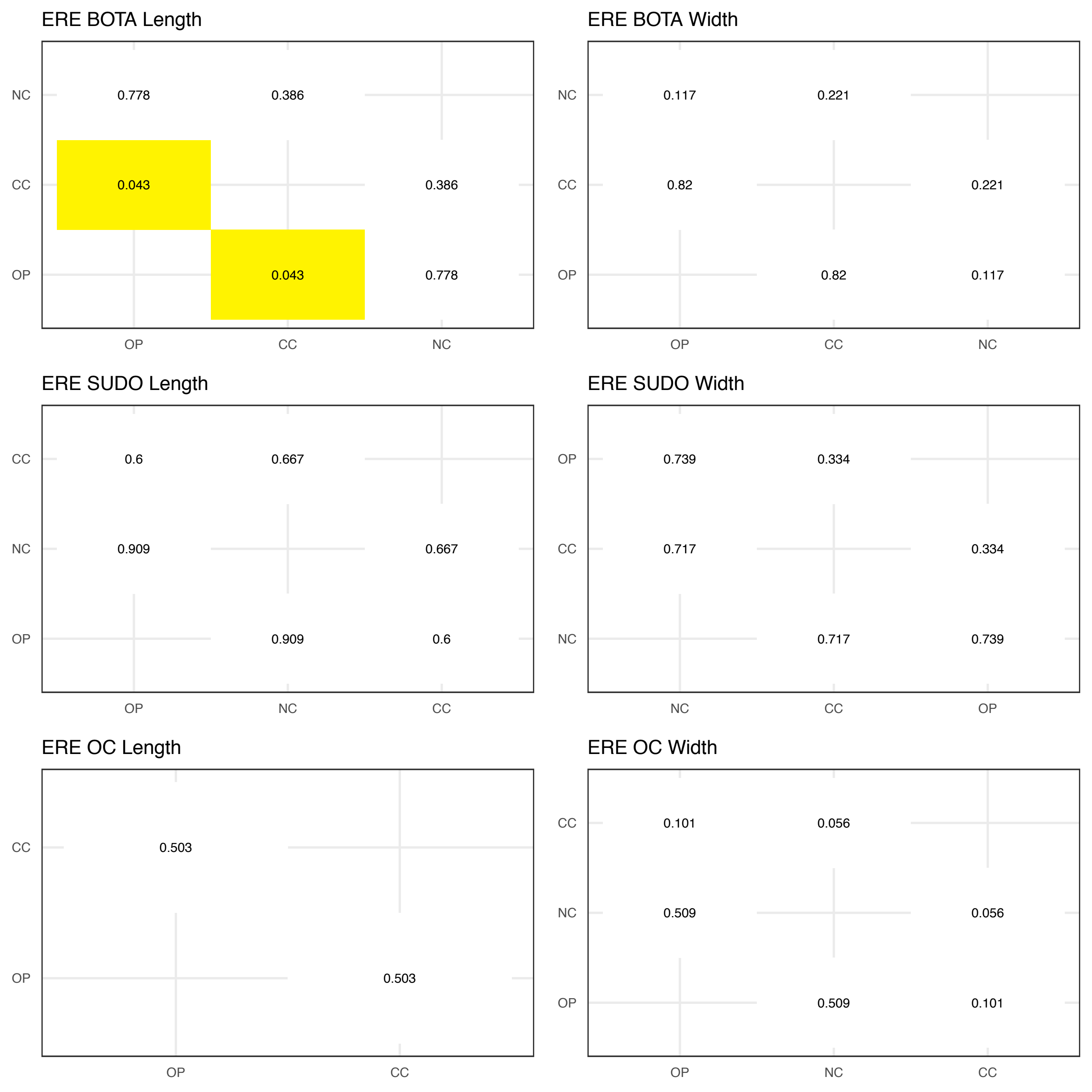
ERE BOTA Length
NC
CC
OP
0.778
0.386
0.043
0.386
0.043
0.778
OP
CC
NC
ERE BOTA Width
NC
CC
OP
0.117
0.221
0.82
0.221
0.82
0.117
OP
CC
NC
ERE SUDO Length
CC
NC
OP
0.6
0.667
0.909
0.667
0.909
0.6
OP
NC
CC
ERE SUDO Width
OP
CC
NC
0.739
0.334
0.717
0.334
0.717
0.739
NC
CC
OP
ERE OC Length
CC
OP
0.503
0.503
OP
CC
ERE OC Width
CC
NC
OP
0.101
0.056
0.509
0.056
0.509
0.101
OP
NC
CC

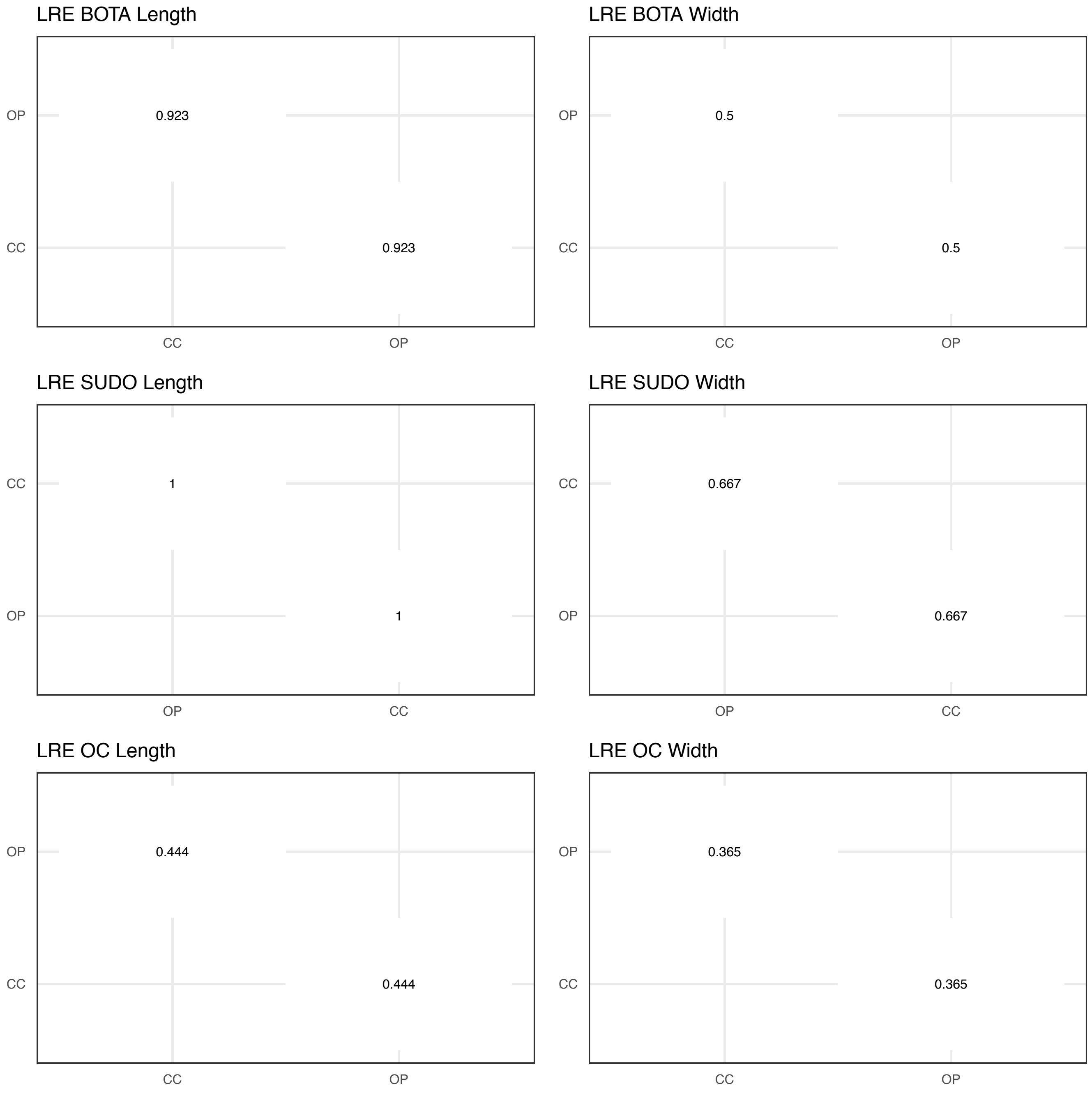
LRE BOTA Length
OP
CC
0.923
0.923
CC
OP
LRE BOTA Width
OP
CC
0.5
0.5
CC
OP
LRE SUDO Length
CC
OP
1
1
OP
CC
LRE SUDO Width
CC
OP
0.667
0.667
OP
CC
LRE OC Length
OP
CC
0.444
0.444
CC
OP
LRE OC Width
OP
CC
0.365
0.365
CC
OP

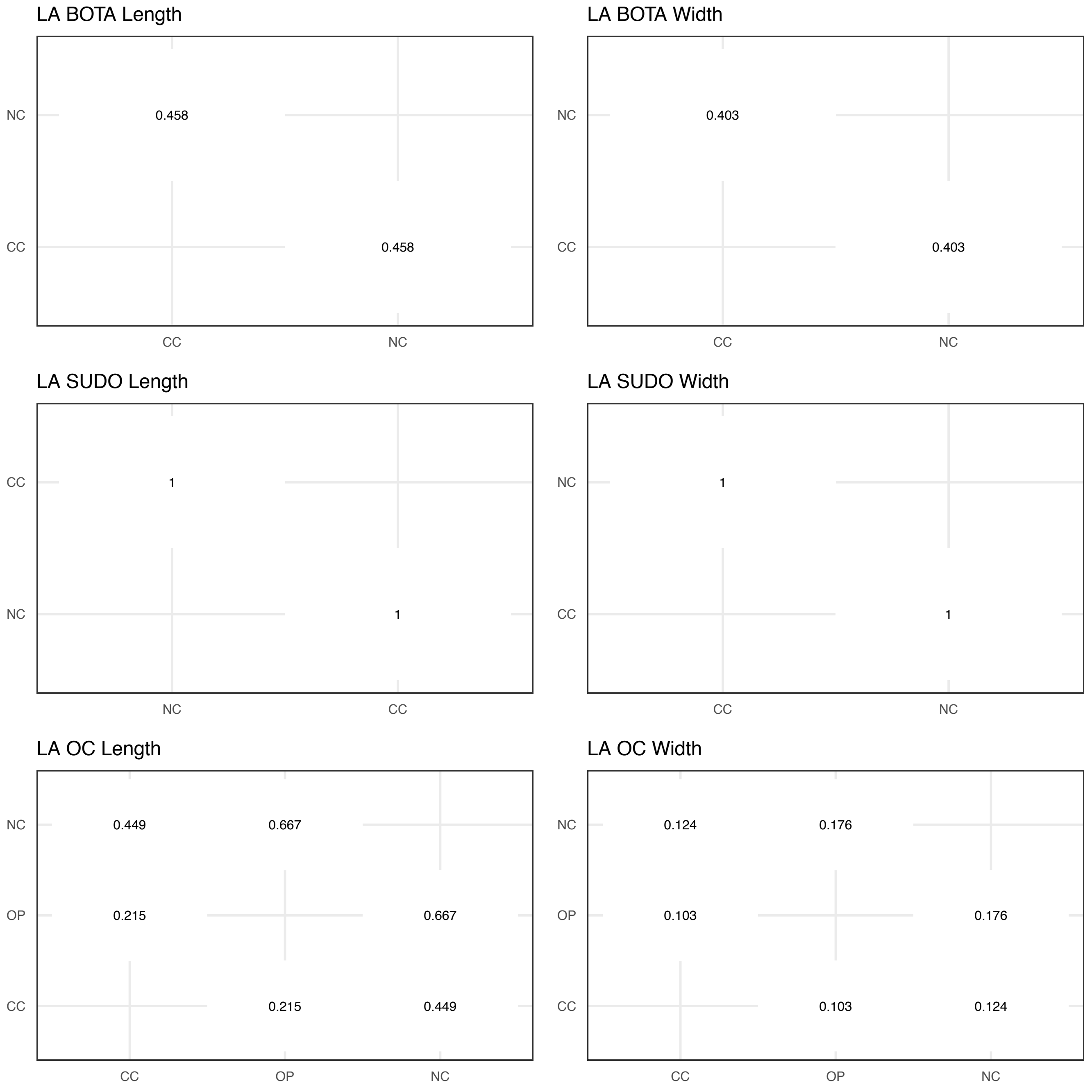

LA BOTA Length
NC
CC
0.458
0.458
CC
NC
LA BOTA Width
NC
CC
0.403
0.403
CC
NC
LA SUDO Length
CC
NC
1
1
NC
CC
LA SUDO Width
NC
CC
1
1
CC
NC
LA OC Length
NC
OP
CC
0.449
0.667
0.215
0.667
0.215
0.449
CC
OP
NC
LA OC Width
NC
OP
CC
0.124
0.176
0.103
0.176
0.103
0.124
CC
OP
NC
